# Quantitative integrative taxonomy informs species delimitation in *Teloschistaceae* (lichenized* Ascomycota*): the genus *Wetmoreana* as a case study

**DOI:** 10.1186/s43008-024-00140-1

**Published:** 2024-04-01

**Authors:** Karina Wilk, Robert Lücking

**Affiliations:** 1 W. Szafer Institute of Botany, Polish Academy of Sciences, Lubicz 46, 31–512 Kraków, Poland; 2 Botanischer Garten und Botanisches Museum Berlin, Freie Universität Berlin, Königin-Luise-Strasse 6–8, 14195 Berlin, Germany

**Keywords:** Phylogenetic binning, MRPP, PCA, South America, 6 New species, 8 New combinations

## Abstract

The genus *Wetmoreana* was studied using quantitative integrative taxonomy methods to resolve the genus delimitation and explore its taxonomy diversity at the species level. As a result, the genus *Fulgogasparrea* is synonymized with *Wetmoreana*, and the latter includes 15 formally described species, one subspecies, and three further, thus far undescribed species: *W. appressa*, *W. awasthii* comb. nov., *W. bahiensis* sp. nov., *W. brachyloba* comb. nov., *W. brouardii*, *W. chapadensis* comb. nov., *W. circumlobata* sp. nov., *W. decipioides*, *W. intensa* comb. nov., *W. ochraceofulva* comb. nov., *W. rubra* sp. nov., *W. sliwae* sp. nov., *W. sliwae* ssp. *subparviloba* subsp. nov., *W. subnitida* comb. nov., *W. texana*, and *W. variegata* sp. nov. Eleven of 19 examined taxa are newly placed within this genus or confirmed to belong to it. Two species, *W. awasthii* and *W. intensa*, are transferred to *Wetmoreana* without additional analysis but based on previous studies. The *W. brouardii* and *W. ochraceofulva* species complexes are discussed in detail*.* Additionally, *Caloplaca muelleri* and *C. rubina* var. *evolutior* are transferred to *Squamulea*, and the latter is elevated to the species rank.

**Supplementary Information:**

The online version contains supplementary material available at 10.1186/s43008-024-00140-1.

## Introduction

*Teloschistaceae* is one of the families of lichenized *Ascomycota* where internal genus-level classification is most contentious, although, with the exception of some questionable studies based on demonstrably chimeric data, subfamily divisions are stable (Arup et al. 2013; Bungartz et al. 2020; Wilk et al. 2021). Many new genera have been described in recent years, with the total reaching more than 90 (71 listed in Wijayawardene et al. 2022, with a further 23 treated as synonyms or possible synonyms of accepted genera), including about one thousand known species (Arup et al. 2013), resulting in a ratio of 11 species per genus. In more speciose families, the number of accepted genera is comparatively lower: 2765 species and 77 genera in *Parmeliaceae* (36 species per genus), 2161 species and 79 genera in *Graphidaceae* (27 species per genus), and 943 species and 43 genera in *Verrucariaceae* (22 species per genus) (Lücking et al. 2017).

Genus-level classification in *Teloschistaceae* is particularly challenging due to the lack of clear correlations of potentially diagnostic characters with phylogenetically defined clades, along with a high level of morphological homoplasy, particularly across lineages containing crustose taxa formerly placed in the collective genus *Caloplaca* (Gaya et al. 2008; Arup et al. 2013; Bungartz et al. 2020). Consequently, accurate placement of taxa at generic level without molecular data is difficult or often impossible (e.g., taxa with the ‘*Flavoplaca citrina* morphology’; Vondrák et al. 2009; Wilk et al. 2021). It is, however, unclear whether such diagnostic characters are indeed absent or have not been properly assessed using quantitative approaches (Vondrák et al. 2013). Although morphology is often integrated into phylogenetic revisions at the family level, e.g., in *Collemataceae* (Otálora et al. 2013), *Graphidaceae* (Rivas Plata et al. 2012a), *Pannariaceae* (Ekman et al. 2014), *Parmeliaceae* (Thell et al. 2012), or *Ramalinaceae* (Kistenich et al. 2018), morphological data are less frequently quantitatively assessed in the form of a matrix or using statistical methods (e.g., Kistenich et al. 2018).

Some exceptions are found in cladistic approaches to assess morphological data, e.g., in the order *Arthoniales* (Tehler 1990), the families *Gomphillaceae* (Lücking et al. 2005) or *Parmeliaceae* (Saag and Randlane 1995; Saag et al. 2002), or the genus *Diploschistes* Norman in *Graphidaceae* (Lumbsch and Tehler 1998). When complementing molecular studies, the power of quantitative approaches to properly delimit and define taxa becomes apparent, e.g., in the *Graphidaceae* (Parnmen et al. 2012; Rivas Plata et al. 2012a; Lücking et al. 2015; Lücking and Kalb 2018) or in the *Arthoniales* (Perlmutter et al. 2020). Crustose *Teloschistaceae* have been subjected to quantitative phenotypic analysis within a phylogenetic framework (Frolov et al. 2016), using a comprehensive character and data matrix established by Vondrák et al. (2013); however, the applied technique has been limited to morphometric (continuous) data.

Here, we use the recently established genus *Wetmoreana* as an example to test the combination of phenotype-based phylogenetic binning (PBPB) and multiple response permutation procedure (MRPP) to assess species and genus boundaries and to clarify the nomenclature within this complex. *Wetmoreana* belongs to subfamily *Teloschistoideae*, with *W. texana* as its type (Arup et al. 2013). Almost at the same time when the genus was established, Kondratyuk et al. (2013) considered it polyphyletic and proposed its division into *Fulgogasparrea* and *Wetmoreana* s.str. However, that division has not been broadly accepted (Lücking et al. 2017; Bungartz et al. 2020; Wilk et al. 2021; Wijayawardene et al. 2022). *Wetmoreana* sensu Arup et al. (2013) is a relatively small group consisting of four accepted species, besides *W. texana* also *W. appressa*, *W. brouardii*, and *W. decipioides*, plus several additions proposed in the present study. The relocation of *Xanthoria tenax* within *Wetmoreana* by Kondratyuk et al. (2013) was based on misidentified material representing an undescribed species of *Wetmoreana* (Kondratyuk et al. 2020). Kondratyuk et al. (2013, 2015) circumscribed *Fulgogasparrea* as a genus including three of the four species originally assigned to *Wetmoreana*, keeping the latter restricted to its type, *W. texana*, and later adding also *F. awasthii* (Mishra et al. 2020). A further species, *F. intensa*, was recently described from Brazil (Aptroot et al. 2021). Species of *Wetmoreana* (including *Fulgogasparrea*) form marginally lobate (placodioid) or squamulose thalli with anthraquinones and often produce vegetative propagules, such as isidia, papillae, schizidia or soredia. Apothecia are less common or unknown, e.g. in *W. decipioides* (Arup et al. 2013). The species are exclusively saxicolous, occurring mostly on siliceous rocks. Most *Wetmoreana* species, including those added in the present study, occur in South America, a few in North America (U.S.A., Mexico), and a one each in Africa and Asia (Arabian Peninsula and South Korea) (Arup et al. 2013; this study). The origin of the genus is unknown, but the highest diversity occurs in South America and may indicate this continent as the centre of its diversification.

The objective of the present study included: (i) assess specific and intraspecific delimitation of *Wetmoreana* species based on phenotype and DNA-based phylogenetic reconstructions and multi-response permutation procedure (MRPP), (ii) evaluate potentially diagnostic characters for *Wetmoreana* at the genus level using phenotype-based phylogenetic binning (PBPB), (iii) assess the validity of the separation of *Fulgogasparrea* through molecular data and MRPP, (iv) assess the potential phylogenetic position of several South American putative *Wetmoreana* species for which no sequence data are unavailable by PBPB, and (v) analyze phenotypic variation within the newly recognized *W. ochraceofulva/ W. variegata* complex in Africa, the Arabian Peninsula and South America using MRPP. Based on our results, the genus *Fulgogasparrea* is synonymized with *Wetmoreana*, six new taxa are established, namely *W. bahiensis* sp. nov., *W. circumlobata* sp. nov., *W. rubra* sp. nov., *W. sliwae* sp. nov., *W. sliwae* subsp. *subparviloba* subsp. nov., and *W. variegata* sp. nov., and further six species are transferred to *Wetmoreana*: *Caloplaca brachyloba*, *C. chapadensis*, *C. ochraceofulva*, *C. subnitida*, *F. awasthii*, and *F. intensa*. We further transfer *Caloplaca muelleri* and *C. rubina* var. *evolutior* to *Squamulea*, and *C. rubina* var. *evolutior* is elevated to species rank. Two larger subclades within *Wetmoreana* are recognized: the *W. brouardii* clade, including *W. appressa*, *W. bahiensis* sp. nov., *W. brouardii*, *W. intensa* comb. nov. and *W. subnitida* comb. nov., and the *W. ochraceofulva* clade, including *W. ochraceofulva* comb. nov. and *W. variegata* sp. nov.

## Material and methods

### Taxon sampling

The study was based on the herbarium material from B, BCN, BM, C, E, F, G, GZU, KoLRI, KRAM, LPB, LD, M, MIN, S, UPS, and the private herbarium of U. Arup. The material originates mainly from South America, in some cases from other continents, including also types and original collections. The studied samples were represented by marginally lobate, squamulose or rarely areolate species of *Wetmoreana* and the following morphologically similar genera: *Aridoplaca*, *Calogaya*, *Cinnabaria*, *Gyalolechia*, *Squamulea*, *Teuvoahtiana*, and selected representatives of *Caloplaca* s. lat. representing all three subfamilies within *Teloschistaceae*. Among the studied material, we included poorly known neotropical *Caloplaca* species (e.g., *C. brachyloba*, *C. chapadensis*, *C. muelleri* and *C. rubina*), which were revised using type or original material. A large collection of *W. ochraceofulva* from Africa, the Arabian Peninsula and South America (BM, E, G, KRAM, LD, and MIN) was studied in detail.

### Phenotype assessment

**Fig. 1:**
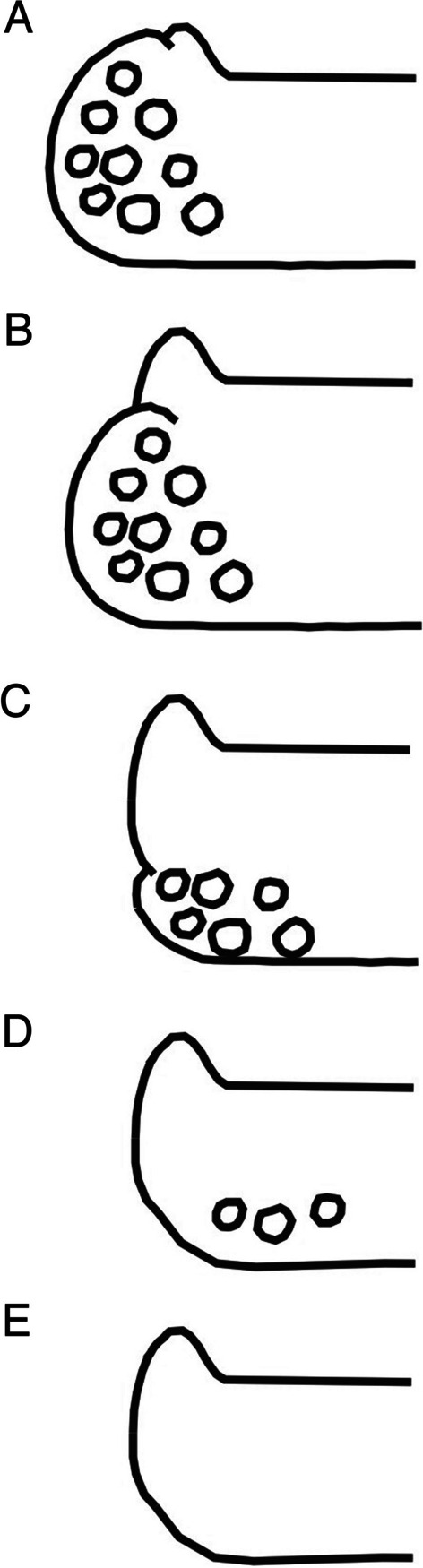
Stage of development of the thalline margin in the zeorine apothecia: **A** ± persistent, **B** partly reduced, **C** much reduced, and two other types of apothecia **D** pseudolecanorine and **E** biatorine not producing thalline margins

Morphological characters were studied on herbarium material using a Nikon SMZ 1270 dissecting microscope (Tokyo, Japan). Anatomical details of thalli, apothecia and pycnidia were examined using hand-cut sections mounted in water under a Nikon Eclipse 50i compound microscope. Ascospore sizes are given as an average with standard deviation and extremes are given in brackets, with the number of measurements (n) and specimens examined (N) also indicated in brackets. Spores were measured without preheating the slides (a method used in Wetmore 2003). Conidia were measured based on calibrated photographs of preparations. In case of lobate species, only terminal lobes were measured, and lobe length refers to the distance from the tip of the lobe back to the first branch (Wetmore and Kärnefelt 1998). The widths of lobes were measured below their tips (cf. Brodo et al. 2001: Fig. 3). Apothecial margins were measured according to Vondrák et al. (2013: Fig. 3). Apothecium types follow Clauzade and Roux (1985: Fig. 61), where 1–2 refer to immersed apothecia, 3 to erumpent apothecia, and 4–5 to sessile apothecia. The thalline margin in zeorine apothecia was classified as ± persistent, partly reduced, or much reduced (Fig. 1). The presence of crystals on anatomical structures was observed under polarized light and the solubility of crystals were determined using 25% KOH (K), 65% nitric acid (N), 5% acetic acid (abbreviated as AA), and 2% hydrochloric acid (HCl). Calcium oxalate crystals (CaOx crystals) are defined as insoluble in K, soluble in N, insoluble in AA and soluble in HCl without giving off gas (Yasue 1969; Wilk et al., in prep.). To study the occurrence of crystals within the thallus, especially in *W. ochraceofulva* and similar species, cross sections of thalline lobes were prepared by selecting those which overlap other lichen thalli where possible, which allowed to observe the delimited layer of CaOx crystals. Hydrochloric acid was used to test for the presence of calcium carbonate (CaCO_3_) in rock substrata. Terminology of thallus and apothecial structures follows Bungartz (2002) and Ryan et al. (2002, 2012). Photographic documentation was made with a Nikon DS-Fi2 digital camera combined with the imaging software NIS-Elements D 4.30 (Nikon Corporation, Japan). All specimens examined were photographed and the photo documentation was used to compare species and obtain dimensions of structures that were difficult to measure under the microscope, such as conidia.

In total, 112 specimens were included in the phenotype data matrix, representing 31 taxa (Additional file 1: Table S1). For 39 specimens the ITS sequences are available, which constitute 35% of all studied specimens. These specimens provide the basis for the identification of the reference taxa and were used to generate the ‘molecular reference tree’ for the phenotype-based phylogenetic binning analysis. The remaining 73 specimens (65%), for which ITS sequences are unknown, constitute ‘taxon queries’. The latter group includes among others the poorly known, mostly neotropical species, such as *Blastenia fernandeziana* f. *validior*, *Caloplaca brachyloba*, *C. chapadensis*, *C. muelleri*, *C. rubina* var. *evolutior*, and *C. xanthobola.* They were chosen for comparison with representatives of *Wetmoreana* due to their morphological similarities to *Wetmoreana* spp., and were considered to probably belong to this genus. The 73 phenotypic characters, which comprehensively characterizes the studied group, were used in analyses, including 59 qualitative and 14 morphometric characters. Among them, 32 characters belong to thallus morphology; 2 to thallus anatomy; 13 to apothecia morphology; 9 to apothecia anatomy; 2 to pycnidia morphology; and 6 to conidia anatomy; 6 to CaOx crystals occurrences; and 1 to habitat preferences. The binary and ordered multistate characters were evaluated, measured and coded (Additional file 2: Table S2).

The "query taxa" represented *Teloschistaceae* which morphologically, anatomically, and geographically relate to the study fresh material from South America and include putative undescribed *Wetmoreana* species. They also include some poorly examined tropical *Teloschistaceae* for which DNA sequences are unavailable. The chosen reference taxa encompass species for which DNA sequences are available and which have morphological and anatomical resemblance with *Wetmoreana*, e.g. *Aridoplaca*, *Calogaya*, *Cinnabaria*, *Gyalolechia*, *Squamulea*, and *Teuvoahtiana*. Other placodioid or squamulose genera, such as *Austroplaca*, *Follmannia*, *Gondwania*, *Orientophila*, *Polycauliona*, *Scutaria*, *Sirenophila*, *Xanthopeltis*, where not considered, as they deviate in important features from *Wetmoreana* and geographic distribution (e.g., endemic to Australasia) and/or ecology (e.g., restricted to seashore rocks).

### Molecular phylogenetic analyses

The monophyly of the *Wetmoreana* genus was demonstrated in previous papers (Arup et al. 2013; Søchting et al. 2014; Wilk et al. 2021). To assess phylogenetic relationships between of all available *Wetmoreana* spp., we first used a three-marker data set (ITS, nuLSU, mtSSU), for which additional DNA sequences were downloaded from GenBank (Additional file 1: Table S1). *Teloschistes flavicans* was chosen as an outgroup because it is not closely related to *Wetmoreana* and is located in a sister clade to that genus (Arup et al. 2013: Fig. 1). Sequences of two specimens of *W. ochraceofulva* from Africa and Saudi Arabia were newly generated following the methods outlined in Wilk et al. (2021) (Additional file 1: Table S1), assembled and edited using CodonCode Aligner 6.0.2, and subjected to BLAST queries for an initial verification of their identities. Alignments of the three different markers were assembled using MAFFT v. 7 (Katoh et al. 2019), and then corrected manually in AliView 1.17.1 (Larsson 2014). They were first analysed separately to check for potential incongruence. The one incongruence was present in the ITS analysis in relation to the other two (see chapter Results for details). The concatenated alignment of the three markers was assembled, with 34 terminals and a length of 2980 bases (ITS: 623, nuLSU: 1476, mtSSU: 881). The alignment was subjected to maximum likelihood (ML) analyses in RAxML v. 8.2.0 (Stamatakis 2014) under the GTR-Gamma model. Given the low number of terminals and the high number of characters, no partitioning scheme was applied, as it would not result in any notable differences. Branch support was assessed by non-parametric bootstrapping with 100 replicates. Phylogenetic trees were visualized and edited in FigTree v. 1.4.4 (Rambaut 2018). Nodes with bootstrap values ≥ 70 were considered as supported.

As a second step, phylogenetic relationships of *Wetmoreana* with the morphologically similar genera *Aridoplaca*, *Calogaya*, *Cinnabaria*, *Gyalolechia*, *Squamulea*, *Teuvoahtiana*, and the species *Caloplaca fernandeziana*, were assessed based on the ITS marker, following the methods outlined above, with the corresponding additional ITS sequences downloaded from GenBank (Additional file 1: Table S1). The resulting ITS alignment included 39 terminals and was 650 bases long. Given previously published phylogenies, the topology was internally rooted with subfamily *Xanthorioideae*, defining the clade formed by *Teuvoahtiana altoandina*, *Squamulea* spp., and *Calogaya* spp. as outgroup. The resulting phylogenetic tree was consistent with previously published phylogenies (e.g., Arup et al. 2013) and was used as the molecular reference tree for the downstream PBPB analysis.

The alignments for all phylogenetic analyses performed are available in Additional files 3–6: FILES S1–S4.

### Phenotype-based phylogenetic binning (PBPB)

PBPB was carried out following Berger et al. (2011) and Lücking et al. (2015). The procedure was performed in three steps: (i) computing the molecular reference trees (ITS rDNA marker) for the terminals for which sequence data were available; (ii) performing weight calibration for the phenotype characters of these same terminals, using maximum likelihood (ML) and maximum parsimony (MP); and (iii) performing the binning analysis using the Evolutionary Placement Alogarithm (EPA), under both parsimony and maximum likelihood weights for the remaining terminals for which no DNA sequences where available. All steps were performed in RAxML. During the binning procedure, alternative placement of each terminal based on its phenotype characters were being assessed through non-parametric bootstrapping using 100 replicates. The resulting classification trees were visualized using FigTree, with some file modifications following Lücking et al. (2015), and the classification tables were edited for display in Microsoft Excel. Results were summarized as cartoon trees.

The final phenotype data matrix was used to run PCA ordinations (Principal Component Analysis) to test how well the separation of clades defined by molecular phylogeny and morphological binning corresponded to discrimination of the included species by means of morphological data. PCA analysis was performed in PC-ORD v. 5.03 (McCune and Mefford 2011; McCune et al. 2002) using 108 specimens of all studied taxa of *Wetmoreana* and other genera, and separately using 73 specimens of *Wetmoreana* exclusively. The morphological data included 71 and 63 variables respectively; missing data, mostly of apothecia, were interpolated, and *Callopisma subnitidum* (syntype, PBPB no. 49), *Calogaya biatorina* (PBPB no. 127) and *W. decipioides* (PBPB no. 70 and 122), were not included in the PCA analyses because of missing data of apothecia/pycnidia. PCA was done using correlation to compute the cross-products matrix, and a randomization test was performed using 999 replicates.

### Multi-response permutation procedure (MRPP)

**Fig. 2:**
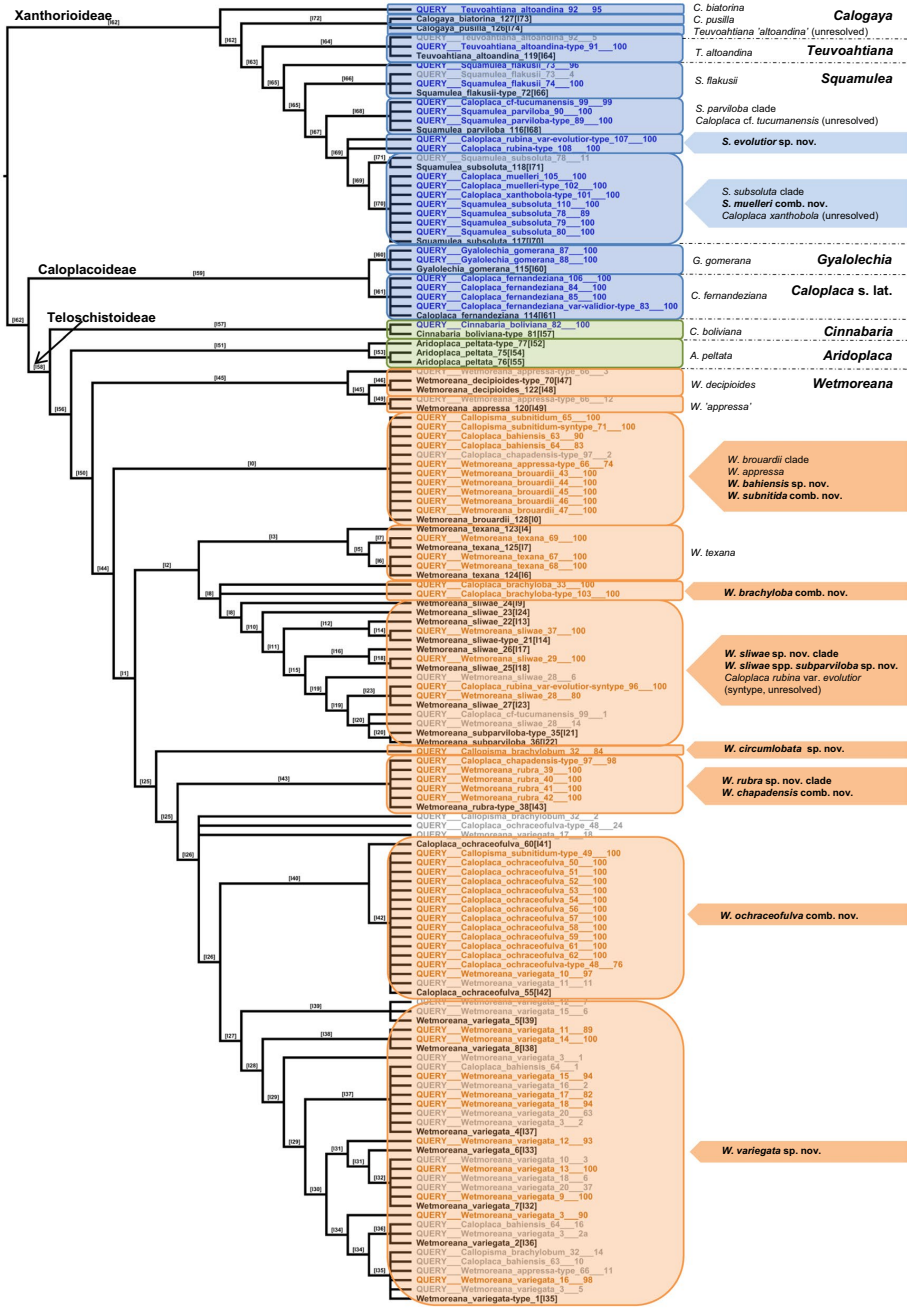
Summary tree showing placements of 73 queried *Teloschistaceae* specimens for which the DNA sequences are unknown, using 73 phenotypic traits for 112 specimens in total, based on PBPB and ML weighting technique. Numbers in square brackets indicate node numbers and numbers after the query name indicate bootstrap support for a particular placement of the query taxon based on its morphological features. The numbers following the species names are a unique PBPB number. The colors used on the figure indicate subfamilies as follows: *Xanthorioideae* and *Caloplacoideae* in blue, *Teloschistoideae* including *Wetmoreana* in orange and the *Cinnabaria*–*Aridoplaca* complex in green. Species names in black without the prefix "QUERY" are the reference taxa for which DNA sequences were available. The remaining terminals, all with the prefix "QUERY", represent the query taxa, the gray-colored terminals indicating bootstrap support (in cases where the same individual was binned into several, alternative clades). Clade designations are summarized on the right side of the tree, with newly recognized species in bold

MRPP was performed in PC-Ord 5.03 following Parnmen et al. (2012), with the objective to test for the statistically significant phenotypic differences between predefined groups (clades). The groups constituted our initial taxon delimitation hypotheses from the previous binning analysis. The clades tested were of two types, namely the clades defined by molecular data only, and the extended clades produced by adding the terminals binned to each molecular clade with support, according to ML and MP weighting techniques separately. At first, we focused on the phylogenetically defined complexes comparison, to decide how many species to distinguish within. Two complexes: *ochraceofulva*/ *variegata* (= *W. ochraceofulva* clade) and *sliwae*/ *subparviloba*/ *brachyloba* (= *W. sliwae* clade), corresponding to ML and MP classification trees, were analysed (Fig. 2, Additional file 7: Fig. S1). The test was performed for complete and incomplete datasets; the latter were devoid the apothecial characters which were missing in some specimens, especially in case of *W. ochraceofulva* clade, but also in the case of *W. texana* and *W. brouardii*. For the complete dataset, missing data were interpolated from other specimens, the type material when possible. However, in case of *W. decipioides* and *Callopisma subnitidum* (syntype, PBPB no. 49), none of the specimens studied produced apothecia, and these species were analysed only in case of the incomplete dataset.

In case of the cross-species comparison three grouping scenarios were employed: (i) *W. ochraceofulva* vs. *W. variegata*, (ii) *W. sliwae* vs. *W. sliwae* ssp. *subparviloba*, and (iii) *W. sliwae* vs. *W. brachyloba*. In case of the *W. ochraceofulva* clade, both dataset types (with and without apothecia) were used. In the last comparison, *W. sliwae* s. lat. and *W. sliwae* s.str. [without *W. sliwae* ssp. *subparviloba* and *C. rubina* var. *evolutior* no. 96 (syntype)] were compared with *W. brachyloba* separately. The additional MRPP analysis was made to assess *Squamulea subsoluta* vs. *S. evolutior* comb. nov (PBPB no. 107–108), as they are similar and closely related taxa on the classification trees. The PCA analyses were done in all cases to check the phenotypic variation between the studied taxa.

**Fig. 3:**
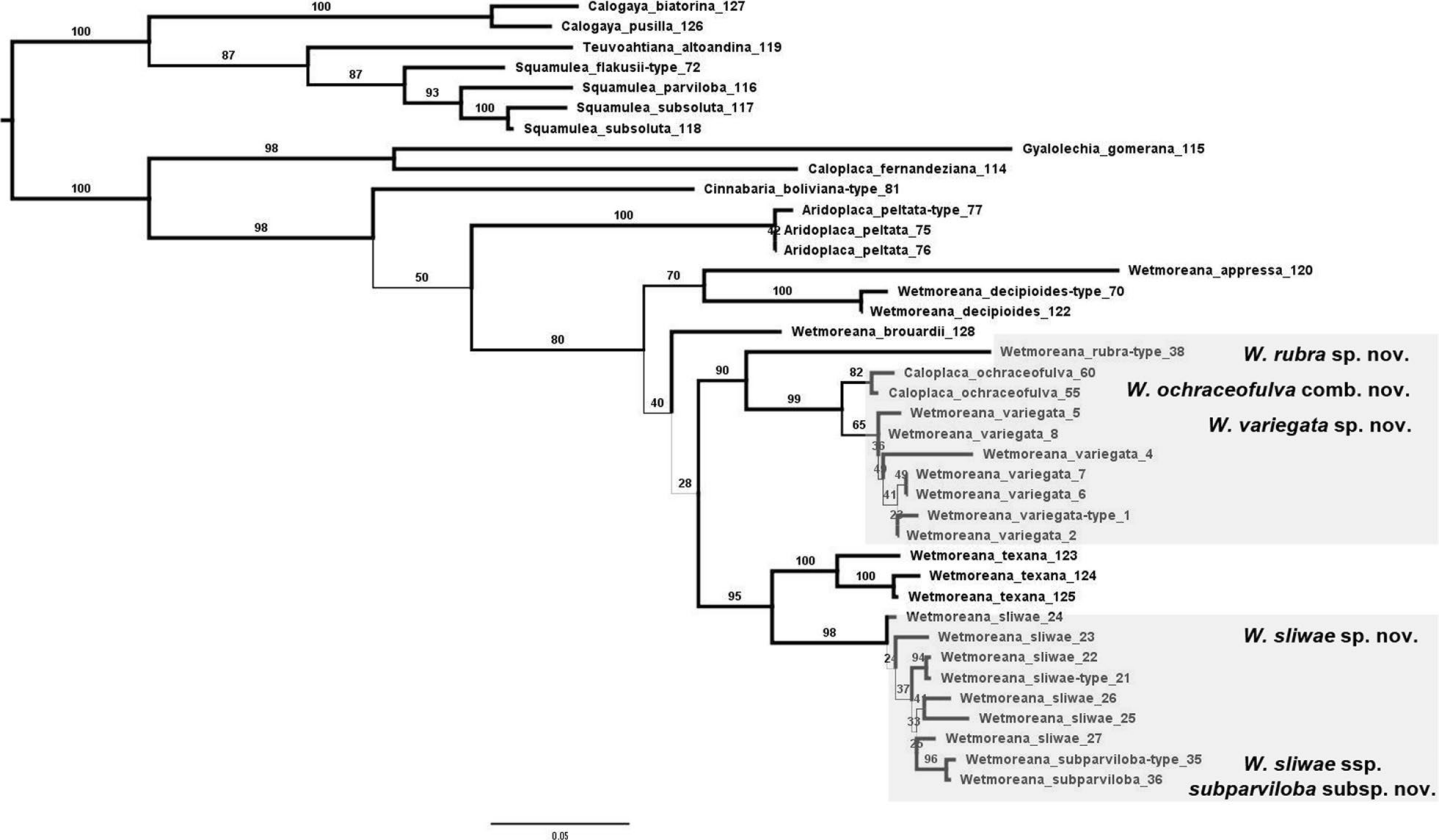
Overall molecular phylogeny of the members of *Wetmoreana* and additional genera *Aridoplaca*, *Calogaya*, *Cinnabaria*, *Gyalolechia*, *Squamulea*, *Teuvoahtiana*, and the species *Caloplaca fernandeziana* which members are similar to *Wetmoreana*. Phylogenetic ITS tree was performed in RAxML. The support values associated with branches indicate maximum likelihood bootstrap values. The values ≥ 70 are considered as significant support. Newly proposed taxa are marked. The numbers after species names corresponds to PBPB numbers

Finally, we tested the separation of *Fulgogasparrea* from *Wetmoreana* as proposed by Kondratyuk et al. (2013). We defined the clades as follows: 1) *W. decipioides* (type species for *Fulgogasparrea*) and *W. appressa*, and 2) *W. brouardii*, *W. ochraceofulva*/ *W. variegata*/ *W. rubra*, and *W. texana* (type species for *Wetmoreana*)/ *W. sliwae/ W. sliwae* ssp. *subparviloba*, based on Fig. 3. The test was performed based on specimens for which both molecular and phenotypic datasets were present, with the exception of *W. brouardii* which was supplemented by an additional sample lacking sequence data. Additionally, to increase sample size, the analysis was repeated on an expanded dataset with additional binned specimens of each species. Because *W. decipioides* is sterile, the incomplete datasets were used in both analyses.

## Results

### Molecular phylogenetic analysis

The *Wetmoreana* clade consisted of 33 terminals, clustering into five main, strongly supported subclades and two singleton lineages: the *W. ochraceofulva*/ *W. variegata*, *W. brouardii/ W. intensa*, *W. texana*/ *W. sliwae*, *W. decipioides* and an unnamed subclade, with two additional singleton lineages representing *W. ‘appressa’* and *W. rubra*. The relationships among the five subclades and the singleton lineages were not supported in the three-locus phylogeny (Additional file 8: Fig. S2). *Wetmoreana texana* was fully supported as sister to the newly described *W. sliwae*, which in turn included the newly recognized *W. sliwae* subsp. *subparviloba* in a nested position. *Wetmoreana ochraceofulva* and *W. variegata* were recovered as sister on short stem branches, and each of the species-level clades was only moderately supported. The recently described *W. intensa* was recovered as sister to *W. brouardii*. Finally, *W. rubra* was recovered on a long, separate branch, and the species is clearly divergent from other members of the genus. Its phylogenetic position was not congruent in the individual gene trees, forming a supported sister group relationship with the *W. ochraceofulva*/ *W. variegata* clade in the ITS tree, but with the *W. texana*/ *W. sliwae* clade in the other trees (Additional files 9–11: Figs. S3–S5). Fortunately, this supported conflict did not interfere with the objectives of the study regarding the potential delimitation of *Fulgogasparrea* vs. *Wetmoreana* or species delimitation in this group. *Wetmoreana ‘appressa’* lies within the genus clade on a very long branch. Depending on the analyses it was recovered as sister to *W. decipioides* (Fig. 3) or was not grouped with any other clade (Additional files 8–9: Figs. S2–S3).

### Phenotype-based phylogenetic binning analysis (PBPB)

The analyses were performed to classify the twelve South American putative *Wetmoreana* species for which sequences were unavailable (Additional file 1: Table S1), and thus to define the initial taxon delimitation hypothesis subsequently tested in the MRPP analyses, in order to determine diagnostic vs. homoplastic characters for *Wetmoreana*. The twelve putative *Wetmoreana* species included *B. fernandeziana* f. *validior*, *Callopisma subnitidum* (syntype; currently *W. ochraceofulva*), *Caloplaca bahiensis*, *C. brachyloba*, *C. chapadensis*, *C. muelleri*, *C. rubina* var. *evolutior*, *C. rubina* var. *evolutior* (syntype; currently *W.* cf. *sliwae*), *C.* cf. *tucumanensis*, *C. subnitida*, and *C. xanthobola*, as well as one undescribed species (*Caloplaca* sp. 1). Given that at least some of these species may belong in other genera, we expanded the PBPB reference set by six additional genera represented by both molecular and morphological data, namely *Aridoplaca*, *Calogaya*, *Cinnabaria*, *Gyalolechia*, *Squamulea*, and *Teuvoahtiana*, plus a sequenced specimen of *Caloplaca fernandeziana* (Additional file 1: Table S1, Fig. 3).

Phylogenetic binning of the chosen twelve *Caloplaca* s.lat. species for which no molecular data are available, based on ML weighting, suggested placement of seven species in *Wetmoreana*, whereas the remaining five species were suggested to not form part of *Wetmoreana*, nested instead within *Squamulea* or clustering with *Caloplaca fernandeziana* (one species, i.e. *B. fernandeziana* f. *validior*). MP weighting placed the same seven species in *Wetmoreana*, whereas three of the remaining species were placed outside *Wetmoreana*, in the same positions as with ML weighting; the positions of the remaining two query samples were not supported when using MP weighting (Table 1, Additional file 12: Table S3, Fig. 2, Additional file 7: Fig. S1).

**Table 1:** Placements of twelve query *Caloplaca* s.lat. species within studied clades based on phenotype-based phylogenetic binning method under ML and MP weighting. The numbers in brackets indicate the number of specimens per species studied. The proposed placement of the query taxa means that species is the closet species with respect to morphological characters but not necessarily that it belongs to that species

Query species	ML	MP
*Blastenia fernandeziana* f. *validior* (1)	*C. fernandeziana*	*C. fernandeziana*
*Callopisma subnitidum* syntype (1)	*Wetmoreana ochraceofulva*	*W. ochraceofulva*
*Caloplaca bahiensis* (2)	*W. brouardii*	*W. variegata*
*C. brachyloba* (2)	*W. sliwae/*separate taxon	*W. sliwae/*separate taxon
*C. chapadensis* (1)	*W. rubra*	*W. rubra*
*C. muelleri* (2)	*Squamulea subsoluta*	*S. parviloba*
*C. rubina* var. *evolutior* (2)	*S. subsoluta/*separate taxon	unsupported
*C. rubina* var. *evolutior* syntype (1)	*W. sliwae*	*W. sliwae*
*C. subnitida* (2)	*W. brouardii*	*W. brouardii*
*C.* cf. *tucumanensis* (1)	*S. parviloba*	*S. parviloba*
*C. xanthobola* (1)	*S. subsoluta*	unsupported
*Caloplaca* sp. 1 (1)	*W. rubra*/separate taxon	*W. variegata*

Additional 14 query specimens of other *Teloschistaceae* for which molecular data at the species level are generally available, and which included in the reference tree, mostly grouped with the corresponding species. Two exceptions were found for *W. appressa* and *Teuvoahtiana altoandina*. In the case of *W. appressa*, the non-sequenced type specimen did not cluster with the sequenced specimen bearing that identification but grouped with *W. brouardii* instead under ML weighting, BS = 74 (MP weighting did not give support). This implies that the sequenced specimen labelled *W. appressa* in GenBank may not represent that species and is more likely an undescribed species within *Wetmoreana.* In the case of *Teuvoahtiana altoandina*, only MP weighting grouped together all specimens of this species (Additional file 7: Fig. S1), the alternative placement suggesting that *T. altoandina* (PBPB no. 92) might represent an undescribed species probably belonging in the genus *Calogaya* (Fig. 2). Notably, the thirteen *W. ochraceofulva* query specimens, including the type, grouped only with one of the two sequenced specimens of that species, i.e. *W. ochraceofulva* PBPB no. 55 from Saudi Arabia, but not with *W. ochraceofulva* PBPB no. 60 from Namibia. Even if quite similar, none of the *W. ochraceofulva* query specimens grouped with *W. variegata*, suggesting that the subtle differences between the two taxa are diagnostic. The type specimen of *W. ochraceofulva* from Africa and syntype of *Callopisma subnitidum* from South America were shown to be conspecific, both grouping with the sequenced *W. ochraceofulva* from Saudi Arabia (Additional file 12: Table S3, Fig. 2, Additional file 7: Fig. S1).

The phylogenetic binning method also allows assessment of clade-based correlations, and hence the potentially diagnostic importance of morphological characters through the ML and MP weighting process. Of the 73 characters, 34 received more-or-less significant weighting scores (from 70 to 100) in both ML and MP analyses. The remaining 30 characters received higher weights only in one analysis. Among them, 23 were weighted higher by the ML and seven by the MP analysis. Nine characters received low weights (< 70) in both analyses (Additional file 2: Table S2). Characters with high weight in both analyses included the type of thallus morphology (e.g., areolate, squamulose), the morphology of the squamules (their margins and their spatial growth), the presence of propagules, their type and way of formation, the presence and color of the prothallus, the type of apothecia, the apothecial disc color and pruinosity, the structure of the parathecium and the ratio of parathecium and amphithecium, the ratio of length and width of ascospores, the shape of the conidia, and the presence of CaOx crystals in the thalline cortex and in the inner part of the apothecia (parathecium and basal part). Characters with higher weights in only one of the two approaches encompassed, e.g., the type of substrate, the shape of areoles, the size and shape of marginal lobes, the presence of the necral layer and the continuity of the algal layer in the thallus, the frequency of apothecia, their diameter and degree of emergence, the visibility of both apothecial margins, the development of thalline margin of apothecia, the length of ascospores, the length of conidia, and the ratio of length and width of conidia. Characters with low weights in both analyses included the shape of apothecia, the continuity of the apothecial disc surface, the color of the apothecial margin, the width of parathecium, the width of ascospores, the thickness of the acospore septum, the ratio of ascospore length and septum width, the frequency of pycnidia, and the width of conidia (Additional file 2: Table S2). In summary, 57 out of 73 characters received significant weight scores in ML. Among them, qualitative traits accounted for 50 (85% of all qualitative traits), and morphometric traits accounted for 7 (50% of all morphometric traits). In contrast, 41 characters received support in MP and 37 were qualitative traits (63% of all qualitative traits) and 4 were morphometric traits (29% of all morphometric traits).

### Ordination of morphological dataset

**Fig. 4:**
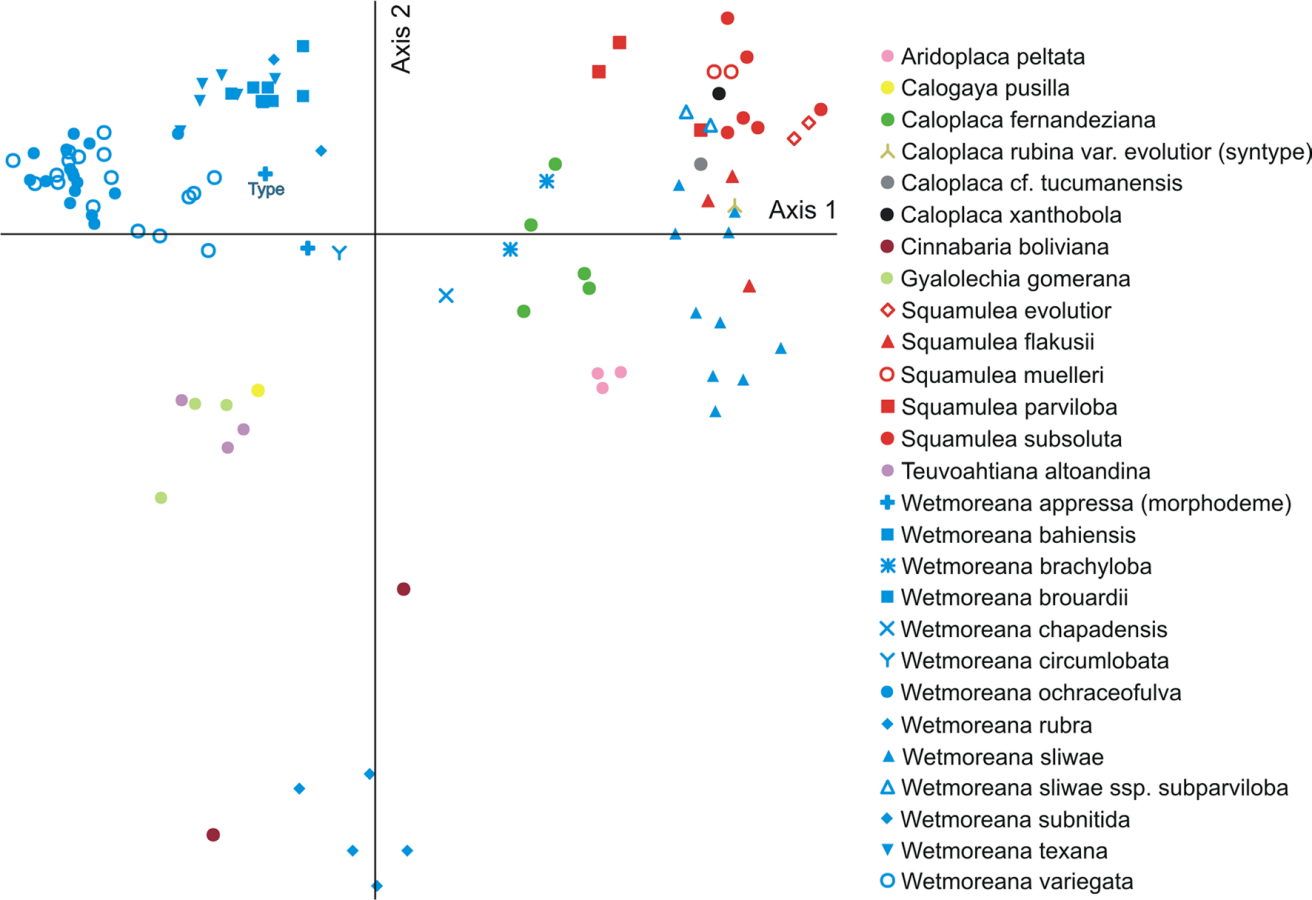
PCA ordination based on 71 phenotypic characters of 27 species of *Wetmoreana* and other genera included in the study

In the extended PCA including 27 species representing all study genera, the lobate members of *Wetmoreana* occupied the upper left quadrant of the diagram and, surprisingly, did not group with other lobate taxa such as *Calogaya*, *Gyalolechia* and *Teuvoahtiana*. In contrast, the squamulose *W. sliwae* fell together with squamulose representatives of *Squamulea* (Fig. 4). The sublobate *W. rubra* and *Cinnabaria boliviana*, characterized by the same ecology, took isolated positions in the diagram (Fig. 4), supporting the molecular data indicating these taxa as more divergent lineages (Fig. 3; Wilk et al. 2021: Fig. 2). *Wetmoreana ochraceofulva* and *W. variegata* overlapped morphologically, whereas the remaining lobate species, *W. appressa*, *W. bahiensis*, *W. brouardii*, *W. texana*, and *W. subnitida*, were more-or-less separate from each other. The two samples of *W. appressa* appeared to be not conspecific. The type specimen associated with other lobate *Wetmoreana* species, but the second sample, *W. ‘appressa’*, together with *W. circumlobata* fell near the centre of the diagram. *Wetmoreana chapadensis*, which forms an obscurely lobate thallus and additional prothallus, had a relatively isolated position relative to other lobate *Wetmoreana* species. *Wetmoreana brachyloba* was resolved as morphologically close to but still separate from *W. sliwae*. The latter cluster included *W. sliwae* ssp. *subparviloba* and *Caloplaca rubina* var. *evolutior* (syntype). *Squamulea muelleri* comb. nov. and *S. evolutior* came out as morphologically close to *Squamulea subsoluta*, and *Caloplaca* cf. *tucumanensis* and *C. xanthobola* also fell within *Squamulea*.

The restricted PCA focusing on the 14 *Wetmoreana* species recognized in this study (Additional file 13: Fig. S6) showed some differences compared to the more extended PCA. Above all, in this analysis, *W. ochraceofulva* and *W. variegata* no longer overlapped morphologically. In contrast, *W. appressa*, *W. bahiensis*, *W. brouardii*, *W. subnitida*, and *W. texana* more-or-less overlapped with *W. variegata*, corresponds to the high variation of the latter taxon. *Wetmoreana ‘appressa’*, *W*. *circumlobata* and especially *W. chapadensis* remained as clearly morphologically distinct from the other lobate *Wetmoreana* species.

### Multi-response Permutation Procedure (MRPP) analysis

The MRPP analyses were performed only on selected scenarios, excluding some clades that were resolved as clearly distinguished in the PCA analysis, namely *W. bahiensis* (PBPB no. 63), *W. circumlobata* (PBPB no. 32), and *C. xanthobola* (Additional files 14 and 15: Figs. S7 and S8). In contrast, *Caloplaca rubina* var. *evolutior* (syntype; PBPB no. 96) nested within the *W. sliwae* clade and was treated as conspecific with the latter based on the PCA (Additional file 16: Fig. S9).

**Fig. 5:**
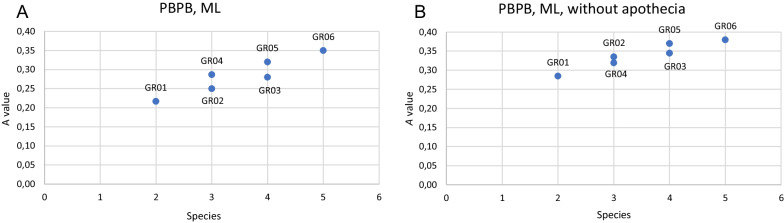
The comparison between the *ochraceofulva*/ *variegata* and *sliwae*/ *subparviloba*/ *brachyloba* complexes using MRPP based on PBPB ML results for complete (**A**) and incomplete (without apothecia, **B**) datasets. The analyzed groups are as follow, GR01: *ochraceofulva* s.lat. vs. *sliwae* s.lat., GR02: *ochraceofulva* s.lat. vs. *sliwae* s.lat./*brachyloba*, GR03: *ochraceofulva* s.lat. vs. *sliwae*/*subparviloba*/*brachyloba*, GR04: *ochraceofulva*/*variegata* vs. *sliwae* s.lat., GR05: *ochraceofulva*/*variegata* vs. *sliwae* s.lat./*brachyloba*, GR06: *ochraceofulva*/*variegata* vs. *sliwae*/*subparviloba*/*brachyloba*. *A* value means within-group agreement value

The MRPP gave the highest A value (between 0.35 and 0.4) when all clades were treated as separate taxa and the lowest (between 0.2 to 0.3) when the two larger complexes, the *ochraceofulva*/ *variegata* clade and the *sliwae*/ *subparviloba*/ *brachyloba* clade, were treated as two more broadly defined taxa (Fig. 5, Additional file 17: Fig. S10a, b, c). The cross-species MRPP indicated the absence of significant differences between the *W. ochraceofulva* and the *W. variegata* clades, suggesting that these species should be treated as a single taxon (Table 2). The analysis gave weak support for distinguishing both taxa when apothecia and more samples were included (A = 0.1), but only two samples were found fertile out of 15 specimens of *W. ochraceofulva*, and seven out of 19 specimens in *W. variegata*, leading to the observed uncertainty of the results. PCA ordination supported the separation of both taxa also when apothecia were included (Additional file 14: Fig. S7), although this effect was not seen when PCA was performed on all genera studied (Fig. 4).

**Table 2:** MRPP results for pairwise species comparisons using clades defined from molecular data or extended clades including terminals binned to each molecular clade with support by ML and MP weighting. Chance-corrected within-group agreement (A) describes within-group homogeneity, compared to the random expectation. When all items are identical within groups A = 1 (the highest possible value for A). If heterogeneity within groups equals expectation by chance, then A = 0. If there is less agreement within groups than expected by chance, then A < 0 (McCune et al. 2002)

Pairwise species comparisons	DNA	PBPB, ML	PBPB, MP
*W. ochraceofulva*–*W. variegata*	A = 0.03731636 p = 0.15359669	A = 0.11654641 p = 0.00000010	A = 0.13915435 p = 0.00000000
*W. ochraceofulva*–*W. variegata* (without apothecia)	A = –0.01825464 p = 0.61452644	A = 0.06007533 p = 0.00002964	A = 0.07058784 p = 0.00000499
*W. sliwae*–*W. sliwae* ssp. *subparviloba*	A = 0.24269049 p = 0.00614222	A = 0.14099005 p = 0.00210686	A = 0.14099005 p = 0.00210686
*W. sliwae* s.l.–*W. brachyloba*	N/A	A = 0.12262540 p = 0.00289011	A = 0.12262540 p = 0.00289011
*W. sliwae* s.str.^1^–*W. brachyloba*	N/A	A = 0.18061758 p = 0.00258934	A = 0.18061758 p = 0.00258934
*S. subsoluta* s.l.–*S. evolutior*	N/A	A = 0.10107775 p = 0.00502788	A = 0.10107775 p = 0.00502788
*S. subsoluta* s.str.^2^–*S. evolutior*	N/A	A = 0.11600227 p = 0.00901012	A = 0.11600227 p = 0.00901012

^1^*Wetmoreana sliwae* s.str. without ssp. *subparviloba* and *C. rubina* var. *evolutior* PBPB no. 96 (syntype)

^2^*Squamulea subsoluta* s.str. without *S. muelleri*

The separation of *W. sliwae* and *W. sliwae* ssp. *subparviloba* on one hand and between *W. sliwae* and *W. brachyloba* on the other was supported by both MRPP (Table 2) and PCA ordination analyses (Fig. 4, Additional file 16: Fig. S9). Finally, the MRPP supported the distinction of *Squamulea subsoluta* and *S. evolutior*, although these taxa overlapped on the PCA diagram (Table 2, Additional file 15: Fig. S8).

The separation of *Fulgogasparrea*, defined as including only *W. decipioides* and *W. ‘appressa’* to reflect reciprocal monophyly, from the other three clades (*brouardii*, *ochraceofulva-variegata-rubra*, *texana-sliwae-subparviloba*; Fig. 3) not obtained support with the results A = 0.03435718, p = 0.06735338.

## Discussion

*Wetmoreana* is placed in *Teloschistoideae*, where it constitutes a well-separated lineage. Its phylogenetic relationship with other closely related genera is resolved without support (Arup et al. 2013; Wilk et al. 2021). The delimitation of *Wetmoreana* as proposed by Arup et al. (2013) is confirmed in this study, in contrast to the delimitations of this group presented by Kondratyuk et al. (2013, 2015). According to the latter authors, *Wetmoreana* is polyphyletic, comprising two generic clades, namely *Fulgogasparrea* and *Wetmoreana* s.str. Following this concept, the first taxon includes *W. decipioides* (type), *W. ‘appressa’*, *W. awasthii*, *W. brouardii,* and *W. intensa*, while the genus *Wetmoreana* includes only the type species, *W. texana*. Our phylogenetic analyses based on the extended datasets of the *Wetmoreana* clade show that *Fulgogasparrea* sensu Kondratyuk et al. cannot be molecularly defined because it would be highly paraphyletic (Fig. 3, Additional file 8: Fig. S2). We performed, the MRPP analyses to test a possible separation of *Fulgogasparrea* (including *W. decipioides* and *W. ‘appressa’* only) from the rest of the molecularly defined clades (the *W. brouardii*, *W. ochraceofulva*, and *W. texana* clades) based on their morphological variation and using the one-loci molecular phylogeny as framework (Fig. 3). The MRPP results did not show significant phenotypic differences between *Fulgogasparrea* and the remaining clades (A = 0.03435718, p = 0.06735338). Therefore, we synonymize *Fulgogasparrea* with *Wetmoreana*, and transfer *F. awasthii* and *F. intensa* to *Wetmoreana*. Unfortunately, we could not resolve the placement of *W. awasthii* within *Wetmoreana*, as the authors of the combination of that species into *Fulgogasparrea* (Mishra et al. 2020) failed to deposit the underlying sequence data, ignoring agreed standards of open and fair access to data.

Currently, *Wetmoreana* includes 15 formally described species, one subspecies, and three further, yet undescribed species. These are *W. appressa*, *W. awasthii*, *W. bahiensis*, *W. brachyloba*, *W. brouardii*, *W. chapadensis*, *W. circumlobata*, *W. decipioides*, *W. intensa*, *W. ochraceofulva*, *W. rubra*, *W. sliwae*, *W. sliwae* ssp. *subparviloba*, *W. subnitida*, *W. texana*, and *W. variegata*. Eleven of the 19 tested taxa are newly placed within this genus or confirmed to belong to it based on quantitative integrative taxonomy methods. Two species (*W. awasthii* and *W. intensa*) are transferred to *Wetmoreana* without additional analysis, based on previous studies only (Mishra et al. 2020; Aptroot et al. 2021). *Wetmoreana* species are exclusively saxicolous, crustose, more-or-less lobate at the margins (placodioid), or rarely squamulose. The squamulose representatives, namely *W. brachyloba*, *W. sliwae,* and *W. sliwae* ssp. *subparviloba*, are similar to the unrelated genus *Squamulea* (subfamily *Xanthorioideae*), which differs from *Wetmoreana* mainly in the paraplectenchymatous apothecial exciple, the frequent presence of a prothallus, and shorter ascospores [8–15 µm vs. (9)12–22 µm; based on examined samples]. In contrast, the crustose-lobate (placodioid) *Wetmoreana* species are similar to many other lobate taxa of different genera widely dispersed in all three *Teloschistaceae* subfamilies. Surprisingly, the studied lobate members of *Calogaya*, *Gyalolechia*, and *Teuvoahtiana* are clearly separated from the lobate members of *Wetmoreana* in the PCA ordination diagram (Fig. 4). Several examined phenotypic characters in the examined lobate species of *Calogaya*, *Gyalolechia* and *Teuvoahtiana* are responsible for this division, for example, the absence of vegetative diaspores, frequent and abundant apothecia, which are mostly sessile and larger up to 1.5 mm in diam. (vs. 0.9 mm in *Wetmoreana* spp.), the amphithecium being dominant over the parathecium, mostly widely ellipsoid ascospores (vs. regularly ellipsoid), and the frequent presence of CaOx crystals in the thalline cortex (vs. in the thalline medulla). However, to confirm the indicated discriminatory phenotypic characters between those two groups of lichens, it is necessary to consider an expanded dataset with more representatives of lobate species from different genera.

**Fig. 6:**
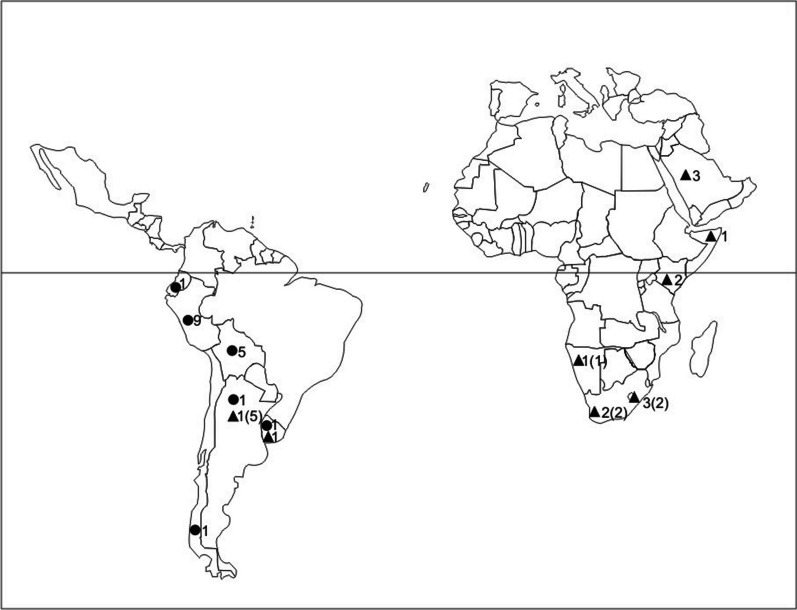
Map of world distribution of *Wetmoreana ochraceofulva* (black triangle) and *W. variegata* (black circle). The numbers in parentheses refer to additional specimens cited in the literature but not examined by us

The newly recognized *Wetmoreana ochraceofulva/ W. variegata* complex in South America, Africa, and the Arabian Peninsula was analyzed by us in terms of molecular and phenotypic variation using PBPB and MRPP. Molecularly, the two species are separated, but with only moderate support and with low genetic divergence (Fig. 3, Additional file 8: Fig. S2). *Wetmoreana ochraceofulva* was described from Somaliland (East Africa) at the end of nineteenth century (Müller 1885). According to Dodge (1971), there are four other taxa with *W. ochraceofulva*-morphology in Africa, namely *Gasparrinia gracilescens*, *G. sympageella*, *G. granulifera*, and *G. granulifera* var. *subvitellina*. Also, Müller indicated in the protologue the similarity of *W. ochraceofulva* with *G. granulifera* (Müller 1885, p. 504). Therefore, more studies are needed on African material of *W. ochraceofulva* to detect potential (semi-)cryptic diversity. Kärnefelt (1988, 1990) synonymized the South American *Callopisma subnitidum* with *W. ochraceofulva* and discussed the disjunctive geographic range of this species. This was also supported by our PBPB analyses, since *Callopisma subnitidum* (syntype, PBPB no. 49) grouped with full support with African *W. ochraceofulva* (Fig. 2, Additional file 12: Table S3). However, the typification of the name *Callopisma subnitidum*, and consequently its placement as a synonym of *C. ochraceofulva*, is to be questioned and this topic is discussed under the species *W. subnitida* below. The distribution of *W. ochraceofulva* on the South American continent appears narrow, while the species is dominant on the African continent (Fig. 6).

The other species similar to *W. ochraceofulva* that is common on the South American continent is the newly proposed *W. variegata*. Our molecular studies indicate that both are closely related, and we suppose that the two taxa have recently diverged, given the short stem branches leading to each (Fig. 3). In comparison to *W. ochraceofulva*, *W. variegata* is a highly variable species which may resemble, besides *W. ochraceofulva*, also other species, such as *W. appressa* and *W. texana*. *Wetmoreana variegata* differs from *W. appressa* and *W. texana* in some morphological aspects (e.g., distinctly thinner ascospore septum compared to *W. appressa*, and distinctly shorter conidia and a limited layer of CaOx crystals in the medulla compared to *W. texana*), and by their phylogenetic positions (Figs. 2–3). Separating *W. variegata* from *W. ochraceofulva* can be problematic when a specimen of the former may produce isidia, as does *W. ochraceofulva*. Still, some other distinguishing features may discriminate between the two taxa (Table 3). In the PBPB analyses, both taxa were placed on the phylogenetic tree separately (Fig. 2, Additional file 7: Fig. S1). Also, the MRPP comparison between the *ochraceofulva*/ *variegata* and *sliwae*/ *subparviloba*/ *brachyloba* complexes shows the largest A value when all taxa are treated separately (GR06, all datasets), or at least *W. ochraceofulva* and *W. variegata* (GR05 and GR04, only complete datasets) (Fig. 5, Additional file 17: Fig. S10). When defining the clades only molecularly, these being less well sampled, all solutions have similar support (Additional file 17: Fig. S10c, d). However, MRPP analyses focusing on species pairwise comparisons show some support for distinguishing the two taxa after accounting for apothecial traits and expanding sampling to include additional binned specimens (Table 2). The PCA ordination of *Wetmoreana* supports the separation of *W. ochraceofulva* and *W. variegata*, but only when apothecial characters are included. *Wetmoreana ochraceofulva* forms one uniform group, whereas *W. variegata*, with its high morphological variation, overlaps with other *Wetmoreana* species on this diagram (Additional file 13: Fig. S6). Overall, the separation of the two species is not supported in the case of an extended dataset including additional genera (Fig. 4).

**Table 3:** Comparison of *Wetmoreana ochraceofulva* and *W. variegata*

Character	*W. ochraceofulva*	*W. variegata*
Apothecium diam. (mm)	0.4–0.6 (n = 2)	0.2–0.9 (n = 7)
Apothecial margin	Slightly prominent to level with disc	Prominent, rarely level with disc
Amphithecium *vs* parathecium	Parathecium dominant	Both ± evenly developed
Cortex of amphithecium	Indistinct	Well developed, thin
Spores (µm) Spore septum (µm)	(10–)12.6 ± 1.5(–16) × (4–)5.7 ± 0.8(–8) (n = 39) (2–)2.9 ± 0.3(–4)	(10–)13.1 ± 1.5(–17) × (5–)6.3 ± 0.75(–9) (n = 125) (3–)3.6 ± 0.5(–5)
Spore length/septum ratio (M_min_–M–M_max_)	4.3–4.4–4.4	3.2–3.6–4.1
Marginal lobes	Plane	Plane to convex
Marginal lobes length (mm)	0.3–2.4 (n = 14), mean length 2.0 ± 0.3 (only max. values)	0.2–4.5 (n = 19), mean length 2.5 ± 0.9 (only max. values)
Marginal lobe thickness (µm)	(50–)215 ± 73(–400) (n = 19)	(125–)296 ± 119(–600) (n = 19)
Thalline areoles	Plane	Plane to convex
Vegetative propagules	Isidia, always present	Schizidia, rarely isidia, or absent
Substrate	Siliceous or calcareous rocks	Mostly siliceous rocks
Distribution	Africa, Arabian Peninsula and South America	South America
References	This paper	This paper

The newly proposed *Wetmoreana rubra* is a highly divergent species, confirmed besides molecular data also by phenotypic characters (Figs. 3–4). The morphology of this species is rather unique within the genus, characterized by a sublobate thallus, ascospores with very thin septa, partly immersed apothecia, and occurrence on strongly calcareous rocks. The PCA ordination diagram shows that *Cinnabaria boliviana* forms a group together with *W. rubra*. Although both species are separated molecularly within *Teloschistoideae*, they are superficially similar and may grow side by side on calcareous rock substrate in the field (Wilk et al. 2021: Fig. 2).

Another new species recognized in this study is *Wetmoreana sliwae*. It has a strongly supported sister group relationship with *W. texana* (Fig. 3), although both species are completely different in morphology, squamulose versus lobate, respectively. Instead, the samples of *W. sliwae* overlap morphologically with the unrelated genus *Squamulea*, rather than with other representatives of *Wetmoreana*, in the PCA ordination diagram (Fig. 4). *Wetmoreana sliwae* is a variable species. Some specimens producing small lobules on the margins of squamules and slightly thinner ascospores are treated here as a separate taxon at the subspecies level, namely *W. sliwae* ssp. *subparviloba*. PCA ordination analyses partially support their separation, depending on the data set analyzed, i.e., regarding only the *W. sliwae* clade or all *Wetmoreana* spp. (Additional files 13 and 16: Figs. S6 and S9). PBPB (only ML) and MRPP analyses support the separation of both infraspecific taxa (Fig. 2, Table 2).

Among the twelve query *Caloplaca* s. lat. species, for which molecular data are currently unavailable, seven were placed within *Wetmoreana* based on ML and MP weighting methods. Those are *Callopisma subnitidum* (syntype), *Caloplaca bahiensis*, *C. brachyloba*, *C. chapadensis*, *C. rubina* var. *evolutior* (syntype), *C. subnitida*, and one unnamed species, *Caloplaca* sp. 1. The query species *C. brachyloba* was placed within *Wetmoreana* in a sister group relationship with *W. sliwae* with full support, based on both weighting techniques in PBPB (Fig. 2, Additional file 7: Fig. S1). The species is quite similar to *W. sliwae* and differs from the latter in having a thinner areolate to subsquamulose thallus, paraplectenchymatous and more-or-less even thalline cortex, ± continuous algal layer, smaller apothecia, orange ostiole of pycnidia, and CaOx crystals within medulla of thallus. The separation of both taxa was supported by all analyses (Table 2, Fig. 4, Additional files 13 and 16: Figs. S6 and S9).

The query species *Caloplaca chapadesis* was located within the *W. rubra* clade with strong support in both ML and MP analyses, suggesting a close affinity with the latter (Fig. 2, Additional file 7: Fig. S1). The obscurely lobate *W. chapadensis* has a rather unique morphology, characterized by the presence of a prothallus and by ascospores with very thick septa, clearly different from *W. rubra*. Moreover, both species grow on different rock substrates, *W. chapadensis* on siliceous and *W. rubra* on calcareous rocks. The PCA diagram clearly shows the uniqueness of *W. chapadesis* within *Wetmoreana* (Additional file 13: Fig. S6), supporting its recognition as a species distinct from *W. rubra*.

The placement of *Caloplaca bahiensis* within *Wetmoreana* is not resolved in the same way by the two analyses used (Fig. 2, Additional file 7: Fig. S1). It is either nested within the *W. brouardii* (ML, both studied samples) or the *W. variegata* clade (MP, only one studied sample; the other one has different unsupported placement). The partially recovered affinity of *W. bahiensis* with *W. brouardii* is considered as more accurate due to morphological characters such as the thickness and color of the thallus and the length of the marginal lobes. Besides *W. brouardii*, *W. bahiensis* grouped with *W. appressa* and *W. subnitida*, taxa of the same clade (Fig. 2). This group of four species (*W. brouardii*, together with *W. appressa*, *W. bahiensis* and *W. subnitida*) is recognized here as the *W. brouardii* complex. *Wetmoreana bahiensis* is a poorly known species, the epithet not having been validly published (without description) by Zahlbruckner (Luetzelburg 1923; Mackenzie-Lamb 1963). The name was detected by us thanks to Clifford Wetmore’s note on one of the specimens of this taxon examined and the epithet is taken up below for this taxon. Based on our study, *W. bahiensis* is superficially similar to the recently described *W. intensa* (Aptroot et al. 2021). Both taxa are reported from Brazil from the same or close to the same region. Interestingly, *W. intensa* is sister to *W. brouardii* in our phylogenetic analysis (Additional file 8: Fig. S2), just as *W. bahiensis* in the PBPB analysis (ML only, Fig. 2). *Wetmoreana bahiensis* and *W. intensa* may perhaps be conspecific, but more studies are needed to compare both taxa, especially in their anatomical structure. Aptroot et al. (2021) reported that in *W. intensa* the thallus is quite thick, with the marginal lobes up to 1 mm long, resembling *Rusavskia elegans*. In contrast, *W. bahiensis* has longer lobes reaching 2 mm in length, the thallus is thin, and the marginal lobes are 100–200 µm thick. Also, the thalline medulla of *W. bahiensis* is filled with crystals of the secondary metabolites, mixed with some smaller clusters of CaOx crystals, all prominent in polarized light. From *W. brouardii*, *W. bahiensis* differs in lacking vegetative propagules, containing abundant crystals in the medulla of the thallus and apothecia, rather common apothecia with coarsely orange pruinose discs and ascospores with thicker septum up to 5 µm (vs. 3.5 µm). *Wetmoreana bahiensis* differs from *W. subnitida* in having coarsely orange pruinose discs of apothecia, slightly narrower ascospores (4–5.5 µm vs. 4–7 µm) with slightly thicker septa (mean thickness 4.6 µm vs. 3.2 µm), and abundant crystals in the whole medulla and the apothecia. It differs from *W. appressa* in having reddish colored thallus, coarsely orange pruinose apothecial discs, distinctly smaller ascospores [(9–)10.8(–13) × (4–)4.6(–5.5) µm vs. (10–)12.6(–15) × (5.5–)6.8(–8) µm], with thinner septa (4–5 µm vs. 4.5–9 µm), and distinctly thinner conidia [(0.5–)0.7(–1.1) µm thick vs. (0.9–)1.1(–1.3) µm].

The placement of *Wetmoreana subnitida* within the *W. brouardii* complex was recovered by both weighting methods in the PBPB with full support (Fig. 2, Additional file 7: Fig. S1). *Wetmoreana subnitida* differs from *W. brouardii* in having abundant apothecia, ascospores with a wider septum, up to 5 µm (vs. 3.5 µm), and a partly white pruinose thallus lacking vegetative propagules. It differs from the closely related *W. appressa* in having a reddish colored thallus, smaller apothecia up to 0.5 mm in diam. (vs. 0.9 mm in diam.), apothecial discs ± concolorous with the thallus, distinctly thinner ascospore septa (2–5 µm vs. 4.5–9 µm), and absence of colorless crystals in the thallus and apothecia.

Unexpectedly, the type of *Wetmoreana appressa*, for which molecular data are unavailable, grouped with the *W. brouardii* complex in the PBPB analysis (only ML, Fig. 2). The separation of *W. appressa* from *W. brouardii* is clear and without a doubt. It differs from *W. brouardii* in having abundant apothecia, wider ascospores with a noticeably thicker septum up to 9 µm (vs. 3.5 µm), and lacking vegetative diaspores and by containing CaOx crystals in the medulla. *Wetmoreana appressa* is also different from *W. bahiensis* and *W. subnitida*, the taxa grouping with *W. brouardii,* by having a distinctly thicker thallus with CaOx crystals (absent in *W. subnitida*), and by larger ascospores with a much wider septum. Our analyses indicate that the sequenced specimen of *W. appressa* (available in GenBank) is not conspecific with the studied type of this taxon. Both examined specimens differ in morphology, and the sequenced *W. ‘appressa’* may belong to an undescribed species. The sequenced *W. ‘appressa’* differs from the type material of *W. appressa* in having a thallus with bullate centre, shorter and broader marginal lobes, and a thinner ascospore septum (see notes under *W. ‘appressa’*). In the PBPB analyses, the sequenced *W. ‘appressa’* has sister position to *W. decipioides* (Fig. 2, Additional file 7: Fig. S1).

Finally, query species *Caloplaca* sp. 1 grouped in *Wetmoreana* and its placement within the genus differs depending on the analyses used. It either is sister to the *W. rubra* clade (ML) or nested within *W. variegata* clade (MP) (Fig. 2, Additional file 7: Fig. S1). The studied specimen was published previously as *Callopisma brachylobum* by Malme (1926). However, the species differs from *W. brachyloba* in having distinctly placodioid thallus, with distinct, yet rather short marginal lobes about 1.5 mm long and central convex areoles (for more details see notes under *W. circumlobata*, the name under which it is formally described below). From *W. rubra*, *W. circumlobata* differs by larger ascospores [(13–)16.2(–19) × (7–)7.5(–9) µm vs. (9–)12(–15) × (5–)5.7(–7) µm], with a distinctly thicker septum [(3–)4.5(–5) µm vs. (1–)1.1(–2) µm], better-developed lobes, and ecology occurring on siliceous rocks. From *W. chapadensis*, *W. circumlobata* differs in having larger marginal lobes (0.3–1.4 × 0.2–1.0 mm vs. 0.2–0.5 × 0.1–0.4 mm), absent of prothallus, larger ascopores [(13–)16.2(–19) × (7–)7.5(–9) µm vs. (11–)13.3(–15) × (6–)6.9(–8) µm]), with a thinner septum [(3–)4.5(–5) µm vs. (5–)6.3(–7) µm], and the presence of CaOx in the thallus. Finally, *W. circumlobata* differs from *W. variegata* in having shorter marginal lobes (0.3–1.4 × 0.2–1.0 mm vs. 0.2–4.5 × 0.1–2.0 mm), convex central areoles, and larger ascospores [(13–)16.2(–19) × (7–)7.5(–9) µm vs. (10–)13.1(–17) × (5–)6.3(–9) µm], and thinner conidia [(3.3–)3.8(–4.3) × (0.7–)0.9(–1.2) µm vs. (1.9–)3.2(–4.2) × (0.8–)1.2(–1.7) µm].

Four query taxa, *Caloplaca muelleri*, *C. rubina* var. *evolutior*, *C.* cf. *tucumanensis*, and *C. xanthobola*, grouped with *Squamulea* in the PBPB (Fig. 2, Additional file 7: Fig. S1). In the case of *C.* cf. *tucumanensis* and *C. xanthobola*, the results only indicate that these species are similar to *Squamulea*, but they likely do not belong to that genus because the species produce a clearly prosoplectenchymatic exciple. An expanded dataset, including members of more *Teloschistaceae* genera, is needed for more correct binning and to elucidate the possible systematic positions of *C.* cf. *tucumanensis* and *C. xanthobola*. In contrast, *C. muelleri* and *C. rubina* var. *evolutior* are very similar to *Squamulea* spp. and both may even constitute synonyms of *S. subsoluta*. However, we prefer to keep them separate and proposed new combinations, i.e., *Squamulea muelleri* and *Squamulea evolutior* (see notes under these taxa below). The latter species differs from *S. subsoluta* in some aspects, e.g., the squamules in *S. evolutior* are strongly reduced in the central part and only marginal incised squamules are present. Although *S. subsoluta* may also have a reduced thallus, the squamules are dispersed and present in the central portion between apothecia. Both species, *S. evolutior* and *S. subsoluta*, are likely present on the Juan Fernández Islands (Chile). *Caloplaca rubina* appears to be a tiny species, producing very small, vivid red apothecia and small, scattered areoles. The superficial morphology of the species (we could not study its anatomy due to the small amount of material, and thus this taxon was not included in our PBPB analyses) agrees with *Squamulea*, and *C. rubina* can be considered to belong to this genus. An anatomical study should be done especially concerning the apothecial exciple, or molecular analysis based on fresh material, to possibly transfer this taxon to *Squamulea*.

One of three specimens of *Teuvoahtiana altoandina*, i.e. specimen PBPB no. 92, grouped with either *Teuvoahtiana* (MP) or *Calogaya* (ML) (Fig. 2, Additional file 7: Fig. S1). The determination of this species needs to be clarified; it may represent an undescribed species of *Calogaya*.

The *Caloplaca fernandeziana* clade represents an undescribed, molecularly clearly separate genus within *Caloplacoideae* (Wilk et al. 2021: Fig. 1). The taxon is quite divergent morphologically from the rest samples studied due to producing typical areolate thallus and biatorine or pseudolecanorine apothecia.

## Taxonomy

***Wetmoreana*** Arup et al., Nord. J. Bot. 31: 66 (2013).

*Synonym: Fulgogasparrea* S.Y. Kondr. et al., in Kondratyuk et al., Acta Bot. Hung. 55: 268 (2013), **syn. nov.**

*Type species: Fulgogasparrea decipioides* (Arup) S.Y. Kondr. et al., in Kondratyuk et al., Acta Bot. Hung. 55: 272 (2013).

*Basionym: Caloplaca decipioides* Arup, in Lumbsch et al., Phytotaxa 18: 29 (2011).

*Type:* **South Korea:** Gangwon Province, Inje-gun, Buk-myun, Yongdae-ri, Sorak-san National Park, 2006, *G. Thor* 20,768 (UPS!—holotype).

*Diagnosis:* Thallus saxicolous, crustose-lobate, or squamulose, marginal lobes short or long, squamule surface undulate and verruculose, vegetative propagules often present (soredia, isidia, schizidia or papillae), prothallus absent (except for *W. chapadensis*), algal layer often irregular or algae in distinct columns, CaOx crystals often located in the thalline medulla, apothecia often scarce and not abundant, mostly erumpent and relatively small up to *ca.* 1.0 in diam., parathecium (proper exciple) prospolectenchymatic (except for *W. bahiensis*), ascospore length and septa varied, spores regularly ellipsoid, pycnidia mostly present, often distinct, conidia ovoid or bacilliform.

*Distribution and ecology:* The species grow mainly on siliceous rocks, rarely calcareous (*W. rubra*), or on both types of rocks (*W. ochraceofulva* and *W. variegata*). They are reported from the Americas, Africa, and Asia, where they were collected in exposed or shaded habitats, at different elevations, from 20 to 4500 m. Among the species, *W. sliwae* occurred at the highest-altitude sites, i.e., 3500–4500 m above sea level. *Wetmoreana appressa* s. lat., *W. awasthii,* and *W. decipioides* occurred at the lowest elevations up to 1000 m (Wetmore and Kärnefelt 1998; Joshi and Upreti 2007; Lumbsch et al. 2011). In contrast, *W. brouardii* occurred in the widest ranges of altitude between 10 to 2800 m above sea level (Wilk 2021).

*Notes:* The genus *Wetmoreana* is similar to other lobate or squamulose members of different genera such as, e.g., *Calogaya*, *Cinnabaria*, *Squamulea*, *Teuvoahtiana*, or *Gyalolechia*.

The squamulose members of *Wetmoreana* are very similar to *Squamulea* ssp. The latter genus, however, differs from *W. brachyloba*, *W. sliwae*, and *W. sliwae* ssp. *subparviloba* in having a paraplectenchymatous apothecial exciple, mostly smooth/intact surface of the squamules, thallus sometimes slightly lobate at margin (*S. parviloba*), frequent occurrence of prothallus (e.g., *S. evolutior*, *S. muelleri* and *S. subsoluta*), mostly continuous algal layer in the thallus, mostly smaller apothecia (usually up to 0.9 mm in diam.), a less distinguishable thalline margin of apothecia, smaller ascospores [spore length 8–15 µm vs. (9)12–22 µm; based on examined specimens], clearly widely ellipsoid ascospores (vs. usually regularly ellipsoid), indistinct pycnidia, and commonly absent of CaOx crystals in the thallus and apothecia.

The lobate members of *Wetmoreana* differ from the studied lobate species of *Calogaya*, *Gyalolechia,* and *Teuvoahtiana* by the frequent occurrence of the vegetative diaspores, often rare and not so abundant apothecia, which are mostly erumpent and clearly smaller up to 0.9 in diameter (vs. 1.5 mm), ellipsoid ascospores (vs. often broadly ellipsoid), frequent occurrence of CaOx crystals and the location of these in the thalline medulla (vs. in the thalline cortex when present).

One of the sublobate species of *Wetmoreana*, *W. rubra*, is very similar to the monospecific genus *Cinnabaria*. *Cinnabaria boliviana* differs from *W. rubra* in having a more yellowish color of the thallus, arrangement of algae in distinct groups, persistently immersed apothecia, widely ellipsoid ascospores (vs. regularly ellipsoid), thicker ascospore septa (2.0‒3.0 µm vs. 1.0‒2.0 µm), lower ascospore length/septum thickness ratio (4.5‒5 vs. 9.5‒13.5 times), indistinct pycnidia, and ovoid conidia (vs. short to long bacilliform).

Among the *Wetmoreana* spp. only *W. rubra* has ascospores with surprisingly very thin septa, i.e., from 1 to 2 µm wide. A further three species in the genus i.e., *W. brouardii*, *W. ochraceofulva*, and *W. sliwae* ssp. *subparviloba* also have thin spore septa, but in a slightly wider range and from 2 to 4 µm. Such thin ascospore septa occur throughout *Teloschistaceae* mainly in *Gyalolechia*, *Calogaya*, *Cerothallia*, *Stellarangia* and *Xanthocarpia*. In contrast, *Wetmoreana* spp. with noticeably thick spore septa include *W. appressa* (4.5–9.0 µm), *W.* ‘*appressa*’ (5.0–7.5 µm), *W. brachyloba* (3.0–7.0 µm), and *W. chapadensis* (5.0–7.0 µm). For ascospore length, *W. sliwae* has larger ones (12‒22 µm), while *W. bahiensis* and *W. subnitida* are much smaller (from 8 to 13 µm). As shown here, ascospores are very diverse in this group of lichens. Indeed, the morphometric traits which characterized ascospores (five in number) received low weights in both PBPB analyses (ML and MP), except for ascospores L/W ratio (both analyses) and ascospores length (ML only) (Additional file 2: Table S2). And they cannot be used as diagnostic traits at the genus-level.

In contrast, the occurrence of CaOx crystals in the thallus and apothecia is a good discriminating character for this group of lichens. The traits characterizing CaOx crystals (six in number) received significant weighting scores in both or at least one PBPB analysis. 60% of the examined *Wetmoreana* spp. have such crystals in the thallus medulla, sometimes also in apothecia, and very rarely in the thalline cortex (only in *W. rubra* and in small amounts). In some species, the crystals form a relatively thin and distinct crystalline layer that separates the algal layer from the lower part of the crystal-free medulla. Such a limited crystalline layer occurs in *W. appressa*, *W. circumlobata*, *W. ochraceofulva*, and *W. variegata*. Taxa in which CaOx crystals have not been observed are *W. brouardii*, *W. chapadensis*, *W. decipioides*, *W. sliwae*, *W. sliwae* ssp. *subparviloba*, and *W. subnitida*. CaOx crystals were also found in some other studied species unrelated to *Wetmoreana*, such as *Calogaya pusilla*, *C. biatorina*, *Gyalolechia gomerana*, *Teuvoahtiana altoandina*, or closely related *Cinnabaria boliviana*. Compared to *Wetmoreana*, the crystals are not always present in those taxa, and if they are present, they often occur in the thalline cortex. CaOx crystals were used as a diagnostic feature by Wetmore and Kärnefelt (1998) in the case of *W. appressa* and *W. texana*. The authors studied nineteen species of “Gasparrinia”, and in three of them [including also *Caloplaca eugyra*], the crystals were present in the thalline medulla. Other authors also examined the occurrence of CaOx crystals within *Teloschistaceae*. For example, within the *Caloplaca saxicola* group, the crystals are commonly present in the thalline cortex, but only in one species, *Calogaya pseudofulgensia*, they occur abundantly also in the medulla of the thallus (Gaya 2009). In the case of *Variospora aurantia* and *V. flavescens*, the presence of CaOx crystals in the thalline cortex is treated as the most helpful feature to distinguish the two similar species (e.g., Śliwa and Wilk 2008). The occurrence of the CaOx crystals in the thalline cortex is manifested as pruina on the thallus surface, which protects lichens against excessive insolation and pollution (Modenesi 1993; Modenesi et al. 1998). While data on CaOx crystals found in the inner parts of the thallus, such as the medulla, are rather sparse, and their role is unknown.

### Key to *Wetmoreana* species

1. Thallus lobate or sublobate** 2**
  Thallus squamulose** 13**
2. Thallus with distinct marginal lobes more than 1.5 mm long** 3**
  Thallus with short marginal lobes up to 1.5 mm long or sublobate** 10**
3. Thallus thick, lobe thickness up to 600 µm, thallus orange** 4**
  Thallus thin, lobe thickness up to 200 µm, thallus red tinged** 8**
4. Apothecia always present, ascospores with very thick septa, 4.5–9.0 µm wide, vegetative propagules absent **W. appressa**
  Apothecia may present, ascospores with septa up to 5 µm wide, vegetative propagules usually present** 5**
5. Apothecia unknown, soralia always present, thalline algal layer continuous, absence of any crystals prominent in polarized light in the thallus, found in Asia **W. decipioides**
  Apothecia rare, schizidia or isidia present, when absent numerous apothecia present, thalline algae usually in distinct groups, CaOx crystals prominent in polarized light usually present in the thallus** 6**
6. Schizidia or isidia present, conidia ovoid to short bacilliform, from 2 to 4.5 µm long, lobes up to 4.5 mm long, CaOx crystals always present as a limited crystalline layer between algal layer and medulla in the thallus** 7**
  Schizidia always present, conidia long bacilliform, from 3 to 7 µm long, lobes up to 2(–2.4) mm long, CaOx crystals may be present, but then fill the entire medulla in the thallus **W. texana**
7. Schizidia present or rarely isidia, when there are no vegetative propagules, abundant apothecia present, lobes up to 4.5 mm and up to 600 µm thick, on siliceous rocks, found in South America **W. variegata**
  Isidia always present, apothecia very rare, and if present, co-occur with isidia, lobes up to 2.4 mm and up to 400 µm thick, ascospores and their septa slightly smaller, on siliceous or calcareous rocks, found in Africa and the Arabian Peninsula, rarely in South America **W. ochraceofulva**
8. Thallus with laminal papillae, apothecia rare, ascospore septa up to 3.5 µm wide, pycnidia rare **W. brouardii**
  Vegetative propagules absent, apothecia usually present and abundant, ascospore septa up to 5 µm wide, pycnidia always present** 9**
9. Apothecial discs not or only slightly whitish pruinose, ascospores up to 7 µm wide, marginal lobes up to 1 mm wide, medullary crystals prominent in polarized light absent **W. subnitida**
  Apothecial discs coarsely orange pruinose, ascospores up to 5.5 µm wide, marginal lobes up to 0.6 mm wide, medullary crystals prominent in polarized light present **W. bahiensis**
  (similar to recently published** W. intensa** but the latter differs in having lobes up to 1 mm long, unknown apothecia and pycnidia, and thalline surface with dark punctiform depressions [Aptroot et al. 2021]; species may be co-specific and require further study)
10. Thallus sublobate, sublobes up to 0.5 mm, prothallus orange, ascospore septa up to 7 µm **W. chapadensis**
  Thallus lobate, lobes more than 0.5 mm, prothallus absent, ascospore septa up to 5 µm or less** 11**
11. Thallus with soredia/blastidia, apothecia and pycnidia unknown, found in Asia (India) **W. awasthii**
  Vegetative propagules absent, apothecia common, pycnidia abundant and distinct, found in South America** 12**
12. Thallus with reddish tinge, apothecia distinctly erumpent, ascospore septa very thin up to 2 µm, CaOx crystals fill the entire medulla in the thallus, on strong calcareous rocks **W. rubra**
  Thallus orange, apothecia sessile, ascospore septa up to 5 µm, CaOx crystals present as a limited crystalline layer between algal layer and medulla in the thallus, on siliceous rocks **W circumlobata**
13. Thallus stipitate squamulose, thick, up to 850 µm, algae in distinct groups in the thallus, absence of any crystals prominent in polarized light in the thallus and apothecia, high mountain taxa** 14**
  Thallus areolate to squamulose, thinner, up to 200 µm, algal layer ± continuous, medullary CaOx crystals always present **W. brachyloba**
14. Squamule surface slightly undulate and matte, squamule margins slightly crenate, apothecia common, ascospores large, 12‒22 × 5‒9 µm, septa 2‒6 µm **W. sliwae subsp. sliwae**
  Squamule surface strongly convex and shiny, squamule margins lobulate, apothecia uncommon, ascospores distinctly smaller, 9‒13 × 5‒7 µm, septa 2‒3 µm **W. sliwae subsp. subparviloba**

#### ***Wetmoreana bahiensis*** Wilk & Lücking, sp. nov. (Fig. 7a–g)

**Fig. 7:**
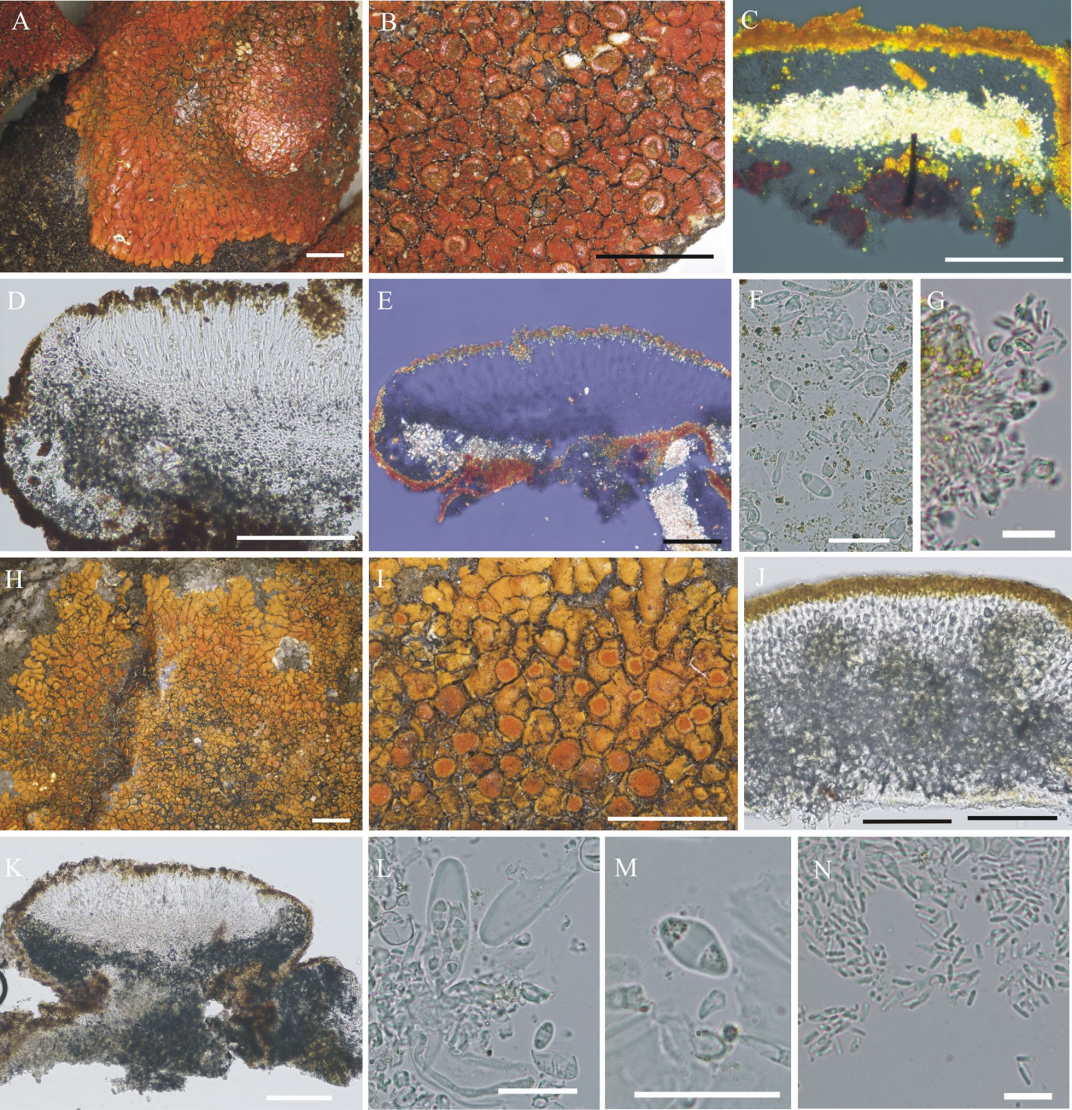
Species of the *Wetmoreana brouardii* clade. **A**–**G** *Wetmoreana bahiensis* (lectotype, **A** thallus habit, **B** apothecia with coarse orange pruinose discs, **C** section of thallus in polarized light showing medulla filled with crystals of unknown origin and some calcium oxalate, **D** section of apothecium showing paraplectenchymateous parathecium, **E** section of apothecium showing crystals prominent in polarized light, **F** ascospores, **G** conidia). **H**–**M** *W. subnitida* (lectotype, **H** thallus habit, **I** erumpent to sessile apothecia, **J** section of thallus showing paraplectenchymatous upper cortex, **K** section of apothecium showing prosoplectenchymatous parathecium, **L** ascus with spores, **M** ascospore, **N** conidia). Scale in **A**, **B**, **H**, **I** = 2 mm, in **C**, **D**, **E**, **J**, **K** = 100 µm, in **F**, **L**, **M** = 20 µm, in **G**, **N** = 10 µm

*MycoBank:* MB851723.

*Etymology:* The epithet is derived from the name of the area where the species was collected, an epithet previously proposed by Zahlbruckner but not validly published (Luetzelburg 1923).

*Diagnosis:* Similar to *Wetmoreana brouardii* but vegetative propagules not present, apothecia common, ascospore septum thicker, 4‒5 µm versus 2‒3.5 µm and thalline medulla filled with crystals prominent in polarized light.

*Type:* **Brazil:** Serra Chuquê, Staat Bahia, *s.d.*, *P. von Luetzelburg 407* (M 0207257—holotype).

*Synonym: Caloplaca bahiensis* Zahlbr. ex Luetzelb., Estud. Botan. do Nordeste (Inspec. Federal. Obras contra Seccas, Rio de Janeiro, Publ. no. 57) 3: 232 (1923), nom. inval.

*Description*: Thallus crustose, areolate in centre, lobate at margin, orbicular to irregular in outline, tightly attached to substratum, red to reddish orange, K + purple, epruinose, pseudocyphellae or other cracks absent, areoles polygonal to granular, plane to convex, marginal lobes plane, widened at tips, 0.4‒1.8 mm long, 0.2‒0.6 mm wide at the tips, 95–230 µm thick; vegetative propagules absent; prothallus absent; upper cortex well developed, distinct, 15‒70 µm thick, paraplectenchymatous, necral layer absent, colorless crystals absent; algal layer continuous; medulla always filled with colorless crystals of unknown origin, luminescent in polarized light, some CaOx crystals may also present. Apothecia few to abundant, ± scattered, round, sessile, zeorine, 0.3‒0.6(1.2) mm in diam.; disc plane also in old apothecia, K + purple, coarsely orange pruinose, entire (without cracks); both margins not or almost not distinguishable from each other, apothecial margin prominent, thick, epruinose, thalline margin ± persistent to partly reduced, even; parathecium 60‒85 µm thick, paraplectenchymatous; amphithecium dominant, 102–119 µm thick, algal layer discontinuous in the central part, with abundant colorless crystals (pol + white), apothecial cortex 10‒17 µm thick, paraplectenchymatous, colorless crystals absent; epihymenium brownish tinged, K + purple; hymenium 85‒94 µm thick; hypothecium 120 µm thick, prosoplectenchymatous, hyaline, oil droplets absent; paraphyses simple, with upper cell 3‒5 µm wide; asci 8-spored; ascospores hyaline, regularly ellipsoid, thin-walled, (9‒)10.8 ± 1.0(‒13) × (4‒)4.6 ± 0.5(‒5.5) µm, length/width ratio 2.3, ascospore septa (4‒)4.6 ± 0.5(‒5) µm, length/septum width ratio 2.4 (n = 13; N = 1). Pycnidia abundant, ostiole red, raised, indistinct, conidia long bacilliform, (2.6‒)3.5 ± 0.5(‒4.3) (n = 38) × (0.5‒)0.7 ± 0.1(‒1.1) (n = 33) µm, length/width ratio (4.5–)4.6 ± 0.1(‒4.7) (N = 2).

*Distribution and ecology:* The species is known only from Brazil, where it was collected on siliceous rock. Unfortunately, information about ecology is unavailable. The regions of Bahia and Parahyba, where the species has been recorded, are generally covered with dry forests, such as caatinga (Cardoso and Queiroz 2008).

*Notes:* In our PBPB analysis, the two samples named *W. bahiensis* grouped in *W. brouardii* (ML, two samples) or in *W. variegata* clades (MP, one sample) depending on the analyses used (Fig. 2, Additional file 7: Fig. S1). Due to the morphological similarity to *W. brouardii*, *W. subnitida* and the closely related *W. intensa*, as well as the PBPB results, we believe that placing *W. bahiensis* within the *W. brouardii* complex is appropriate. For comparison of *W. bahiensis* with the members of the *W. brouardii* complex, see the section Discussion. *Wetmoreana bahiensis* differs from *W. variegata* by having distinctly thinner (95–100 µm vs. 125–600 µm thick in *W. variegata*) and narrower (0.2–0.6 mm vs. 0.1–2.0 mm) marginal lobes, continuous algal layer in the thallus, coarsely orange pruinose apothecia, distinctly smaller ascospores (9–13 × 4–5.5 µm vs. 10–16 × 5–9 µm), lower length/septum width ratio (2.4 vs. 3.2–4.1 times), raised pycnidia, long bacilliform conidia (vs. ovoid to short bacilliform), and abundant crystals of unknown origin, in addition to small portions of CaOx crystals, filled thalline medulla (vs. limited layer of only CaOx crystals at the base of the algal layer).

*Additional specimen examined:* **Brazil:** Serra da Baixa Verde, Staat Parahyba do Norte, March 1920, *P. von Luetzelburg 273* (M 0207256).

#### ***Wetmoreana circumlobata*** Wilk & Lücking, sp. nov. (Fig. 8a–g)

**Fig. 8:**
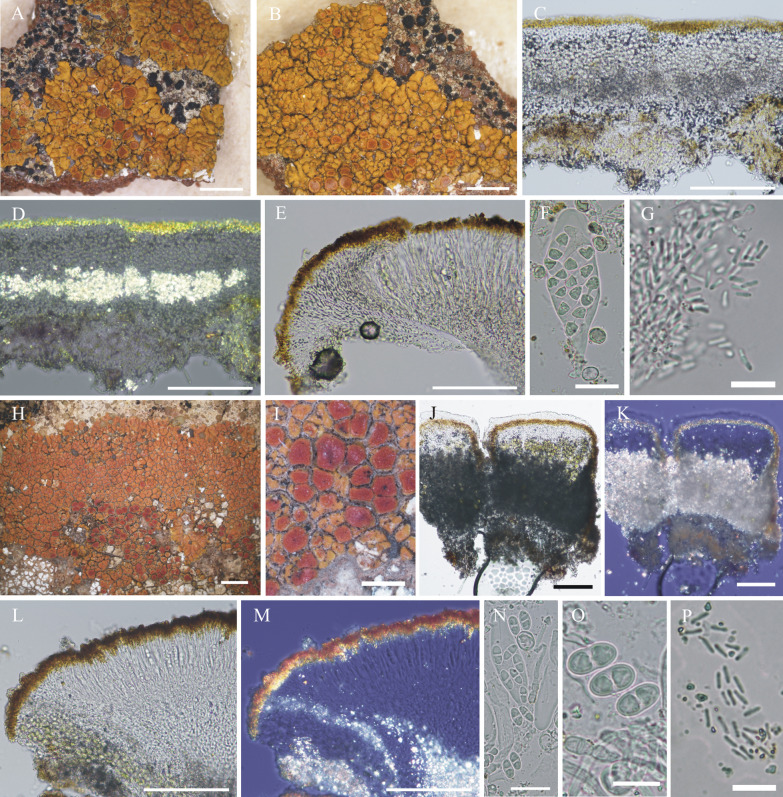
A–G *Wetmoreana circumlobata* (holotype, **A** young placoidioid thallus, **B** mature thallus bullate in the centre, **C, D** sections of thallus showing calcium oxalate crystals below algal layer prominent in polarized light (**D**), **E** section of apothecium showing distinctly prosoplectenchymatous parathecium, **F** ascus with spores, **G** conidia). **H**–**P** *W. rubra* [H sublobate thallus, I erumpent apothecia (holotype), **J**–**K** section of thallus showing thick necral layer on upper cortex and medulla filled with CaOx crystals prominent in polarized light (*K. Wilk 3294*, KRAM), **L** section of apothecium showing prosoplectenchymatous parathecium, **M** section of apothecium showing CaOx crystals in cortex and parathecium (holotype), **N** ascus with spores, O ascospores constricted in the middle (*K. Wilk 3294*, KRAM), P conidia (holotype)]. Scale in **A, B**, H = 2 mm, in I = 1 mm, in **C, D, E, J, K, L, M** = 100 µm, in **F, N** = 20 µm, in **G, O, P** = 10 µm

*MycoBank:* MB851727.

*Etymology:* The epithet is derived from clearly placodioid thallus in this species.

*Diagnosis:* Similar to *Wetmoreana appressa* but central areoles strongly convex, marginal lobes shorter, 0.3–1.4 mm versus 0.4–2.2 mm, ascospores different, 13–19 × 7–9 µm versus 10–15 × 5.5–8 µm, and ascospore septa thinner, 3–5 µm versus 4.5–9 µm.

*Type:* **Paraguay**: Prov. Asuncion (meridion. versus), in pascuo aperto, 21 July 1893, *G. O. Malme 1423* (S F90573—holotype).

*Description:* Thallus crustose, areolate in centre, lobate at margin, orbicular in outline, tightly attached to substratum, orange, K + purple, epruinose, pseudocyphellae or other cracks absent, areoles polygonal, strongly convex to granular, marginal lobes plane, widened at tips, 0.3‒1.4 mm long, 0.2‒1.0 mm wide at the tips, 160‒200 µm thick; vegetative propagules absent; prothallus absent; upper cortex 15‒50 µm thick, paraplectenchymatous, uneven, some cortex cones present, necral layer absent, colorless crystals absent; algal layer continuous to discontinuous; medulla with CaOx crystals forming a limited layer 35–43 µm wide at the base of algal layer. Apothecia abundant, sattered to crowded, round to angular, sessile, zeorine, 0.3‒0.8 mm in diam.; disc plane to slightly convex in old apothecia, darker than thallus, K + purple, epruinose, entire (without cracks); both margins distinguishable but inconspicuous, proper margin prominent, paler than disc, epruinose; thalline margin partly to much reduced, even; parathecium dominate, 100‒190 µm thick, prosoplectenchymatous; amphithecium 85‒95 µm thick, algae abundant, forming continuous layer, apothecial cortex indistinct, colorless crystals absent; epihymenium yellow orange, K + purple; hymenium 100‒110 µm thick; hypothecium 140 µm thick, prosoplectenchymatous, hyaline, oil droplets absent; paraphyses simple, 1 µm broad at base, with upper cell 2.5‒3 µm wide; asci 8-spored; ascospores hyaline, regularly ellipsoid, thin-walled, (13‒)16.2 ± 1.4(‒19) × (7‒)7.5 ± 0.6(‒9.0) µm, length/width ratio 2.2, ascospore septa (3‒)4.5 ± 0.7(‒5) µm, length/septum width ratio 3.5 (n = 22; N = 1). Pycnidia abundant, ostiole dark orange, partly immersed, distinct, conidia long bacilliform, (3.3‒)3.8 ± 0.3(‒4.3) (n = 22) × (0.7‒)0.9 ± 0.1(‒1.2) (n = 18) µm, length/width ratio 3.8 (N = 1).

*Distribution and ecology:* The species was collected in Paraguay, in the open pasture. It grows on siliceous sandstone rocks.

*Notes:* The examined specimen of *W. circumlobata* was previously reported as *Callopisma brachylobum* by Malme (1926) (see the commentary under this species). *Wetmoreana circumlobata* differs from *W. brachyloba* mainly in having a placodioid thallus (vs. areolate-squamulose in *W. brachyloba*), convex central areoles, polygonal to granular (vs. plane to slightly convex, polygonal to sublobate mostly on margins), and apothecia sessile (vs. erumpent). The ascospores and conidia are the same in both species, as is the ecology. Both species were collected in Paraguay on similar loose sandstone rocks. There is more material of *Callopisma brachylobum* collected by Malme in Paraguay (e.g., *G. O. Malme 1355*, *1596*, *1468b*, all in S) and Brazil (*G. O. Malme 1134*, S) (Malme 1926), which needs to be studied to determine its correct taxonomic identity.

*Wetmoreana circumlobata* may be confused with *W. appressa*, *W. ‘appressa’* and *W. variegata*. The new species differs from *W. appressa* in having strongly convex central areoles, shorter marginal lobes (0.3–1.4 mm vs. 0.4–2.2 mm), persistently prominent apothecial margins, partly to much reduced thalline margin, distinctly thicker parathecium (190 µm vs. 70 µm), different ascospores (13–19 × 7–9 µm vs. 10–15 × 5.5–8 µm) with distinctly thinner septa (3–5 µm vs. 4.5–9 µm), and larger conidia (mean length 3.8 µm vs. 3.5 µm). *Wetmoreana circumlobata* is somewhat similar to *W. ‘appressa’* due to the smooth thallus and strongly convex central areoles, but it differs from the latter in having more tightly appressed thallus to the substrate, partly to much reduced apothecial margin (vs. mostly ± persistent), different ascospores (13–19 × 7–9 µm vs. 12–15.5 × 5.5–7 µm) with thinner septa (3–5 µm vs. 5–7.5 µm), and slightly larger conidia (mean length 3.8 µm vs. 3.5 µm). For comparison with *W. variegata* see section Discussion.

#### ***Wetmoreana rubra*** Wilk & Lücking, sp. nov. (Fig. 8h–p)

*MycoBank:* MB851729.

*Etymology:* The epithet is derived from the reddish color of the thallus.

*Diagnosis:* Smilar to *Cinnabaria boliviana* but thallus reddish orange, apothecia only initially immersed, ascospores often narrower in the medium part, ascospore septa thinner, 1–2 µm versus 2–3 µm, and conidia longer, (3.2‒)4.0 ± 0.4(‒5.4) versus (2.3–)3.2 ± 0.5(–4.8).

*Type:* **Bolivia:** Dept. Cochabamba, Prov. Quillacollo, East Cordillera, area of Inkarraya-Sipesipe, dry Inter-Andean Valleys, rocky and shrubby slope, sunny place, E exposition, alt. 3146 m, 17°29′25"S, 66°22′09"W, 17 Dec. 2004, *K. Wilk 3236a* (KRAM L 71767—holotype, LPB, B—isotypes).

*Description:* Thallus crustose, areolate in centre, minutely lobate at margin, orbicular to irregular in outline, 1–6 cm wide, tightly attached to substrate, reddish orange, K + purple, epruinose, pseudocyphellae or other cracks absent, usually parasitized by fungi, areoles 0.2‒0.5 mm wide, polygonal, somewhat convex, marginal lobes convex, widened at tips, 0.3‒1.8 mm long, 0.2‒0.8 mm wide at the tips, 175‒450 µm thick; vegetative propagules absent; prothallus absent; upper cortex 30‒75 µm thick, prosoplectenchymatous, necral layer distinct and thick, always present, 3.5‒35.0 µm thick, some CaOx crystals may present; algal layer ± continuous (some cortex cones present); medulla always filled with CaOx crystals. Apothecia scarce to abundant, crowded, mostly angular, initially immersed, then erumpent, zeorine, 0.2‒0.9 mm in diam.; disc concave, then plane to slightly convex in old apothecia, orange red, K + purple, ± contrasting against thallus, epruinose, distinctly cracked; both margins distinguishable and conspicuous, slightly prominent to level with discs, proper margin thin, ± concolorous with disc, epruinose, thalline margin partly to much reduced, even; parathecium 50‒120 µm thick, prosoplectenchymatous, always with CaOx crystals distributed along the parathecium from base of apothecium; amphithecium 50–120 µm thick, algae abundant, algal layer discontinuous in the central part, apothecial cortex 15‒40 µm thick, always filled with CaOx crystals; epihymenium reddish yellow, K + purple; hymenium 70‒95 µm thick; hypothecium 95‒180 µm thick, prosoplectenchymatous, hyaline, oil droplets absent; paraphyses simple to forked, 1.5–2 µm broad at base, with upper cell 2‒5 µm wide, oil droplets absent; asci 8-spored; ascospores hyaline, ± regularly ellipsoid, some of them ± constricted in the middle, thin-walled, (9‒)12.0 ± 1.5(‒15) × (5‒)5.7 ± 0.5(‒7.0) µm, length/width ratio (1.8–)2.1 ± 0.2(–2.4), ascospore septa (1‒)1.1 ± 0.2(‒2) µm, length/septum width ratio (9.6–)11.3 ± 1.6(–13.6) (n = 55; N = 5). Pycnidia abundant, ostiole red, ± immersed, distinct, conidia long bacilliform, (3.2‒)4.0 ± 0.4(‒5.4) (n = 48) × (0.5‒)1.0 ± 0.2(‒1.3) (n = 38) µm, length/width ratio (3.4–)4.0 ± 0.5(–4.9) (N = 5).

*Distribution and ecology: Wetmoreana rubra* is known only from Bolivia from dry Inter-Andean Valleys, at an altitude of ca. 3000 m. It grows on calcareous rocks, in well-lit conditions together with *Cinnabaria boliviana*.

*Notes:* *Wetmoreana rubra* is one of the most distinctive species in the genus, both molecularly and morphologically (Figs. 3–4). It produces a sublobate thallus and ascospores with very thin septa, slightly constricted at the central part, and occurs on strongly calcareous rocks. Morphologically, it is most similar to the distantly related, but still within the *Teloschistoideae*, *Cinnabaria boliviana*. Both species grow side by side in the field, and the most apparent superficial difference between them is the color of the thallus, which is yellowish orange in *C. boliviana*. Moreover, the apothecia in *C. boliviana* are ± persistently immersed and produce widely ellipsoid ascospores with slightly thicker septa (2–3 µm vs. 1–2 µm wide in *W. rubra*), and distinctly lower length/septum width ratio (4.6 vs. 10.9 times). *Cinnabaria boliviana* also produces different, shorter conidia, ovoid to short bacilliform in shape (length/width ratio 2.6 vs. 4 times), and has a discontinuous algal layer with algae arranged in the distinct groups. While the anatomical structure of the apothecia are very similar in both species, and the apothecia contain CaOx crystals that are characteristically located along the parathecium (but crystals are not always present in *C. boliviana*).

*Wetmoreana rubra* shares some similarities with *Gyalolechia gomerana*. The latter differs from *W. rubra*, however, by having longer and broader marginal lobes [0.5–2.0 × 0.1–1.0 mm vs. 0.3–1.0(–1.8) × 0.2–0.8 mm in *W. rubra*], yellow orange color of the thallus, conspicuous pseudocyphellae on the thallus surface, algae in distinct groups or arranged irregularly, lack of necral layer, sessile and larger apothecia (0.3–1.5 mm vs. 0.2–0.9 mm in diam.), smaller ascospores (8–12 × 4–6 µm vs. 9–15 × 5–7 µm) with thicker septa (2–4 µm vs. 1–2 µm), and different, biguttulate conidia. Moreover, both taxa differ in ecology since *G. gomerana* grows on siliceous rocks. CaOx crystals are usually absent in the thalli in *G. gomerana*; however, crystals of secondary metabolites (insoluble in K and N, pol +) are present. *Wetmoreana rubra* may be confused with *Harusavskia elenkinianoides*, which is also known from South America (Kondratyuk et al. 2017). *Wetmoreana rubra* differs from that species in having smaller marginal lobes [0.3–1.0(–1.8) × 0.2–0.8 mm vs. 2–3.5 × 0.4–1.2 mm in *H. elenkinianoides*], an absence of pseudocyphellae of the thallus surface, smaller apothecia (0.2–0.9 mm vs. 0.4–1.5 mm in diam.), different ascospores (1-septate without a halo vs. simple to 1-septate with a halo), different ecology, and different phylogenetic position (Wilk et al. 2021: Fig. 2).

*Additional material examined:* **Bolivia:** Dept. Cochabamba, Prov. Quillacollo, East Cordillera, area of Inkarraya-Sipesipe, dry Inter-Andean Valleys, rocky and shrubby slope, sunny place: E exposition, alt. 3146 m, 17°29′25"S, 66°22′09"W, 17 Dec. 2004, *K. Wilk 3221b, 3223, 3278* (KRAM, LPB, B); NE exposition, alt. 2846 m, 17°28′39"S, 66°21′43"W, 17 Dec. 2004, *K. Wilk 3294* (LPB).

#### ***Wetmoreana sliwae*** Wilk & Lücking, sp. nov. (Fig. 9a–h)

**Fig. 9:**
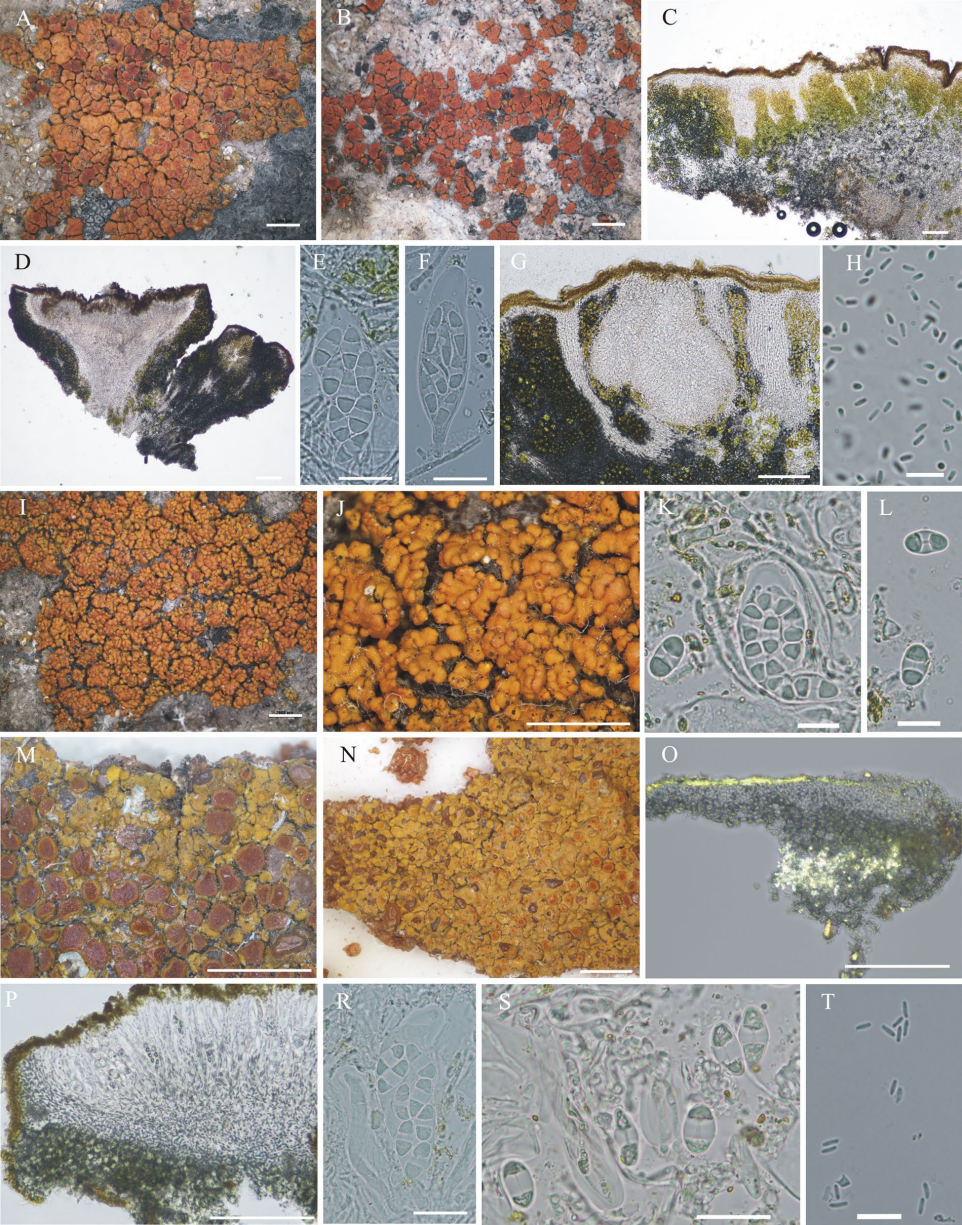
Squamulose members of *Wetmoreana*. **A**–**H** *Wetmoreana sliwae* [A squamulose stipitate thallus (holotype), **B** specimen with reduced thallus (*A. Flakus 16,503*, KRAM), **C** section of thallus showing columnar arrangement of the algal cells (*A. Flakus 16,719*, KRAM), **D** section of apothecium (*A. Flakus 9266*, KRAM), **E**–**F** asci with spores which are variable from narrowly to widely ellipsoid (*A. Flakus 9279*, KRAM), **G** section of pycnidium, **H** conidia (*A. Flakus 9266*, KRAM)]. **I**–**L** *W. sliwae* ssp. *subparviloba* (holotype, A thallus habit, **J** thalline squamules lobulated at margins, **K** asci with spores, **L** ascospors. **M**–**T** *W. brachyloba* [M thallus habit (lectotype), **N** well-developed thallus, **O** section of thallus in polarized light showing medulla filled with CaOx crystals (*B. Balansa*, M 0207259), **P** section of apothecium showing distinctly prosoplectenchymatous parathecium (lectotype), **R** ascus with spores (*B. Balansa*, G 00293574), **S** ascospores (lectotype), **T** conidia (*B. Balansa*, M 0207259)]. Scale in **A**, **B**, **I**, **J**, **M**, **N** = 2 mm, in **C**, **D**, **G**, **O**, **P** = 100 µm, in **E**, **F**, **R**, **S** = 20 µm, in **H**, **K**, **L**, **T** = 10 µm

*MycoBank:* MB851730.

*Etymology:* Named after Lucyna Śliwa, Polish lichenologist, in appreciation of her lichen taxonomic knowledge and her influence on the first author taxonomic experience.

*Diagnosis:* Similar to *Wetmoreana brachyloba* but thallus thicker, distinctly stipitate squamulose with larger apothecia (0.2–1.2 mm vs. 0.2–0.8 mm in diameter), thalline algae clustered in distinct groups, and the thallus and apothecia lack crystals prominent in polarized light.

*Type:* **Peru:** Dept. Arequipa, Prov. Caylloma, Valle del Colca valley, near Achoma village, open semi-desert montane area, alt. 3500 m, 15°39′26"S, 71°43′38"W, 2 July 2006, *A. Flakus 9279 & B. Cykowska* (KRAM L 71769—holotype, LPB—isotype).

*Description:* Thallus squamulose, irregular in outline, loosely attached to the substrate, orange to reddish orange, K + purple, epruinose, matte, small cracks often present, thallus usually parasitized by microscopic fungi, squamules stipitate, surface verruculose, margins crenate, 200‒850 µm thick; vegetative propagules absent; prothallus absent; upper cortex 15‒85 µm thick, prosoplectenchymatous, uneven with distinct cortex cones, necral layer always present, discontinuous, 7‒35 µm thick, colorless crystals absent; algal layer discontinuous and algae in distinct groups or columns or irregularly arranged; medulla without colorless crystals. Apothecia usually present, usually abundant, crowded, rounded to angular, flexuous and undulate when compressed, erumpent, zeorine, 0.2‒1.2 mm in diam.; disc first concave, then plane to slightly convex in old apothecia, dark orange to red, K + purple, darker than thallus or ± concolorous with thallus, epruinose, entire to cracked; both margins distinguishable and conspicuous or inconspicious, slightly prominent to level with discs, proper margin thick and prominent in young apothecia, then level with disc, paler than disc (young) to ± concolorous with disc in mature and old apothecia, thalline margin partly to much reduced, concolorous with thallus, even; parathecium 70‒120 µm, prosoplectenchymatous, consisting of radiating hyphae; amphithecium 85‒170 µm thick, algae abundant, algal layer continuous to discountinuous in the central part, apothecial cortex indistinct, 14‒45 µm thick, consists of small isodiametric cells, colorless crystals absent; epihymenium orange to orange red, K + purple; hymenium 70‒130 µm thick; hypothecium 70‒150 µm thick, prosoplectenchymatous, hyaline, oil droplets often present, abundant; paraphyses simple or slightly branched, 1‒2 µm broad at base, with upper cell not or wider, up to 5 µm, oil droplets absent; asci 8-spored; ascospores hyaline, ± regularly ellipsoid, thin-walled, (12‒)15.8 ± 2.2(‒22) × (5‒)7.1 ± 0.9(‒9) µm, length/width ratio (1.7–)2.2 ± 0.3(‒2.8), ascospore septa (2‒)4.1 ± 0.8(‒6) µm, length/septum width ratio (3.2–)3.9 ± 0.6(‒5.3) (n = 162; N = 9). Pycnidia usually abundant, ostioles red, more or less immersed, distinct or almost invisible, conidia short to long bacilliform, (2.8‒)3.6 ± 0.3(‒4.8) (n = 277) × (0.6‒)1.1 ± 0.2(‒1.5) (n = 204) µm, length/width ratio (2.7–)3.3 ± 0.4(‒3.8) (N = 9).

*Distribution and ecology: Wetmoreana sliwae* is known from Bolivia and Peru, where it occurs in semi-desert, high mountain regions at an altitude between 3462 and 4437 m. It grows on siliceous rocks, in well-lit conditions.

*Notes: Wetmoreana sliwae* is characterized by a stipitate squamulose, orange to orange-red thallus, abundant, red, clearly zeorine apothecia, and abundant pycnidia with red ostioles. The specimens with less developed thalli may resemble *Calogaya miniata* (Fig. 9b), while those with well-developed thalli are rather unique among *Teloschistaceae*. In general, *W. sliwae* is similar to *Squamulea* (*Xanthorioideae*) due to the squamulose type of thallus. However, this is only a superficial, generalized similarity. The new species differs from *Squamulea* by having thicker, distinctly stipitate-squamulose thalli (which is mainly areolate to subsquamulose in *Squamulea*), a verruculose surface of the squamules, large apothecia, distinct group arrangements of algae in the thallus, prospolectenchymateous thalline cortex and exciple of the apothecia, and larger and wider ascospores. An exception is *S. squamosa* which stands apart from other members of *Squamulea*, and is most similar to *W. sliwae* due to its fairly well-developed ascending and subimbricate thalli, large apothecia (up to 2 mm in diameter) with a clearly visible thalline margin, and algae often clustered in columns (Wetmore 2003). *Squamulea squamosa* differs from *W. sliwae* by producing noticeably smaller ascospores with thinner septa (9.5–14.0 × 5.5–7.0 µm, septa 3.0–4.0 µm vs. 12–22 × 5–9 µm, septa 2–6 µm; Wetmore 2003), in addition to the other characters mentioned above. Finally, *W. sliwae* is quite similar to a newly recognized member of *Squamulea*, *S. evolutior*. That species differs from *W. sliwae* in having a thinner thallus (120–170 µm vs. 250‒850 µm in *W. sliwae*), black prothallus, plane surface of the squamules, paraplectenchymateous thalline cortex and exciple, continuous algal layer in the thallus, sessile and smaller apothecia (0.1–0.8 mm vs. 0.2–1.2 mm in diameter), thin apothecial margins (below 100 µm), and distinctly shorter ascospores (8‒13 µm vs. 12‒22 µm) with a different shape (widely ellipsoid vs. regularly ellipsoid).

*Calogaya miniata* differs from the less well-developed specimens of *W. sliwae* by having a crustose, lobate thallus with areoles in the central portion, continuous algal layer in the thallus, smaller ascospores (7–15 µm length vs. 12‒22 µm), lack of pycnidia, and different ecology, occurring on calcareous rocks.

Among *Wetmoreana*, *W. sliwae* may be confused with *W. brachyloba* but differs in having a quite thicker (150‒850 µm vs. 90‒200 µm) and distinctly squamulose thallus (vs. areolate to subsquamulose), verruculose thalline surface with rather abundant conspicuous small cracks (pseudocyphellae?), well-developed necral layer, algae clustered in distinct groups or columns, distinctly larger apothecia (0.2–1.2 mm vs. 0.2–0.8 mm in diameter), usually cracked apothecial discs, quite thicker amphithecium (over 100 µm vs. below 100 µm), ascospores that are more variable in size, thinner ascospore septa (mean thickness 4.1 µm vs. 5.2 µm), pycnidia with red ostioles, and in the absence of any crystals prominent in polarized light in the thallus and apothecia.

A specimen from Chile from the Juan Fernandez Islands collected by C. & I. Skottsberg (S L2589) was grouped within the *W. sliwae* clade in the PBPB analysis (ML, BS = 100; MP, PB = 93) (Fig. 2, Additional file 7: Fig. S1). It was considered to represent *W. sliwae*, but there are several phenotypic differences suggesting that the Chilean specimen should be regarded as a separate undescribed species. The Chilean specimen differs from *W. sliwae* in having a relatively thinner thallus (150‒170 µm thick) without any cracks on its surface, paraplectenchymatous thalline cortex, the algal layer more-or-less continuous (almost all specimens of *W. sliwae* have algae in distinct groups or columns), sessile and slightly smaller apothecia (up to 0.8 mm), thinner apothecial margins, pycnidia with orange ostioles, and shorter conidia (mean length 3.1 µm). This specimen is a syntype of *Caloplaca rubina* var. *evolutior* but differs from the holotype of *Squamulea evolutior* by its undulate or verruculose surface of the squamules, absence of a prothallus, prosoplectenchymateous exciple, and larger ascospores (11–16 µm × 6–10 µm length vs. 8‒13 × 5–8 µm) (see comments under *S. evolutior* below).

*Additional material examined:* **Bolivia:** Dept. Oruro, Prov. Sajama, Parque Nacional Sajama: Jecha K’ala 25 km of Sajama village, Puna sureña, Pajonales vegetation, alt. 4184 m, 18°09′52″S 68°49′08″W, 20 June 2010, *A. Flakus* *16,719 & P. Rodriguez* (LPB); near Sajama village, Puna sureña, Tholares vegetation, alt. 4437 m, 18°07′49″S 68°56′54″W, 18 June 2010, *A. Flakus 16,503 & P. Rodriguez* (KRAM, LPB). Dept. La Paz, Prov. Manco Kapac near Copacabana village, Mt. Horca del Inca, high Andean Puna vegetation, alt. 3974 m, 16°10′15″S 69°05′05″W, 18 June 2006, *A. Flakus 8667* (KRAM, LPB, B); Dept. La Paz, Prov. Franz Tamayo, Pelechuco, slope N, along river, sparse bushes, sun-exposed and humid place, alt. 3500 m 14°49′21″S 69°04′06″W, 13 Oct. 2007, *K. Wilk 7511* (KRAM, LPB). **Chile:** Juan Fernandez, Masafuera, Quebr. de las Casas, 19 Feb. 1917, *C. & I. Skottsberg s.n.* (S L2589, as *Caloplaca rubina* var. *evolutior* syntype). **Peru:** Dept. Arequipa, Prov. Caylloma. Valle del Colca valley, near Achoma village, open semi-desert montane area, alt. 3500 m, 15°39′26″S 71°43′38″W, 2 July 2006, *A. Flakus 9266, 9277, 9278 & B. Cykowska* (KRAM, LPB, B). Cañon del Colca canyon, near Cabanaconde village, open semi-desert montane area: alt. 3480 m, 15°38′18″S 71°57′43″W, 5 July 2006, *A. Flakus 9600 & B. Cykowska* (KRAM, LPB, B); alt. 3462 m, 15°37′56″S 71°57′49″W, 4 July 2006, *A. Flakus 9526 & B. Cykowska* (KRAM, B).

#### ***Wetmoreana sliwae*** subsp. ***subparviloba*** Wilk & Lücking, subspec. nov. (Fig. 9i–l)

*MycoBank:* MB851731.

*Etymology:* The epithet is derived from similarities to *Squamulea parviloba*.

*Diagnosis:* Similar to *Wetmoreana sliwae* but thallus smooth, squamule lobulated at margins, apothecia uncommon and smaller, 0.3‒0.6 mm versus 0.2‒1.2 mm wide, ascospores smaller, 9‒13 × 5‒7 µm versus 12‒22 × 5‒9 µm, ascospore septa thinner, 2‒3 µm versus 2‒6 µm and pycnidia not abundant.

*Type:* **Peru:** Dept. Arequipa, Prov. Caylloma, Valle del Colca valley, near Achoma village, open semi-desert montane area, alt. 3500 m, 15°39′26"S, 71°43′38"W, 2 July 2006, *A. Flakus 9274 & B. Cykowska* (KRAM L 71776—holotype, LPB, B—isotypes).

*Description:* Thallus squamulose, irregular in outline, loosely attached to substrate, pale to dark orange or reddish, K + purple, epruinose to somewhere white pruinose, smooth, parasitized by fungi, squamule stipitate, growing irregularly, strongly convex, 225–380 µm thick, margins of squamules crenate to lobulate, lobules robust, finger-like, short; vegetative propagules absent; prothallus absent, upper cortex 20‒100 µm thick, prosoplectenchymatous, uneven with distinct cortex cones, necral layer present, discontinuous, indistinct, 3.5‒12 µm thick, colorless crystals absent; algal layer discontinuous, algae in distinct groups or irregularly arranged; medulla without colorless crystals. Apothecia few or absent, dispersed or crowded, rounded or angular, erumpent to sessile, zeorine, 0.3‒0.6 mm in diam.; disc first concave then plane, dark orange, K + purple, darker than thallus, epruinose, entire; both margins distinguishable but inconspicuous, prominent, proper margin thick and prominent in young apothecia, then thin and only slightly prominent, ± concolorous with disc, thalline margin much reduced, visible at the base of apothecia, even; parathecium dominant, 70‒170 µm thick, prosoplectenchymateous, with distinct long lumina cells; amphithecium 68–119 µm thick, algae abundant, continuous to discontinuous, apothecial cortex indistinct, thin, ca. 25 µm thick, colorless crystals absent; epihymenium yellow orange, K + purple; hymenium 80–100 µm thick; hypothecium ca. 102 µm thick, cone-shaped, prosoplectenchymatous, hyaline, oil droplets present; paraphyses simple to slightly branched, distinctly thick (not slender), 2 µm broad at base, with upper cell slightly wider, up to 2‒5 µm, oil droplets absent; asci 8-spored; ascospores hyaline, regularly ellipsoid, thin-walled, (9‒)11.1 ± 1.2(‒13) × (5‒)5.9 ± 0.6(‒7.0) µm, length/width ratio 1.9, ascospore septa (2‒)2.8 ± 0.4(‒3) µm, length/septum width ratio 4.0 (n = 15; N = 1). Pycnidia ± abundant, ostioles red, almost invisible, conidia short to long bacilliform, (3.0‒)3.5 ± 0.4(‒5.0) (n = 39) × (0.6‒)1.0 ± 0.2(‒1.2) (n = 35) µm, length/width ratio (3.2–)3.6 ± 0.6(‒4.0) (N = 2).

*Distribution and ecology: Wetmoreana sliwae* ssp. *subparviloba* is only known from Peru, where it occurs together with *W. sliwae* in semi-desert habitats in high mountain regions at an altitude of 3500 m. It grows on siliceous rocks in well-lit conditions.

*Notes: Wetmoreana sliwae* ssp. *subparviloba* differs from *W. sliwae* s.str. by having an irregularly growing squamulose thallus, consisting of strongly convex, shiny squamules with finger-like lobules on their edges, smaller, erumpent to sessile apothecia (0.3–0.6 mm vs. 0.2–1.2 mm in diameter), and distinctly smaller ascospores (9–13 µm length vs. 12‒22 µm) with thinner septa (2–3 µm vs. 2‒6 µm). Even though the two taxa are not separated in our phylogenetic tree (Fig. 3), we decided to treat them as distinct at the subspecific level. We are aware that the differences in the thallus morphology may be the result of the habitat conditions, but the taxa also differ in other features that are statistically significant, such as size of ascospores. Therefore, we felt that it would be better to show these differences for further verification by specialists.

*Wetmoreana sliwae* ssp. *subparviloba* is similar to *Squamulea parviloba* but the latter is lobate in the thallus margin, has plane squamules, a paraplectenchymatous thalline cortex and exciple, ± persistent to partly reduced thalline margins of the apothecia, and distinctly thicker ascospore septa (2.5–6 µm vs. 2‒3 µm). In addition, *S. parviloba* has delicate, noticeably smaller lobules compared to *W. sliwae* ssp. *subparviloba*. The lobules of the latter species are thick and robust.

*Additional material examined:* **Peru:** Dept. Arequipa, Prov. Caylloma, Valle del Colca valley, near Achoma village, open semi-desert montane area, alt. 3500 m, 15°39′26″S 71°43′38″W, 2 July 2006, *A. Flakus 9268 & B. Cykowska* (KRAM, LPB, B).

#### ***Wetmoreana variegata*** Wilk & Lücking, sp. nov. (Fig. 10)

**Fig. 10:**
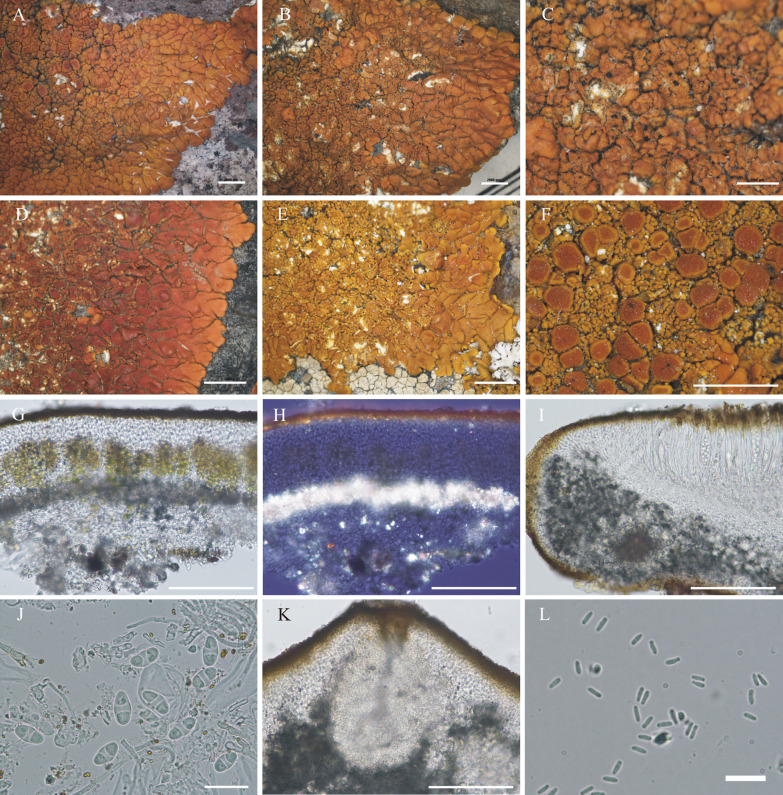
*Wetmoreana variegata*. **A** fertile thallus (holotype), **B** thallus with schizidia (*A. Flakus* 9414, KRAM), **C** close-up on schizidia (*A. Flakus 9414*, KRAM),** D** thallus with isidia resembling *W. ochraceofulva* (*K. Wilk 4144*, KRAM), **E**–**F** untypical specimen from Ecuador showing thallus with apothecia and schizidia/isidia growing together (*R. Davis 34*, BM), **G**–**H** sections of thallus showing calcium oxalate crystals below algal layer prominent in polarized light (**H**) (*K. Wilk 4144*, KRAM), **I** section of apothecium showing prosoplectenchymatous parathecium, **J** ascospores, **K** section of pycnidium, **L** conidia (*A. Flakus 9643*, KRAM). Scale in **A**, **B**, **D**, **E**, **F** = 2 mm, in **C** = 1 mm, in **G**, **H**, **I**, **K** = 100 µm, in **J** = 20 µm, in **L** = 10 µm

*MycoBank:* MB851732.

*Etymology:* The epithet is derived from the high phenotypic variability of the species.

*Diagnosis:* Similar to *Wetmoreana ochraceofulva* but thallus often fertile, when sterile bearing schizidia, rarely isidia, ascospore septa thicker, 3–5 µm versus 2‒4 µm and occurring in South America versus mainly Africa.

*Type:* **Peru:** Dept. Arequipa, Prov. Caylloma, Cañon del Colca canyon, near Cabanaconde village by the way from Cabanaconde to San Juan de Chuccho, open semi-desert montane area, alt. 3179 m, 15°36′11″S 71°57′05″W, 6 July 2006, *A. Flakus 9643 & B. Cykowska* (KRAM L 71778—holotype, LPB—isotype).

*Description:* Thallus crustose, lobate, areolate in central part, ± orbicular to irregular in outline, up to 5.0 cm wide, tightly attached to substrate, yellow orange, orange or reddish orange, often paler at lobe tips, K + purple, epruinose, matte, sometimes slightly wrinkled, often parasitized by fungi (*Zwackhiomyces*), areoles polygonal to granular, 0.2‒1.0 mm wide, plane to convex, marginal lobes plane to sometimes convex, widened at the tips, 0.2‒4.5 mm long, 0.1‒2.0 mm wide at the tips, 125‒600 µm thick; vegetative propagules present almost exclusively on sterile thalli, schizidia, rarely isidia, concolorous with thallus, abundant, marginal, laminal, often areoles dissolved into propagules, sometime areole turned upwards; prothallus absent; upper cortex 15‒110 µm thick, paraplectenchymatous, uneven, distinct cortex cones present, necral layer usually absent, colorless crystals absent; algal layer discontinuous, algae in distinct columns or groups; medulla prosoplectenchymatous, thick, always with CaOx crystals forming a limited layer 10‒85 µm wide at the base of algal layer. Apothecia absent or present, scarce to abundant, crowded, rounded to angular when compressed, erumpent to sessile, zeorine, 0.2‒0.9 mm in diam.; disc first concave, then plane, orange, darker than thallus, K + purple, epruinose, usually cracked; both margins distinguishable but inconspicuous or almost indistinguishable, apothecial margin thick, prominent or rarely to level with disc, proper margin paler than disc, thalline margin ± persistent to partly reduced, sometimes much reduced, even or rarely crenate in old apothecia; parathecium 50‒119 µm thick, prosoplectenchymatous; amphithecium 60‒128 µm thick, algae abundant, ± discontinuous, apothecial cortex indistinct, 12‒26 µm thick, consist of small isodiametric cells, colorless crystals absent; epihymenium yellow orange, K + purple; hymenium 70‒102 µm thick; hypothecium ca. 140 µm thick, prosoplectenchymatous, hyaline, oil droplets absent; paraphyses simple to rarely slightly branched, 1‒2 µm broad at base, with upper cell not or slightly wider, 2‒4(‒5) µm, oil droplets absent; CaOx crystals present in the medulla of apothecia; asci 8-spored; ascospores hyaline, regularly ellipsoid, (10‒)13.1 ± 1.5(‒17) × (5‒)6.3 ± 0.7(‒9.0) µm, length/width ratio (2.0–)2.2 ± 0.1(‒2.3), ascospore septa (3‒)3.6 ± 0.5(‒5) µm, length/septum width ratio (3.2–)3.6 ± 0.4(‒4.1) (n = 125; N = 6). Pycnidia usually abundant, often almost invisible, ostioles red, conidia ovoid to short bacilliform, (1.9‒)3.2 ± 0.4(‒4.2) (n = 373) × (0.8‒)1.2 ± 0.2(‒1.7) (n = 339) µm, length/width ratio (2.2–)2.8 ± 0.5(‒3.9) (N = 14).

*Distribution and ecology: Wetmoreana variegata* is known from Argentina, Bolivia, Ecuador and Peru, where it occurs in semi-desert, high mountain regions at an altitude between 2774 and 3349 m. It grows on siliceous, rarely calcareous rocks, in well-lit conditions, and it is often accompanied by *Squamulea subsoluta*.

*Notes: Wetmoreana variegata* is a highly variable species that, together with *W. ochraceofulva* forms the newly recognized *W. ochraceofulva* complex (Fig. 3, Table 3). *Wetmoreana variegata* is characterized by a rather large, orange yellow, lobate thallus, the presence of apothecia or schizidia or rarely isidia in the central portion of thallus; both reproductive structures, such as apothecia and propagules, are rarely present on the same specimen. The algal layer in the thallus is always discontinuous and consists of algae gathered in distinct columns, and CaOx crystals are always present as a limited crystalline layer at the base of the algal layer and in the apothecial medulla.

Fertile specimens of *W. variegata* are superficially very similar to *W. appressa*. In contrast, sterile ones resemble *W. ochraceofulva* or *W. texana*. There is one atypical specimen from Ecuador (*R. Davis 34*, BM; not sequenced), representing very well-developed thallus in which apothecia, and isidia and schizidia grow together. The type of *W. appressa* differs from *W. variegata* in having a more yellowish thallus, continuous algal layer, entire apothecial disc surface, and ascospores with distinctly thicker septa (4.5‒9.0 µm vs. 3‒5 µm in *W. variegata*). *Wetmoreana texana* has a thallus tightly to loosely adhering to the substrate, more yellowish in color and partly white pruinose, without any cracks on its surface, more convex marginal lobes, fewer apothecia when present, and the joint occurrence of apothecia and vegetative propagules. Moreover, *W. texana* differs from *W. variegata* by having ± widely ellipsoid ascospores with thicker septa (4.4 µm vs. 3.6 µm on average), distinctly longer conidia (3.8–4.4–4.9 µm length vs. 2.8–3.2–3.6 µm on average), and CaOx crystals that are not always present but if so, then not forming a distinct limited layer at the base of the algal layer. The two species are not closely related, and *W. texana* is sister to *W. sliwae* (Fig. 3). *Wetmoreana ochraceofulva* is distinguished from *W. variegata* by consistently forming isidia which occur together with apothecia, if the latter are present (they are extremely scarce). In contrast to *W. variegata*, the isidia of *W. ochraceofulva* are usually rounded, distinctly smaller, almost indistinguishable from each other at low magnification, and formed at the margins of areoles in a regular pattern. The ascospores of *W. ochraceofulva* have septa slightly thicker than in *W. variegata*, and the length/septum width ratio is higher (Table 3). Finally, *W. ochraceofulva* is a morphologically uniform species found mainly on the African continent, while *W. variegata* is highly variable and only occurs in South America (Fig. 6). *Wetmoreana variegata* may also be confused with *W. brouardii*. The latter species, however, differs from *W. variegata* in having smaller marginal lobes [0.4‒2(‒2.2) mm long and 0.2‒1 mm wide at tips vs. 0.2‒4.5 mm long and 0.2‒1.6(‒2) mm wide] that are thinner (90–250 µm vs. 125–600 µm), and more closely adhering to the substrate. Moreover, apothecia in *W. brouardii* are extremely rare, and when present the ascospores are smaller ascopores (10–13 µm length vs. 10–17 µm) with thinner septa (2–3.5 vs. 3–5 µm); also, abundant distinctive small papillae always cover the thallus surface. Finally, the algae form a continuous layer or are clustered but not in such distinct groups as in *W. variegata*, and CaOx crystals are not present in the medulla. *Wetmoreana circumlobata* differs from *W. variegata* in having slightly shorter marginal lobes (0.3–1.4 mm vs. 0.2–4.5 mm length), strongly convex central areoles, a continuous to discontinuous algal layer in the thallus (algae not forming distinct columns), sessile apothecia with entire apothecial discs, more reduced thalline apothecial margin, thicker parathecium (up to 190 µm vs. up to 119 µm), larger and wider ascospores [(13–)16.2(–19) × (7–)7.5(–9) µm vs. (10–)13.1(–17) × (5–)6.3(–9) µm] with thicker septa (mean thickness 4.6 µm vs. 3.6 µm), long bacilliform conidia (length/width ratio 3.8 vs. 2.7), and lack of vegetative propagules.

Although *W. variegata* and *W. ochraceofulva* are not well separated in our molecular and integrative approaches, the emerging patterns, especially considering vegetative propagules and ascospore morphology, led us to recognize *W. variegata* as a distinct taxon. Its recognition will also help to test the taxonomy of the two species with further data in the future, rather than encourage premature lumping.

*Additional material examined:* **Argentina:** Prov. Jujuy, Casabindo, alt. 3600 m, 12? Jan. 1948, *P.J. Santesson 125* (BM 1247497, as *C. subnitida*). **Bolivia:** Dept. Cochabamba, Prov. Quillacollo, East Cordillera, area of Inkarraya-Sipesipe, semidesert Inter-Andean Valleys, alt. 3146 m, 17°29′25"S 66°22′09"W, rocky and shrubby slope, sunny place, E exposition, 17 Dec. 2004, *K. Wilk 3277* (as *C. ochraceofulva* in Wilk and Flakus 2017), 3286 (KRAM, LPB, B); 2846 m, 17°28′39"S 66°21′43"W, NE exposition, 17 Dec 2004, *K. Wilk 3291*, *3293a* (KRAM, LPB, B). Dept. La Paz, Province Bautista Saavedra, Charazani town, by the road, open place, 13 May 2006, *K. Wilk 4144* (KRAM, as *C. ochraceofulva* in Wilk and Flakus 2017). Dept. Potosi, Province Nor Lipez, Pinturas Rupestres near Mallku Villamar village, alt. 4038 m, 21°46′20"S 67°29′05"W, high Andean area, 6 Dec. 2009, *A. Flakus 14,802/1*, *A. Flakus 14,812 & P. Rodriguez* (KRAM, LPB, as *C. texana* in Wilk and Flakus 2017). **Chile:** Tarapacá, Cerro Grande near Marmiña, alt. 300 m, 20°05′S 69°11′W, May 1961, *G. Follmann 7673* (B 600180162). **Ecuador:** Prov. Loja, Gorge of Rio Oña, 1,5 km west of Oña, alt. 2200 m, fairly arid area, 10 Aug. 1980, *R. Davis 34* (BM1247492). **Peru:** [Dept. Lima, Province Huarochirí], Matucana, alt. ca. 2438 m (8000 feet), 14–18 March 1923, *G. S. Bryan 35* (F, as *C. murorum*). Dept. Arequipa, Prov. Caylloma. Valle del Colca valley, near Soccoro village, alt. 3349 m, 15°38′32″S 71°43′22″W, open semi-desert montane area, 3 July 2006, *A. Flakus 9404, 9408, 9411, 9414, 9415a, 9415b & B. Cykowska* (KRAM, LPB, B, as *C. texana* in Wilk & Flakus 2017). Cañon del Colca canyon, below Tapay village, alt. 2774 m, 15°35′07″S 71°56′37″W, open semi-desert montane area, 6 July 2006, *A. Flakus 9662 & B. Cykowska* (KRAM, B).

### New combinations

#### ***Wetmoreana awasthii*** (Y. Joshi & Upreti) Wilk & Lücking, comb. nov.

*MycoBank:* MB851733.

*Basionym: Caloplaca awasthii* Y. Joshi & Upreti, Botanical Journal of the Linnean Society 155 (1): 149 (2007).

*Type:* **India:** Madhya Pradesh, Raisen district, Bhimbetka, Rang Mahal Area, on rocks, alt. 500 m, 2 Nov. 2004, *Y. Joshi 04–004539* (LWG—holotype).

*Synonym: Fulgogasparrea awasthii* (Y. Joshi & Upreti) S.Y. Kondr. et al., in Mishra et al., Acta Botanica Hungarica 62: 349 (2020).

*Notes:* The placement of *W. awasthii* within *Wetmoreana* (= *Fulgogasparrea*) is uncertain. The sequences of this species were published by Mishra et al. (2020) are not present in GenBank. Therefore, the molecular study must confirm *W. awasthii* placement within the genus *Wetmoreana*.

#### ***Wetmoreana brachyloba*** (Müll. Arg.) Wilk & Lücking, comb. nov. (Fig. 9m–t).

*MycoBank:* MB851734.

*Basionym: Amphiloma brachylobum* Müll. Arg., Rev. Mycol. (Toulouse) 10: 59 (1888).

*Type:* **Paraguay:** Asuncion, 1887, *[B. Balansa] 4200* (G00290940!—lectotype; see Wetmore, Bryologist 106: 155. 2003).

*Synonyms: Callopisma brachylobum* (Müll. Arg.) Malme, Ark. Bot. 20A(9): 40 (1926); *Caloplaca brachyloba* (Müll. Arg.) Zahlbr., Cat. Lich. Univers. 7: 219 (1931).

*Description*: Thallus crustose-areolate to squamulose, irregular in outline, tightly attached to substrate, yellow to yellow orange, K + purple, epruinose, without pseudocyphellae or other cracks, areoles/squamules mostly plane, rarely slightly convex in central part of thallus, 0.2–0.5 mm in diam., 140–200 µm thick, squamlue crenate, marginal lobes absent; vegetative propagules absent; prothallus absent; upper cortex 10‒50 µm thick, paraplectenchymatous, uneven, some cortex cone present, necral layer absent or somewhere present, 5–12 µm wide, colorless crystals absent; algal layer continuous to discontinuous; medulla thin, always filled with CaOx crystals. Apothecia abundant, crowded, round to angular, initially immersed then erumpent, zeorine, 0.2‒0.8 mm in diam.; disc first concave then plane even in old apothecia, darker than thallus, K + purple, epruinose, entire (without cracks); both margins distinguishable but inconspicuous, proper margin prominent, paler than disc, to ± level with disc and darkening in old apothecia, thalline margin partly to much reduced, even; prathecium 70‒150 µm thick, prosoplectenchymatous; amphithecium 70‒130 µm thick, algae abundant, apothecial cortex distinct, 15‒20 µm thick, paraplectenchymatous, colorless crystals absent; epihymenium yellow brown, K + purple; hymenium 90‒110 µm thick; hypothecium 25–140 µm thick, prosoplectenchymatous, hyaline, oil droplets absent; paraphyses simple, 1–1.5 µm broad at base, with upper cell 2‒5 µm wide; CaOx crystals alaways present in the medulla of apothecia; asci 8-spored; ascospores hyaline, regularly ellipsoid, thin-walled, (12‒)16.0 ± 1.6(‒20.5) × (6‒)7.7 ± 1.0(‒10.0) µm, length/width ratio (1.9–)2.1 ± 0.3(‒2.3), ascospore septa (3‒)5.2 ± 0.9(‒7) µm, length/septum width ratio (3.0‒)3.2 ± 0.3(‒3.4) (n = 75; N = 2). Pycnidia few to abundant, ostiole orange, slightly raised, distinct, conidia short to long bacilliform, (2.7‒)3.7 ± 0.4(‒4.5) (n = 62) × (0.7‒)1.1 ± 0.1(‒1.5) (n = 78) µm, length/width ratio (3.3–)3.5 ± 0.4(‒3.7) (N = 2).

*Distribution and ecology:* The species is known only from Paraguay, where it grows on siliceous, sandstone rocks.

*Notes:* The studied material of *W. brachyloba* is morphologically uniform. The species has some similarities with *W. sliwae*, as both have a ± squamulose thallus and produce similar ascospores. For a detailed comparison, see the discussion of *W. sliwae*. *Wetmoreana brachyloba* is also similar to *W. circumlobata* in anatomical aspects of thallus and apothecia, including the size of ascospores and conidia (see comments under *W. circumlobata*).

Malme (1926) proposed the new combination *Callopisma brachylobum* for *A. brachylobum*. Notable, the material studied by Malme (1926) is not conspecific with *A. brachylobum* (G, BM, and M) and represents an undescribed species. The latter is described above as new species, *W. circumlobata* (see also the commentary under this species). Nevertheless, since nomenclatural acts follow types, the combination of *A. brachlobum* into *Callopisma* by Malme is valid.

*Additional material examined:* **Paraguay:** Asuncion, June 1879, *B. Balansa 4200* (M, BM, G00293574, G00290942), 1887, *B. Balansa 4200* (G00290941), and 1888, *B. Balansa 4200* (G00290943).

#### ***Wetmoreana chapadensis*** (Malme) Wilk & Lücking, comb. nov. (Fig. 11).

**Fig. 11:**
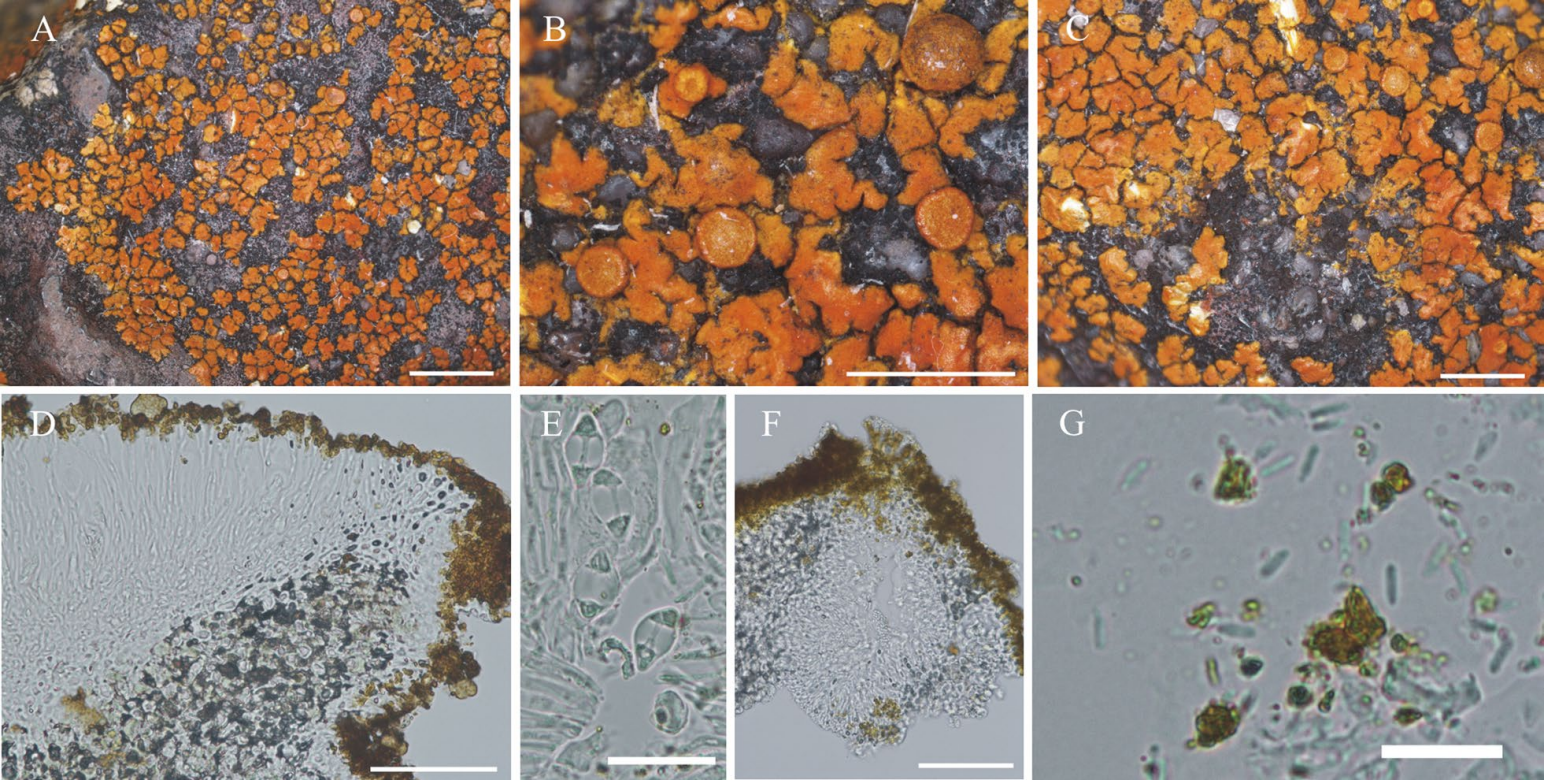
*Wetmoreana chapadensis* (holotype). **A** thallus habit, **B** close-up on apothecia, **C** orange prothallus, **D** section of apothecium showing prosoplectenchymatous parathecium, **E** ascus with spores, **F** section of pycnidium, **G** conidia. Scale in **A**, **B**, **C** = 2 mm, in **D**, **F** = 100 µm, in **E** = 20 µm, in **G** = 10 µm

*MycoBank:* MB851735.

*Basionym: Callopisma chapadense* Malme, Ark. Bot. 20A(9): 38 (1926).

*Type:* **Brazil:** Brasiliæ civit, Matto Grosso: Serra da Chapada, in colli africo, in campo limpo, 20 Jan. 1894, *G. O. Malme 2318* (S L12852!—holotype).

*Synonym: Caloplaca chapadensis* (Malme) Zahlbr., Cat. Lich. Univers. 7: 223 (1931).

*Discription*: Thallus crustose, areolate in centre, sublobate at margin, irregular in outline, tightly attached to substrate, reddish orange, K + purple, epruinose, without pseudocyphellae or other cracks, areoles mostly sublobate, plane to convex, marginal sublobes plane, widened at tips, 0.2‒0.5 mm long, 0.1‒0.4 mm wide at the tips, 170 µm thick; vegetative propagules absent; prothallus conspicuous, orange; upper cortex 8.5‒17 µm thick, paraplectenchymatous, necral layer absent, colorless crystals absent; algal layer continuous; medulla thin, colorless crystals absent. Apothecia scarce, scattered, round, sessile, zeorine, 0.2‒0.7 mm in diam.; disc plane to slightly convex in old apothecia, ± concolorous with thallus, K + purple, epruinose, entire (without cracks); both margins not or almost not distinguishable from each other’s, apothecial margin very thin and level with disc in mature apothecia, concolorous with disc, thalline margin partly reduced, even; parathecium 55‒75 µm thick, prosoplectenchymateous; amphithecium 65‒70 µm thick, algae abundant, discontinuous in the central part, apothecial cortex distinct, 15–25 µm thick, paraplectenchymatous, colorless crystals absent; epihymenium yellow orange, K + purple; hymenium 65‒80 µm thick; hypothecium 50–70 µm thick, prosoplectenchymatous, hyaline, oil droplets absent; paraphyses simple, with upper cell 3 µm wide; asci 8-spored; ascospores hyaline, regularly ellipsoid, thin-walled, (11‒)13.2 ± 1.1(‒15) × (6‒)6.9 ± 0.6(‒8.0) µm, length/width ratio 1.9, ascospore septa (5‒)6.2 ± 0.8(‒7) µm, length/septum width ratio 2.1 (n = 12; N = 1). Pycnidia abundant, ostiole red, raised, indistinct, conidia long bacilliform, (2.6‒)3.2 ± 0.2(‒3.6) (n = 48) × (0.6‒)0.8 ± 0.2(‒1.2) (n = 43) µm, length/width ratio 4.0 (N = 1).

*Distribution and ecology: Wetmoreana chapadensis* is known only from Brazil (Matto Grosso), where it was collected on siliceous stones in a sunny field covered by grasses and herbs (Malme 1926).

*Notes: Wetmoreana chapadensis* is a rather distinct species, clearly separated from other *Wetmoreana* spp. The PBPB analysis indicates that *W. chapadensis* is closest to *W. rubra*, but it differs from the latter in having an orange prothallus, mostly sublobate central areoles (vs. polygonal), sessile apothecia (vs. erumpent), apothecial discs ± concolorous with the thallus, and lack of a clear distinction between proper and thallus margin of the apothecia; in addition, it differs by the indistinct pycnidia, ascospores with distinctly thicker septa (5–7 µm vs. 1–2 µm), thin thalline cortex not covered by a necral layer, and lack of CaOx crystals in the thallus and apothecia. *Wetmoreana chapadensis* can be confused with *W. bahiensis* and *W. subnitida*. Both of those species have a more-or-less reddish thallus. However, *W. chapadensis* differs from the latter two species in having an obscurely lobate thallus, sublobate central areoles, the presence of prothallus, and larger ascospores with distinctly thicker septa.

#### ***Wetmoreana intensa*** (Aptroot & M. Cáceres) Wilk & Lücking, comb. nov.

MycoBank: MB851736.

*Basionym: Fulgogasparrea intensa* Aptroot & M. Cáceres, Cryptogamie, Mycologie 42 (11): 183 (2021).

*Type:* **Brazil:** Sergipe, Poço Redondo, Cajueiros, Trilha Ecoparque, 09°39′43″S, 43°40′18″W, on exposed granite, 15 Nov. 2018, *M. Cáceres & A. Aptroot s.n.* (ISE 48030—holotype, ABL—isotype).

*Notes: Wetmoreana intensa* is closely related to *W. brouardii*, hence belongs to the newly recognized *W. brouadrii* complex. *Wetmoreana intensa* is very similar to *W. bahiensis* and *W. subnitida*. For comparisons with these species, see notes under the corresponding taxa.

#### ***Wetmoreana ochraceofulva*** (Müll. Arg.) Wilk & Lücking, comb. nov. (Fig. 12).

**Fig. 12:**
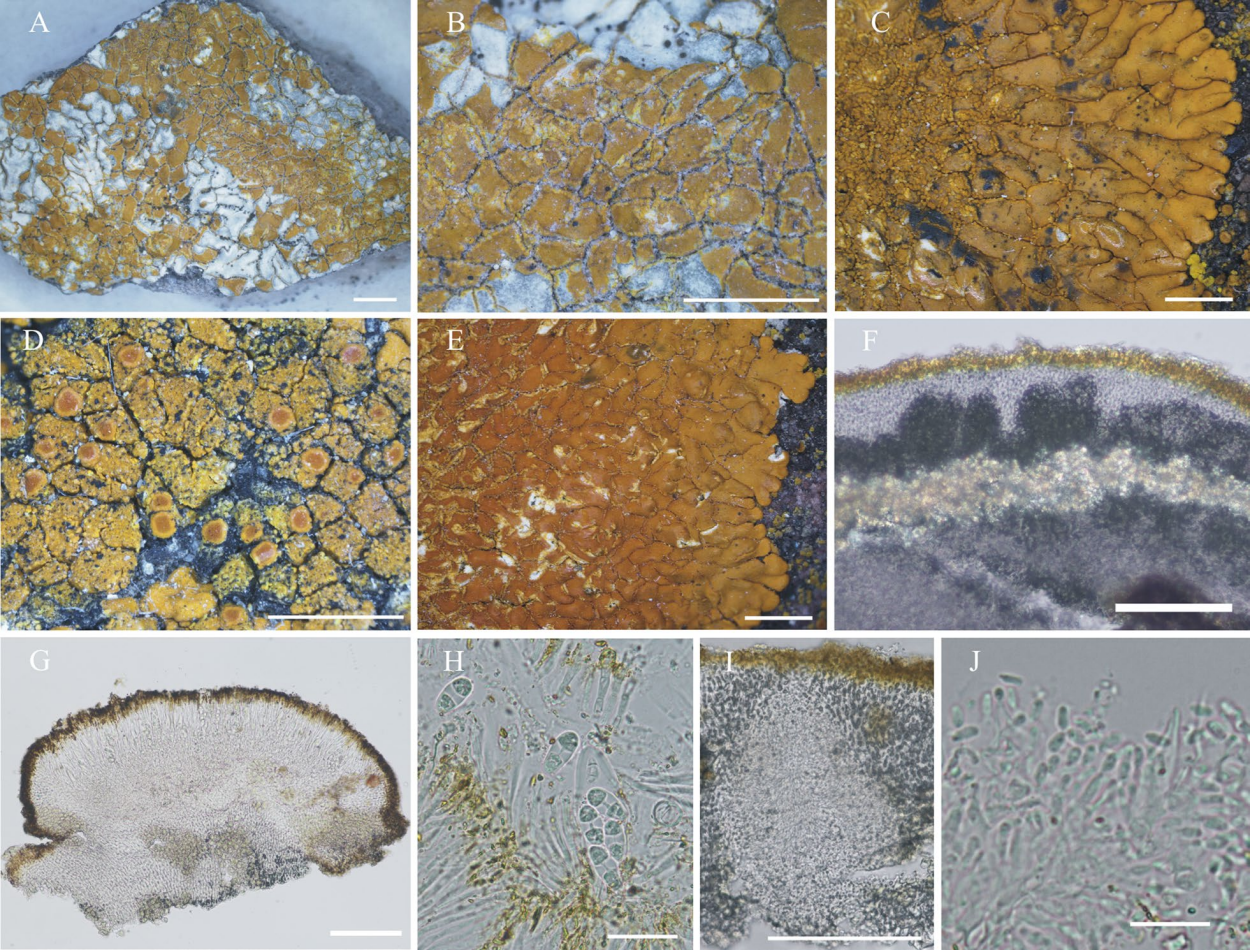
*Wetmoreana ochraceofulva*. **A** lectotype showing incomplete specimen lacking marginal lobes, **B** close-up on marginal isidia (lectotype), **C** well-developed thallus (*R. A. Maas Geesteranus 10,299*, LD), **D** close-up on apothecia growing on isidiate thallus (*O. Almborn*, LD 1024960), **E** specimen from Argentina (*R. E. Fries 50*, LD 1076640), **F** section of thallus showing calcium oxalate crystals below algal layer prominent in polarized light, **G** section of apothecium showing prosoplectenchymatous parathecium, **H** ascus with spores, I section of pycnidium, **J** conidia (*O. Almborn*, LD 1024960). Scale in **A**, **B**, **C**, **D**, **E**, **E** = 2 mm, in **F**, **G**, **I** = 100 µm, in **H** = 20 µm, in **J** = 10 µm

*MycoBank:* MB851737.

*Basionym: Amphiloma ochraceofulvum* Müll. Arg., Flora (Regensburg) 68 (28): 504 (1885).

*Type:* **Somalialand:** Serrubgebirge, 1885, *J. M. Hildebrandt s.n.* (G 00066459!—lectotype; see Kärnefelt, Lichenologist 22: 314. 1990).

*Description*: Thallus crustose, lobate, areolate in central part, orbicular to irregular in outline, tightly attached to substrate, yellow orange to orange, K + purple, epruinose, sometimes with sall cracks on the thalline surface, areoles polygonal, plane, marginal lobes plane, widened at tips, 0.3‒2.4 mm long, 0.2‒1.7 mm wide at the tips, (50)150–400 µm thick; vegetative propagules always present, isidia marginal, sometimes laminal or thallus is dissolved into isidia; prothallus absent; upper cortex 8.5‒80 µm thick, paraplectenchymatous, necral layer absent, colorless crystals absent; algal layer discontinuous, algae in distinct groups or columns; medulla prosoplectenchymatous, always with CaOx crystals forming a limited layer 17–77 µm wide and µm at a distance of 85–120 from the thallus surface, at the base of the algal layer. Apothecia mostly absent, few to scarce, scattered to crowded, round, erumpent to sessile, zeorine, 0.2‒0.6 mm in diam.; disc plane even in old apothecia, ± concolorous with thallus or darker than thallus, K + purple, epruinose, slightly cracked; both margins not or almost not distinguishable from each other’s or distinguishable but inconspicuous, proper margin slightly prominent to level with disc, paler than disc, thalline margin ± persistent to partly reduced or much reduced, even; parathecium dominate, 70‒150 µm thick, prosoplectenchymateous; amphithecium 35‒80 µm thick, algae abundant, discontinuous in the central part, apothecial cortex absent or invisible, colorless crystals absent; epihymenium yellow orange, K + purple; hymenium 70‒95 µm thick; hypothecium ca. 100 µm thick, prosoplectenchymatous, hyaline, some oil droplets present; paraphyses simple to slightly branched, 1–2 µm broad at base, with upper cell 2‒4 µm wide; CaOx crystals may present in the medulla of apothecia; asci 8-spored; ascospores hyaline, regularly ellipsoid, thin-walled, (10‒)12.6 ± 1.5(‒16) × (4‒)5.7 ± 0.8(‒8.0) µm, length/width ratio (2.1–)2.2 ± 0.1(‒2.2), ascospore septa (2‒)2.9 ± 0.3(‒4) µm, length/septum width ratio (4.3–)4.4 ± 0.1(‒4.4) (n = 39; N = 2). Pycnidia abundant, ostiole orange to red, immersed, almost invisible or indistinct, conidia ovoid or short bacilliform, (2.3‒)3.1 ± 0.4(‒4.4) (n = 301) × (0.8‒)1.2 ± 0.2(‒1.7) (n = 300) µm, length/width ratio (2.1–)2.5 ± 0.4(‒3.5) (N = 10).

*Distribution and ecology:* The species is reported mainly from Africa, with single records from the Arabian Peninsula and South America (Argentina and Uruguay) (Kärnefelt 1990; Wilk 2022). It grows on calcareous and siliceous rocks.

*Notes:* The lectotype of *W. ochraceofulva* is incomplete, the specimen is represented only by the central part of thallus, and it entirely lacks the thallus margin, which should produce marginal lobes. This was considered during the PBPB analyses, and the character concerning marginal lobes was coded as unknown (not as absent). The central part of the thallus in *W. ochraceofulva* is quite characteristic as it is areolate with small isidia at the areole margins. Those morphological features and anatomical ones observed in the lectotype, agree with the observations made on the additional material of the species.

The species may be confused with *W. variegata* (discussed above) and *W. brouardii*. For comparison with *W. brouardii*, see the discussion in Wilk (2022).

*Additional material examined:* Africa ‒ **Saudi Arabia:** Al Dalaghan area, Asir National Park, alt. 2170 m, high plain with piles of rocks, 15 Feb. 1982, *G. Zapletal s.n.* (E 905776, BM1247493, E905777). **Kenya:** Rift Valley Prov., Nakuru-Lake Nakuru, alt. 1750 m, on sandstone in the pasture, May 1949, *R. A. Maas Geesteranus 10,299 & L 4644a* (LD 1087200, LD1000623). **Lesotho:** Leribe, Buthabuthe, March 1963, *L. Kofler s.n.* (LD1066528, LD1066592); Kopje near Buthabuthe, on soft sandstone, 1963, *L. Kofler s.n.* (LD1024127). **Namibia:** Otjozondjupa Region, Waterberg Plateau National Park, primary forest with large Ficus at the base of Waterberg Plateau, alt. 1469 m, 15 June 2019, *A. Flakus 19/162* (KRAM, DUKE). **South Africa:** Natal, Vryheid Div., 5 miles N of Vryheid, on steep rock N of road, alt. 1300 m, 24 Oct. 1953, *O. Almborn 7976* (LD 1024896, LD 1024960); Ladybrand Div., alt. 1500–1800 m, on sandstone rock in broken, lightly wooded country, exposed, Nov. 1949, *R. A. Maas Geesteranus L 6546* (LD 1024063). South America ‒ **Argentina:** Prov. Jujuy, Santa Barbara, alt. 1300 m, 9 July 1901, *R.* *E. Fries 50* (LD 1076640, as *Callopisma subnitidum*). **Bolivia:** 28 km from Sucre, on the road to Aiguille, 2 May 1963, *D.* and *V. Ugent s.n.* (MIN 846514). **Uruguay:** Dept. Maldonado, Rio de la Plata, Isla Gorriti, alt. 0–1 m, 18 March 1984, *H. S. Osorio 8345* (MIN).

#### ***Wetmoreana subnitida*** (Malme) Wilk & Lücking, comb. nov. (Fig. 7h–m)

*MycoBank:* MB851738.

*Basionym: Callopisma subnitidum* Malme, Ark. Bot. 20A(9): 41 (1926). 

*Type:* **Argentina:** Argentina septentrionalis, Salta, c 50 m ö staden, 16 May 1901, *R. E. Fries 15* (S F128127, **lectotype designated here**, MBT10017684).

*Synonym: Caloplaca subnitida* (Malme) Zahlbr., Cat. Lich. Univ. 7: 267 (1931).

*Description:* Thallus crustose, areolate in centre, lobate at margin, orbicular to irregular in outline, 3.5‒5.0 cm wide, tightly attached to substratum, orange to reddish orange, K + purple, matte, only partly glossy, somewhere slightly white pruinose, pseudocyphellae and other cracks absent, areoles plane and polygonal, 0.2‒0.7 mm wide, marginal lobes plane, widened at the tips, 0.4‒1.8 mm long, 0.2‒1.0 mm wide at the tips, 130‒150 µm thick; vegetative propagules absent; prothallus absent; upper cortex 20‒50 µm thick, paraplectenchymatous, uneven, without distinct cortex cones, necral layer somewhere present, 2.5‒7 µm thick, colorless crystals absent; algal layer continuous; medulla without colorless crystals. Apothecia abundant, ± scattered, rounded, erumpent to sessile, zeorine, 0.2‒0.5 mm in diam.; disc first concave, then plane, ± concolorous with thallus, K + purple, epruinose, entire (without cracks); both margins distinguishable but inconspicuous, proper margin slightly prominent to level with disc, paler than disc, thalline margin partly to much reduced, even; parathecium 40‒70 µm thick, prosoplectenchymatous; amphithecium dominate, 70‒85 µm thick, algae abundant, forming a continuous layer, apothecial cortex 8.5‒14 µm thick, paraplectenchymatous, colorless crystals absent; epihymenium yellow orange, K + purple; hymenium ca. 80 µm thick; hypothecium ca. 50 µm thick, prosoplectenchymatous, hyaline, oil droplets absent; paraphyses simple, 1.5‒2 µm broad at base, with upper cell slightly wider, 3.5‒4 µm; asci 8-spored; ascospores hyaline, regularly ellipsoid, thin-walled, (8‒)9.6 ± 1.5(‒12) × (4‒)5.1 ± 0.9(‒7.0) µm, length/width ratio (1.9–)2.1 ± 0.3(‒2.3), ascospore septa (2‒)3.2 ± 0.8(‒5) µm, length/septum width ratio (3.0–)3.0 ± 0.1(‒3.1) (n = 14; N = 2). Pycnidia abundant, indistinct, conidia short bacilliform, (2.8‒)3.7 ± 0.3(‒4.2) (n = 70) × (0.8‒)1.1 ± 0.1(‒1.4) (n = 70) µm, length/width ratio (3.2–)3.3 ± 0.1(‒3.4) (N = 2).

*Distribution and ecology: Wetmoreana subnitida* is known from Argentina and Peru. It grows on siliceous rocks.

*Notes:* This species was described by Malme (1926) from South America (Argentina) under the name *Callopsima subnitidum*. The original material of this species is heterogeneous, including two different lobate species, i.e., one fertile and the second sterile forming isidia. Kärnefelt (1990) proposed lectotypification of *Callopisma subnitidum* based on the sterile specimen with isidia, and consequently synonymized this species with *Caloplaca ochraceofulva*. However, our investigation revealed that the original description of *Callopsima subnitidum* (Malme 1926) is clearly based on the fertile material producing apothecia. The description includes details about apothecia and ascospores but there is no mention of vegetative propagules. Furthermore, according to the protologue, the algal layer is more or less continuous, and the tips of marginal lobes are usually about 0.5 mm wide, rarely up to 1 mm. These features better fit the fertile specimen (*R.E. Fries 15*), because the sterile specimen (*R.E. Fries 50*) has a discontinuous algal layer where algae are arranged in distinct groups, and the marginal lobes are wider on average. Malme also highlighted the character of the ascospores of *Callopsima subnitidum* when comparing this species with another taxon, *Variospora aurantia* (= *Lecanora callopisma*). All this clearly shows that the lectotypification by Kärnefelt (1990) is not in accordance with the protologue and has to be superseded [ICNafp Art. 9.19(c)]. In doing so, an existing name becomes available for a taxon that would otherwise have to be described as new. Therefore, we superseed the previous lectotypification of *C. subnitidum* with the fertile specimen *R. E. Fries 15*, treating its combination into *Wetmoreana* as a taxon different from *W. ochraceofulva*. The sterile specimen originally given as a syntype of *C. subnitidum* (*R. E.* Fries 50, LD 1076640) is confirmed as representing *Wetmoreana ochraceofulva*. *Wetmoreana subnitida* differs from *W. ochraceofulva* mainly in anatomical features, in addition to the absence of vegetative propagules and a slightly white-pruinose thallus. *Wetmoreana subnitida* is further distinguished by the presence of a necral layer, a continuous algal layer, abundant apothecia, noticeably smaller ascospores (8–12 µm vs. 10–16 µm long) with slightly thicker septa (2–5 µm vs. 2–4 µm wide), a lower length/septum width ratio (3 vs. 4.3–4.4), and the absence of crystals in the thallus and apothecia.

*Wetmoreana subnitida* is situated within the *W. brouardii* complex. It differs from *W. brouardii* in having abundant apothecia, thicker ascospore septa (2‒5 µm vs. 2‒3.5 µm), and lacking vegetative propagules. *Wetmoreana subnitida* differs from *W. appressa* by having a reddish colored thallus, distinctly thinner ascospore septa (2–5 µm vs. 4.5–9 µm), and absence of colorless crystals in the thallus. *Wetmoreana subnitida* is distinguished from *W. bahiensis* by wider ascospores (4–7 µm vs. 4–5.5 µm), thicker ascospore septa (mean thickness 3.2 µm vs. 4.6 µm), and absence of crystals in the thallus (see Discussion section for more remarks). *Wetmoreana subnitida* may be confused with *W. intensa*, but the former differs from the latter in having longer marginal lobes (up to 1.8 mm vs. up to 1 mm), plane central areoles without any dark punctiform depressions, abundant apothecia and pycnidia (Aptroot et al. 2021).

The additional examined *W. subnitida* specimen collected by W. J. Eyerdam (26,062, F; PBPB no. 65) is somewhat problematic, as it shows some similarities to *W. bahiensis* as well. The PCA diagram has not resolved its affiliation, and *W. subnitida* (PBPB no. 65) is close to both the type of *W. subnitida* and *W. bahiensis* (Additional file 18: Fig. S11). However, *W. subnitida* (PBPB no. 65), like the lectotype of this species, lacks crystals luminescent under polarized light in both the medulla of thallus and apothecia. While in *W. bahiensis* such crystals are found in the whole medulla of the thallus (these are crystals of unknown origin, and some CaOx crystals). This chemical evidence supports the placement of the W. J. Eyerdam specimen within *W. subnitida*. However, considering some uncertainty, the description of *W. subnitida* is based only on type specimen.

*Additional material examined:* **Peru:** Dept. La Libertad, Prov. Trujillo, about 15 km south of Trujillo, on rock slides, lomas country, alt. 300 m, 2 Sept. 1938, *W. J. Eyerdam 26,062* (F, C1026062F).

#### ***Squamulea evolutior*** (Zahlbr.) Wilk & Lücking, comb. et stat. nov. (Fig. 13a–g)

**Fig. 13:**
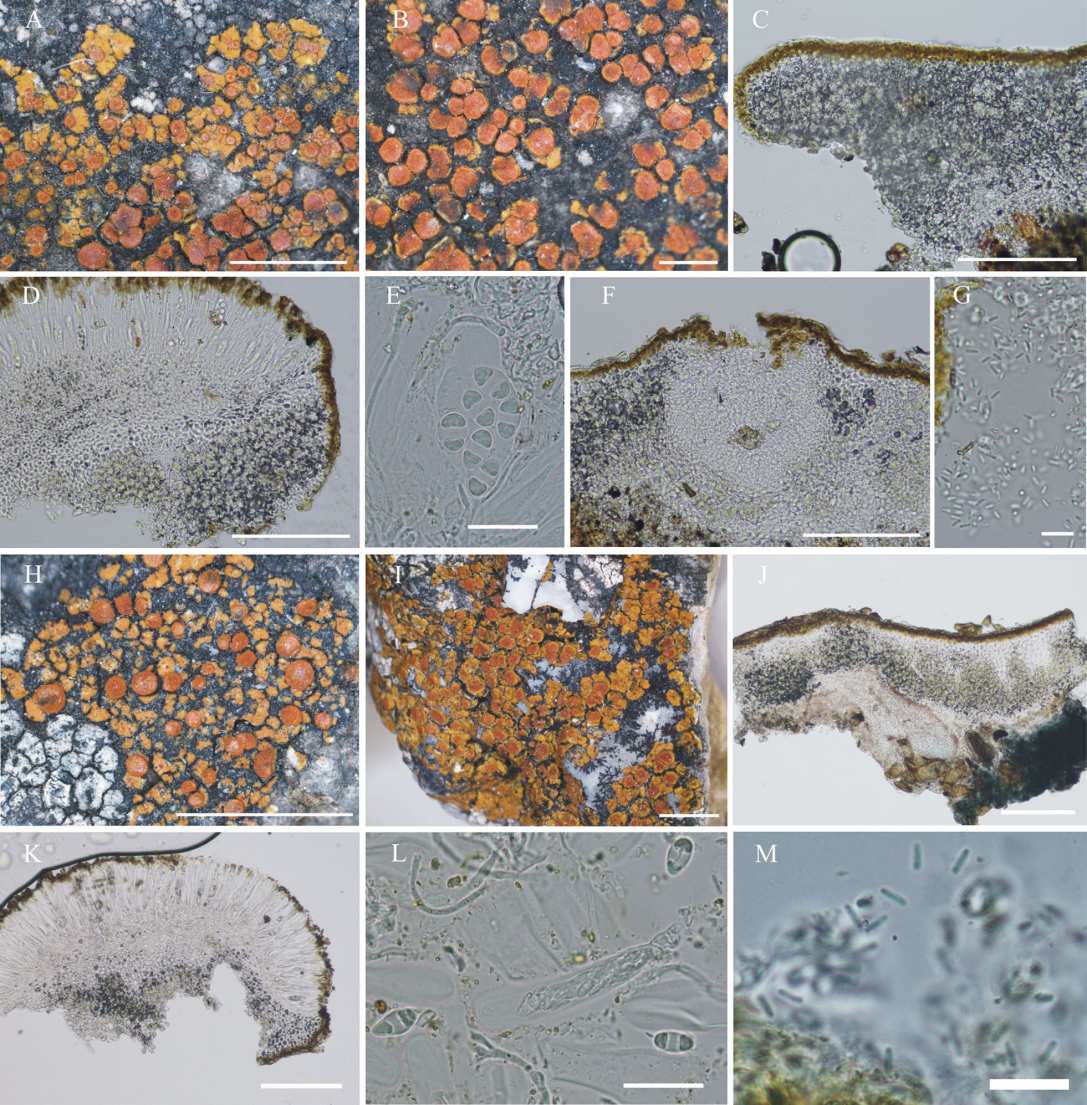
**A**–**G** *Squamulea evolutior* (holotype, **A** thallus habit, **B** close-up on apothecia, **C** section of thallus showing thin paraplectenchymatous upper cortex, **D** section of apothecium showing paraplectenchymatous parathecium, **E** ascus with spores, **F** section of pycnidium, **G** conidia). **H** *Caloplaca rubina* (lectotype, thallus habit). **I**–**M** *Squamulea muelleri* (lectotype, **I** thallus habit, **J** section of thallus, **K** section of apothecium showing paraplectenchymatous parathecium, **L** ascospores, **M** conidia). Scale in **A**, **H**, **I** = 2 mm, in **B** = 1 mm, in **C**, **D**, **F**, **J**, **K** = 100 µm, in **E**, **L** = 20 µm, in **G**, **M** = 10 µm

*MycoBank:* MB851739.

*Basionym: Caloplaca rubina* var. *evolutior* Zahlbr., Die Flechten der Juan-Fernandez-Inseln: 396 (1924).

*Type:* **Chile:** Juan Fernandez Islands, Masafuera, naturter[?] Teil von Quebrada de las Casas, 10 March 1917, *C. & I. Skottsberg s.n.* (UPS 204816!—holotype).

*Description:* Thallus squamulose, irregular in outline, loosely attached to substrate, orange, K + purple, epruinose, pseudocyphellae or other cracks absent, squamules 120‒170 µm thick, plane, margins crenate; vegetative propagules absent; prothallus present, black; upper cortex 15‒35 µm thick, clearly paraplectenchymateous, even, necral layer somewhere present, ca. 3.5 µm thick, colorless crystals absent; algal layer continuous; medulla paraplectenchymatous, colorless crystals absent. Apothecia abundant, crowded, mostly angular, sessile, clearly zeorine, 0.1‒0.8 mm in diam.; disc first concave, then plane to slightly convex in old apothecia, dark darker than thallus, K + purple, epruinose, entire; both margins distinguishable but inconspicuous, proper margin slightly prominent to level with disc, ± concolorous with disc, thalline margin much reduced, even; parathecium 35‒60 µm thick, paraplectenchymateous; amphithecium dominate, 60‒95 µm thick, algae abundant to scarce, continuous to discontinuous, apothecial cortex indistinct but forms clear and even layer, 17 µm thick, paraplectenchymateous, colorless crystals absent; epihymenium yellow orange or red, K + purple; hymenium 68‒85 µm thick; hypothecium 95–136 µm thick, paraplectenchymateous, hyaline, oil droplets absent; paraphyses simple or slightly branched, 1‒2 µm broad at base, with upper cell 2–5 µm wide, oil droplets absent; asci 8-spored; ascospores hyaline, widely ellipsoid, thin-walled, (8‒)11.0 ± 1.1(‒13) × (5‒)6.6 ± 0.7(‒8.0) µm, length/width ratio (1.6–)1.6 ± 0.1(‒1.7), ascospore septa (3‒)3.5 ± 0.6(‒5) µm, length/septum width ratio (2.8–)3.1 ± 0.4(‒3.4) (n = 51; N = 2). Pycnidia few, ostioles reddish, almost totally immersed, almost invisible, conidia short to long bacilliform, (2.9‒)3.4 ± 0.3(‒4.3) (n = 111) × (0.8‒)1.0 ± 0.1(‒1.2) (n = 97) µm, length/width ratio (3.2–)3.4 ± 0.3(‒3.6) (N = 2).

*Distribution and ecology: Squamulea evolutior* is known only from Chile, the Juan Fernandez Islands. It was collected on siliceous rocks.

*Notes: Squamulea evolutior* is placed within *Squamulea* with strong support in the PBPB analyses (ML, Fig. 2) as sister to *S. subsoluta*. The species fits very well with the morphology of *Squamulea*, and we proposed a new combination for it. Originally the taxon was described as *Caloplaca rubina* var. *evolutior* (Zahlbruckner 1924), but our study indicates that it is not conspecific with *C. rubina* s.str. (see below). *Squamulea evolutior* differs from *C. rubina* by having a well developed squamulose thallus, with clearly zeorine, larger apothecia (0.1–0.8 mm vs. 0.2–0.5 mm in diameter), and slightly larger ascospores (8–13 × 5–8 µm vs. 9–11 × 5–6 µm) (Zahlbrucner 1924). *Squamulea evolutior* is similar to *S. subsoluta* but differs from the latter in having a distinctly squamulose thallus and a much reduced thalline margin visible at the base of mature apothecia (see also Discussion). *Squamulea evolutior* may be confused with *W. sliwae*. Both species were discussed under the latter taxon.

*Additional material examined:* **Chile:** Juan Fernandez Islands, Masafuera, naturter[?] Teil von Quebrada de las Casas, 10 March 1917, *C. & I. Skottsberg s.n.* (UPS 204817, sub *Caloplaca rubina*).

#### ***Caloplaca rubina*** Zahlbr., Die Flechten der Juan-Fernandez-Inseln: 396 (1924) (Fig. 13h)

*Type:* **Chile:** Juan Fernandez Islands, Masatierra, niedr. Felsrücken am Südabhang v. Tres Puntas, St. 8, 5 Jan. 1917, *C. & I. Skottsberg s.n.* (S L2586, **lectotype selected here**, MBT10017689).

*Notes: Caloplaca rubina* is distinctive species producing reddish, tiny thalli consisting of dispersed, small and tightly adhered to the substrate areoles, surrounded by a black prothallus, and very small concolorous with thallus apothecia. It grows on volcanic rock. The possible affiliation of this species to *Squamulea* must be confirmed by molecular analysis or anatomical studies of the apothecial margin. The specimen collected by C. & I. Skottsberg (UPS 204817), labelled originally as *Caloplaca rubina*, appears to represent *Caloplaca rubina* var. *evolutior*. The latter produces rather well-developed squamulose thalli and is conspecific with the lectotype of *Squamulea evolutior*.

*Additional material examined:* **Chile:** Juan Fernandez Islands, Masatierra, narn Tres Puntas, 5 Jan. 1917, *C. & I. Skottsberg s.n.* (BM 001096590).

#### ***Squamulea muelleri*** (Vain.) Wilk & Lücking, comb. nov. (Fig. 13i–m)

*MycoBank:* MB851740.

*Basionym: Placodium muelleri* Vain. [as '*mülleri*'], Acta Soc. Fauna Flora fenn. 7: 120 (1890).

*Type:* **Brazil:** ad saxa granitica in littore maris prope Rio de Janeiro, 1885, *E. Wainio*, Lich. Brazil. Exs. 219 (M 0054898!—lectotype; see Wetmore, Bryologist 106: 155. 2003).

*Description:* Thallus squamulose, irregular in outline, tightly to loosely attached to substrate, pale to dark orange, K + purple, epruinose, matte, pseudocyphellae or other cracks absent, squamules, adpressed to stipitate in the crevices of rocks, plane to undulate, 110‒350 µm thick, margins crenate; vegetative propagules absent; prothallus present, distinct, black, dendroid; upper cortex 17‒43 µm thick, paraplectenchymateous, even to uneven, necral layer somewhere present, ca. 3.4 µm thick, colorless crystals absent; algal layer continuous; medulla thin, ca. 17 µm thick, indistinct, colorless crystals absent. Apothecia abundant, crowded, rounded to angular, erumpent to sessile, zeorine, 0.2‒0.7 mm in diam.; disc first concave, then plane also in old apothecia, darker than thallus, K + purple, epruinose, entire; both margins distinguishable but inconspicuous, proper margin prominent, paler than disc; thalline margin inconspicuous, partly to much reduced, even; parathecium 68‒102 µm thick, paraplectenchymateous; amphithecium ca. 95 µm thick, algae abundant, continuous to discontinuous, apothecial cortex almost invisible, ca. 8.5 µm thick, paraplectenchymateous, colorless crystals absent; epihymenium yellow brownish, K + purple; hymenium 85 µm thick; hypothecium 60–85 µm thick, paraplectenchymateous, hyaline, oil droplets absent; paraphyses simple to slightly branched, 1 µm broad at base, with upper cell 2–4 µm wide, oil droplets absent; asci 8-spored; ascospores hyaline, widely ellipsoid, thin-walled, (10‒)10.3 ± 0.6(‒12) × (5‒)5.6 ± 0.5(‒6.5) µm, length/width ratio 1.8, ascospore septa (3‒)3.9 ± 0.6(‒5) µm, length/septum width ratio 2.6 (n = 15; N = 1). Pycnidia few, ostioles orange, immersed, indistinct, conidia short bacilliform, (2.4‒)3.4 ± 0.4(‒4.2) (n = 61) × (0.7‒)1.0 ± 0.1(‒1.4) (n = 48) µm, length/width ratio 3.4 (N = 1).

*Notes: Squamulea muelleri* is a historically described species described by Wainio in 1890 from Brazil and has been known only from South America till present (e.g., Malme 1926; Osorio 1972; Spielmann 2006). However, the studied material of this species is not homogenous and requires further study to resolve its taxonomy. Nevertheless, the type material of *C. muelleri* used in this study clearly clustered with *Squamulea* based on PBPB analysis (only ML, BS = 100, Fig. 2). This affiliation was also confirmed by the morphological studies, which showed the presence of a paraplectenchymatous exciple of apothecia and thalline cortex, and similar, rather small ascospores characteristic for *Squamulea* spp. (Fig. 13i–m). *Caloplaca muelleri* is located in the *S. subsoluta* clade and was considered by us a synonym of the latter species since the morphology of both taxa is quite similar. However, examined type of *C. muelleri* has ascospores quite smaller than in *S. subsoluta* (10–12 × 5–6.5 µm vs. 9.5–15 × 5–9 µm), and such small ascospores are diagnostic for *C. muelleri* according to Wainio (1890) and Malme (1926). Therefore, we decided to treat this species as a separate taxon pending more study material and results of molecular studies providing additional evidence, especially since the entire *S. squamulosa/subsoluta* group is still largely unresolved taxonomically, and the species *S. subsoluta*, appears to include many cryptic taxa (Bungartz et al. 2021).

The additional examined specimens of *C. muelleri* collected by Lorentz (KRAM) [treated as *C. muelleri* by Malme (1926)] and *H. S. Osorio 2342* (F), both from Uruguay, have similar ascospores to the type of *C. muelleri*, but superficially they are rather similar to *S. evolutior* producing distinctly squamulose and slightly stipitate thalli. The *C. muelleri* taxonomic description was based on an isolectotype, because the studied material of *C. muelleri* appears to be not homogenous.

*Additional material examined:* **Uruguay:** An sandsteinfelsen bei Concepcion am La Plata, 1877, *P. G. Lorentz s.n.* (Arnold, Lichenes Exssiccati 750, sub *Callopisma aurantiacum*) (KRAM L 3770). Maldonado-Arroyo Pan de Azucar-Paso Real, sur pierres dans une prairie pres le riusseau, 19 Feb. 1950, *H. S. Osorio 2342* (F).

## Taxon with unresolved taxonomy

### ***Wetmoreana ‘appressa’*** auct. non (Wetmore & Kärnefelt) Arup et al. (Fig. 14h–r).

**Fig. 14:**
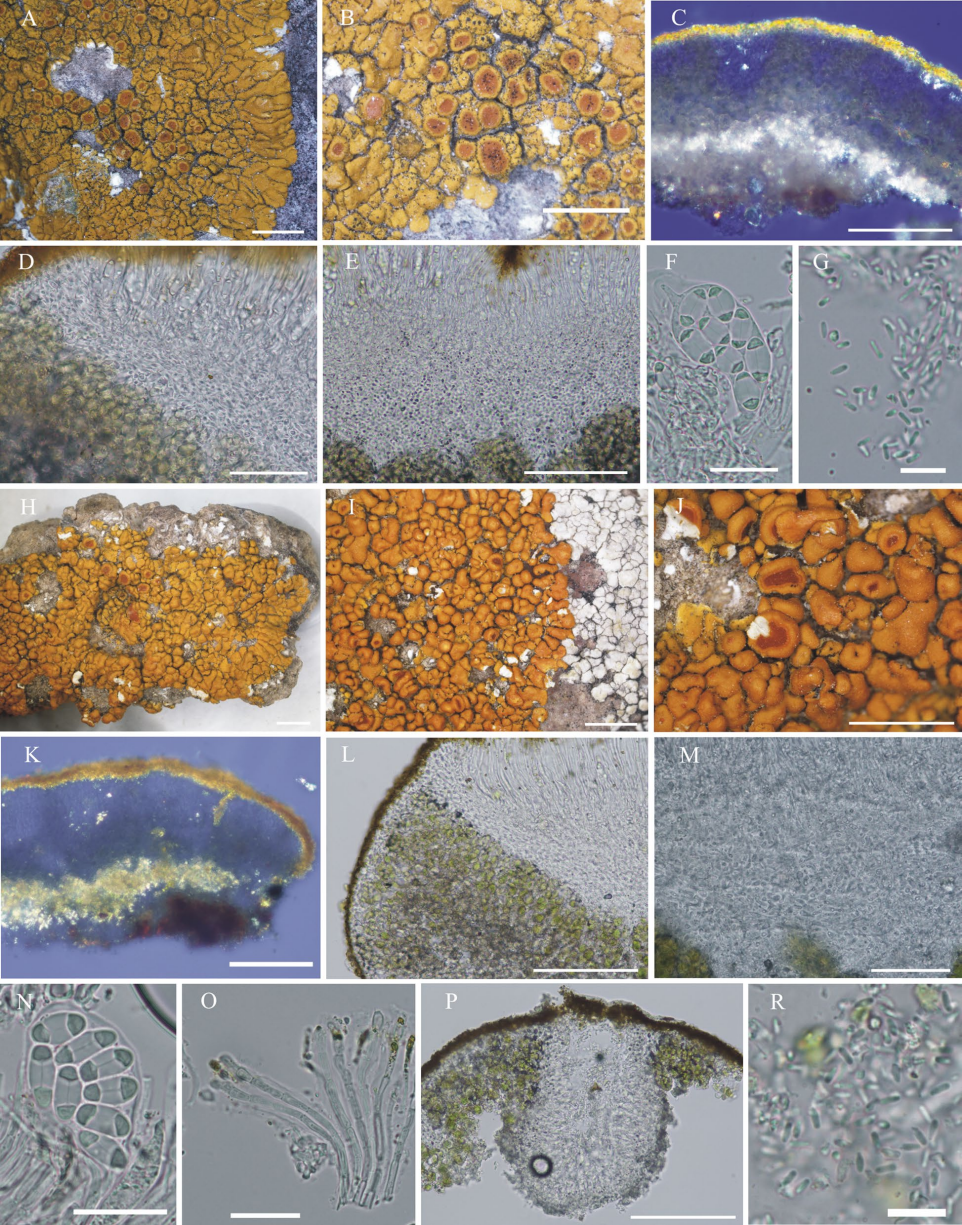
*Wetmoreana appressa* morphodeme. **A**–**G** *W. appressa* (holotype, **A** thallus habit, **B** close-up on apothecia, **C** section of thallus showing calcium oxalate crystals below algal layer prominent in polarized light,** D** section of apothecium showing prosoplectenchymatous parathecium, **E** section of apothecium showing hypothecium consisting of small rounded cells, **F** ascus with spores,** G** conidia), **H**–**R** *W. “appressa”* (*Wetmore 70,012*, LD 1084897, **H** thallus habit, **I** close-up on bullate thallus centre, **J** close-up on apothecia, **K** section of thallus showing medulla filled with calcium oxalate crystals prominent in polarized light, **L** section of apothecium showing prosoplectenchymatous parathecium, **M** section of apothecium showing prosoplectenchymatous hypothecium, **N** ascus with spores, **O** simple paraphyses, **P** section of pycnidium, **R** conidia. Scale in **A**, **B**, **H**, **I**, **J** = 2 mm, in** D**, **E**, **K**, **L**, **M**, **P** = 100 µm, in **F**, **N**, **O** = 10 µm, in **G**, **R** = 10 µm

*Description:* Thallus crustose, lobate, areolate in centre, irregular in outline, tightly to loosely attached to substrate, dark orange, K + purple, epruinose, smooth, pseudocyphellae and other cracks absent, areoles strongly convex, polygonal do granular, 0.2‒1.0 mm wide, marginal lobes plane to convex, widened at the tips, 0.5‒1.9 mm long, 0.3‒1.2 mm wide at the tips, 190‒220 µm thick; vegetative propagules absent; prothallus absent; upper cortex 20‒50 µm thick, paraplectenchymatous, uneven, necral layer somewhere present, 3.5‒15 µm thick, colorless crystals absent; algal layer continuous to discontinuous; medulla filled with CaOx crystals. Apothecia scarce, scattered, rounded to flexuous, erumpent to sessile, zeorine, 0.3‒0.9 mm in diam.; disc first concave, then plane, darker than thallus, K + purple, entire (not cracked), epruinose; both margins distinguishable but inconspicuous, apothecial margin swollen, proper margin very thin, prominent, paler than disc, thalline margin ± persistent to partly reduced, even; parathecium 68‒102 µm thick, prosoplectenchymatous, consisting of radiating hyphae, in upper part ovoid cells 6.8‒8.5 × 3.4‒4.3 µm; amphithecium dominate, 140‒250 µm thick, algae abundant, discontinuous, apothecial cortex very thin, indistinct, 8.5‒34 µm thick, paraplectenchymatous; epihymenium yellow brown, K + purple; hymenium ca. 102 µm thick; hypothecium ca. 128 µm thick, prosoplectenchymatous, hyaline, oil droplets present; paraphyses simple, 2 µm broad, with upper cell no or slightly wider, 2‒3 µm, CaOx crystals present in the medulla of apothecia; asci 8-spored; ascospores hyaline, regularly ellipsoid, thin-walled, (12‒)13.4 ± 1.1(‒15.5) × (5.5‒)6.3 ± 0.7(‒7.0) µm, length/width ratio 2.1, ascospore septa (5‒)5.7 ± 0.9(‒7.5) µm, length/septum width ratio 2.4 (n = 8; N = 1). Pycnidia abundant, ostiole red, distinct, partly immersed, conidia short bacilliform, (3.1‒)3.5 ± 0.3(‒4.4) (n = 24) × (0.8‒)1.1 ± 0.1(‒1.4) (n = 32) µm, length/width ratio 3.2 (N = 1).

*Notes:* *Wetmoreana ‘appressa’* is characterized by bullate central areoles, rather short marginal lobes, closely or loosely adherent to the substrate, and apothecia with distinctly swollen margins (Fig. 14a–g).

The sequenced specimen of *Wetmoreana ‘appressa’* (GenBank No. KC179332) differs phenotypically from the holotype of *W. appressa*, for which DNA sequences are unavailable. The two taxa are also located in different clades in our PBPB analyses (Fig. 2). These results indicate that the two species are not conspecific. The high phenotypic varability of *W. appressa* was also discussed by the author of the species (Wetmore and Kärnefelt 1998). Therefore, this species, *W. appressa* s. lat., requires further investigation, including molecular data, to see if the observed variability has support at the molecular level.

The holotype of *W. appressa* differs from the sequenced specimen of *W. ‘appressa’* in its pronounced close adherence to the substrate, plane to slightly convex central areoles, slightly longer and narrower marginal lobes (0.4–2.2 mm long, 0.2–0.9 mm wide vs. 0.5–1.9 mm long, 0.3–1.2 mm wide), apothecia with rather thin margin, slightly shorter ascospores [(10‒)12.6(‒15) µm vs. (12‒)13.4(‒15.5) µm] and thicker septum [(4.5‒)7.3(‒9) µm vs. (5‒)5.7(‒7.5) µm], and indistinct pycnidia.

*Material examined:* **Mexico:** Sonora, San Carlos, north of Guaymas, on a steep north slope above sheltered bay, alt. 20–30 m, 27°57′N, 111°03′30′′W, 16 March 1992, *C. Wetmore 70,012* (LD; GenBank no KC179332).

## Conclusion

Our study provides a first attempt at an integrative taxonomic approach to solve the delimitation of the genus *Wetmoreana* and its species. PBPB is so far the only approach known that allows to obtain predictive placements of specimens for which no sequence data are available and which provides a measure of statistical confidence for such placements. The obtained solutions are therefore testable, in that way differing from ad hoc taxonomy which weights individual characters subjectively in the decision-making process. The *Wetmoreana* case provides a good case study, as the results would not have been expected using a traditional ad hoc approach. A shortcoming of this method is that it relies on existing molecular reference trees, and the possibility that some query taxa for which DNA is unavailable may represent genera not yet known to science cannot be excluded. Also, the morphological taxon sampling is not always complete and here we might have missed some additional, similar and poorly understood South American taxa. On the other hand, this approach helps to precisely identify those taxa that require targeted sampling for molecular data, and additional phenotypes can be easily added later to undertake a broader analysis. As such, we consider our results as a testable hypothesis that serves as reference for further, more detailed studies in this group. An obvious challenge to this approach is the time-consuming assessment of a large number of characters and their assembly in a comprehensive data matrix. However, once assembled, the matrix allows a reproducible approach and opens avenues to various downstream analyses regarding character evolution.

### Supplementary Information

**Additional file 1: Table S1.** The taxa used in the phylogenetic and PBPB studies. The taxa for which the phenotype dataset was gathered are marked by a unique PBPB number (column B). Query specimens (column C) are those for which the sequences are not available. The newly proposed names for the selected studied taxa are included in the column D. Information about the type material is included in column E. For the taxa used in the phylogenetic analyses, the GenBank numbers of sequences are included and the newly obtained sequences are in boldface.

**Additional file 2: Table S2.** Continuous and discrete characters used in the analyses and their character state definitions and coding. The values of the weighted characters calculated by using ML and MP analyses in RAxML are included. Continuous characters are in boldface.

**Additional file 3: File S1.** Alignment of the ITS sequences of representatives of the genus *Wetmoreana* analysed in this study.

**Additional file 4: File S2.** Alignment of the nuLSU sequences of representatives of the genus *Wetmoreana* analysed in this study.

**Additional file 5: File S3.** Alignment of the mtSSU sequences of representatives of the genus *Wetmoreana* analysed in this study.

**Additional file 6: File S4.** Alignment of the ITS sequences of representatives of the genus *Wetmoreana* and morphologically similar members of the genera *Aridoplaca*, *Calogaya*, *Cinnabaria*, *Gyalolechia*, *Squamulea*, *Teuvoahtiana* and taxa *Caloplaca fernandeziana* included in the phenotype-based phylogenetic binning (PBPB) analyses.

**Additional file 7: Figure S1.** Cartoon tree showing placements of 73 queried *Teloschistaceae* specimens for which the DNA sequences are unknown, using 73 phenotypic traits for 112 specimens in total, based on PBPB and MP weighting technique. Numbers in square brackets indicate node numbers and numbers after the query name indicate bootstrap support for a particular placement of the query taxon based on its morphological features.

**Additional file 8: Figure S2.** Phylogenetic tree of *Wetmoreana* based on three loci (ITS, nuLSU and mtSSU) performed in RAxML. The support values associated with branches indicate maximum likelihood bootstrap values. The values ≥ 70 are considered as significant support in this study. The thickness of the branches corresponds to the level of support. The newly proposed names are signed. The numbers after species names corresponds to PBPB numbers.

**Additional file 9: Figure S3.** Phylogenetic tree of *Wetmoreana* based on ITS performed in RAxML.

**Additional file 10: Figure S4.** Phylogenetic tree of *Wetmoreana* based on nuLSU performed in RAxML.

**Additional file 11: Figure S5.** Phylogenetic tree of *Wetmoreana* based on mtSSU performed in RAxML.

**Additional file 12: Table S3.** PBPB results presented as classification table. Placements of query specimens according ML and MP weighting, with bootstrap support (BS) indicated.

**Additional file 13: Figure S6.** PCA ordination based on 63 phenotypic characters of 14 species of *Wetmoreana* including the binned taxa from the PBPB analysis.

**Additional file 14: Figure S7.** PCA ordination based on 46 phenotypic characters of the *Wetmoreana ochraceofulva* clade defined by PBPB-MP.

**Additional file 15: Figure S8.** PCA ordination based on 32 phenotypic characters of the *Squamulea subsoluta* clade defined by PBPB-ML.

**Additional file 16: Figure S9.** PCA ordination based on 36 phenotypic characters of the *Wetmoreana sliwae* clade defined by PBPB-ML.

**Additional file 17: Figure S10.** The comparison between the *ochraceofulva* /*variegata* and *sliwae* /*subparviloba* /*brachyloba* complexes using MRPP based on PBPB-MP (A, B) or molecular (C, D) defined clades, and for complete (A, C) and incomplete (without apothecia, B, D) datasets. The analyzed groups are as follow in case of PBPB-MP: GR01: *ochraceofulva* s.lat. vs. *sliwae* s.lat., GR02: *ochraceofulva* s.lat. vs. *sliwae* s.lat./*brachyloba*, GR03: *ochraceofulva* s.lat. vs. *sliwae*/*subparviloba*/*brachyloba*, GR04: *ochraceofulva*/*variegata* vs. *sliwae* s.lat., GR05: *ochraceofulva*/*variegata* vs. *sliwae* s.lat./*brachyloba*, GR06: *ochraceofulva*/*variegata* vs. *sliwae*/*subparviloba*/*brachyloba*. In case of DNA: GR01: *ochraceofulva* s.lat. vs. *sliwae* s.lat., GR02: *ochraceofulva* s.lat. vs. *sliwae*/*subparviloba*, GR03: *ochraceofulva*/*variegata* vs. *sliwae* s.lat., GR04: *ochraceofulva*/*variegata* vs. *sliwae*/*subparviloba*.

**Additional file 18: Figure S11.** PCA ordination of the *Wetmoreana brouardii* complex based on 34 phenotypic characters of 11 species.

## Data Availability

DNA sequences (newly generated for two specimens of *W. ochraceofulva*) are deposited in GenBank (https://www.ncbi.nlm.nih.gov/genbank/). Alignments of sequences used in this study are included as supplementary files (Additional files 3–6: Files S1–S4). Information about herbarium materials is included in Additional file 1: Table S1.

## Abbreviations

*A* value: Chance-corrected within-group agreement value
AA: Acetic acid
CaOx: Calcium oxalate
MRPP: Multi-response Permutation Procedure
PBPB: Phenotype-based Phylogenetic Binning
PCA: Principal Component Analysis
EPA: Evolutionary Placement Alogarithm

## Declarations

### Adherence to national and international regulations

The KW field survey and the resulting lichen collection were conducted with respect for the laws of the country of origin of the specimens.
