# Supplementary material for: Quantitative integrative taxonomy informs species delimitation in Teloschistaceae (lichenized Ascomycota): the genus Wetmoreana as a case study
Source: IMA Fungus. 2024 Apr 1;15:9. doi: 10.1186/s43008-024-00140-1 (PMC11225190; doi:10.1186/s43008-024-00140-1)
Supplement: Supplementary file 2 — Additional file 2: Table S2. Continuous and discrete characters used in the analyses and their character state definitions and coding. The values of the weighted characters calculated by using ML and MP analyses in RAxML are included. Continuous characters are in boldface. [file 43008_2024_140_MOESM2_ESM.docx]

Additional file 2: Table S2. Continuous and discrete characters used in the analyses and their character state definitions and coding. The values of the weighted characters calculated by using ML and MP analyses in RAxML are included. Continuous characters are in boldface.

| **Characters** | **ML** | **MP** | **State 0/?** | **State 1** | **State 2** | **State 3** | **State 4** | **State 5** |
| --- | --- | --- | --- | --- | --- | --- | --- | --- |
| Substrate (rock) | 41 | 80 | HCl– | HCl± | HCl+ |  |  |  |
| Thallus lobate | 100 | 67 | Absent | Present |  |  |  |  |
| Thallus sublobate | 94 | 93 | Absent | Present |  |  |  |  |
| Thallus areolate | 100 | 100 | Absent | Present |  |  |  |  |
| Thallus squamulose | 100 | 93 | Absent | Present |  |  |  |  |
| Thallus peltate | 100 | 100 | Absent | Present |  |  |  |  |
| Thallus areolate-squamulose (=subsquamulose) | 100 | 100 | Absent | Present |  |  |  |  |
| Thallus shape | 100 | 27 | Orbicular | Orbicular to irregular | Irregular | Unknown (*W. ochraceofulva* 48) |  | |
| Thallus attachment | 100 | 53 | Tightly | Tightly to loosely | Loosely |  |  |  |
| Thallus pruinosity | 100 | 73 | Epruinose | Partly pruinose | Totally pruinose |  |  |  |
| Thallus pseudocyphellae/other cracks | 84 | 27 | Absent | Inconspicious | Conspicious |  |  |  |
| Thallus areoles | 100 | 87 | Absent | Present |  |  |  |  |
| Thallus squamules | 100 | 87 | Absent | Present |  |  |  |  |
| Areoles/squamules surface | 100 | 27 | Strongly reduced | Plane | Plane to convex | Convex | Undulate/verruculose |  |
| Areoles shape | 100 | 67 | Absent | Polygonal | Polygonal to granular | Sublobate |  |  |
| Squamule margins lobate | 100 | 100 | Absent | Present |  |  |  |  |
| Squamule margins crenate | 100 | 80 | Absent | Present |  |  |  |  |
| Squamule margins entire | 100 | 100 | Absent | Present |  |  |  |  |
| Squamule margins lobulate | 100 | 93 | Absent | Present |  |  |  |  |
| Squamule spatial growth | 100 | 87 | Absent | Horizontally | Horizontally to irregularly | Irregularly |  |  |
| Marginal lobes/sublobes surface | 100 | 47 | Absent | Plane | Plane to convex | Convex | Unknown (*W. ochraceofulva* 48) |  |
| **Marginal lobes/sublobes length** | 100 | 40 | Absent | To 1 mm | To 2 mm | Over 2 mm | Unknown (*W. ochraceofulva* 48) |  |
| **Marginal lobes/sublobes width** | 100 | 27 | Absent | To 0.5 mm | To 1.0 mm | Over 1.0 mm | Unknown (*W. ochraceofulva* 48) |  |
| Vegetative propagules: soredia | 100 | 100 | Absent | Present |  |  |  |  |
| Vegetative propagules: isidia | 83 | 100 | Absent | Present |  |  |  |  |
| Vegetative propagules: schizidia | 85 | 80 | Absent | Present |  |  |  |  |
| Vegetative propagules: papillae | 100 | 100 | Absent | Present |  |  |  |  |
| Vegetative propagules locality: marginal | 97 | 80 | Absent | Present |  |  |  |  |
| Vegetative propagules: laminal | 39 | 87 | Absent | Present |  |  |  |  |
| Vegetative propagules: dissolved | 100 | 80 | Absent | Present |  |  |  |  |
| Vegetative propagules: under areoles | 4 | 93 | Absent | Present |  |  |  |  |
| Vegetative propagules: terminal | 100 | 100 | Absent | Present |  |  |  |  |
| Prothallus black | 97 | 93 | Absent | Present |  |  |  |  |
| Prothallus yellow | 100 | 100 | Absent | Present |  |  |  |  |
| Thallus necral layer | 100 | 47 | Absent | Somewhere present | Clearly present |  |  |  |
| Thallus algal layer | 100 | 53 | Continuous | Continuous to discontinuous | In distinct columnes/groups | Irregular |  |  |
| Apothecia frequency | 97 | 67 | Unknown | Few | Scarce | Abundant |  |  |
| Apothecia emergence | 100 | 67 | Unknown | Sessile | Sessile to erumpent | Erumpent | Immersed |  |
| Apothecia shape | 62 | 47 | Unknown | Round | Round to angular | Mostly angular | Angular to flexuous |  |
| Apothecia zeorine | 100 | 100 | Unknown | Absent | Present |  |  |  |
| Apothecia biatorine/pseudolecanorine | 100 | 100 | Unknown | Absent | Present |  |  |  |
| **Apothecia max. diameter** | 72 | 53 | Unknown | To 0.6 mm | To 0.9 mm | To 1.2 mm | Over 1.2 mm |  |
| Apothecia disc surface (old) | 91 | 47 | Unknown | Plane | Slightly convex |  |  |  |
| Apothecia disc color | 100 | 73 | Unknown | ±Concolorous with thallus | Darker than thallus | Contrasting against thallus |  |  |
| Apothecia disc pruinosity | 100 | 100 | Unknown | Epruinose | Pruinose |  |  |  |
| Apothecia disc surface continuity | 56 | 67 | Unknown | Entire | Entire to cracked | Cracked |  |  |
| Apothecia both margins visibility | 100 | 53 | Unknown | Not or almost not | Distinguishable but inconspiciuous | Distinguishable & conspicuous |  |  |
| Apothecial margin elevation | 95 | 40 | Unknown | Prominent | Slightly prominent to level with disc | Level with disc |  |  |
| Apothecia proper margin/margin color | 43 | 67 | Unknown | Paler than disc | ±Concolorous with disc | Concolorous with disc |  |  |
| Apothecia thalline margin formation (mature) | 100 | 53 | Unknown | ±Persistant to partly reduced | Partly reduced | Partly to much reduced | Much reduced |  |
| **Parathecium width** | 53 | 60 | Unknown | To 100 µm | Over 100 µm |  |  |  |
| Parathecium structure | 100 | 100 | Unknown | Prosoplectenchymatous | Paraplectenchymatous |  |  |  |
| **Amphithecium width** | 0 | 80 | Unknown | To 100 µm | Over 100 µm |  |  |  |
| **Parathecium/amphithecium ratio** | 89 | 73 | Unknown | P/A>1.1 | P/A=0.9 to 1.1 | P/A<0.9 |  |  |
| **Acospore lenght (L) mean value** | 100 | 60 | Unknown | To 13 µm | To 15 µm | Over 15 µm |  |  |
| **Acospore width (W) mean value** | 42 | 67 | Unknown | To 5 µm | To 7 µm | Over 7 µm |  |  |
| **Ascospore L/W ratio** | 97 | 73 | Unknown | To 1.8 times | To 2.5 times | Over 2.5 times |  |  |
| **Acospore septum thickness mean value** | 45 | 53 | Unknown | To 3 µm | To 5 µm | To 7 µm | Over 7 µm |  |
| **Ascospore L/septum ratio** | 49 | 47 | Unknown | To 2 times | To 3 times | To 4 times | To 5 times | Over 5 times |
| Pyknidia frequency | 12 | 0 | Unknown | Uncountable | Few | Abundant |  |  |
| Pyknidia visibility | 95 | 7 | Unknown | Invisible | Almost invisible | Indistinct | Distinct |  |
| **Conidia lenght mean value** | 42 | 73 | Unknown | To 3 µm | To 4 µm | Over 4 µm |  |  |
| **Conidia width mean value** | 45 | 60 | Unknown | To 1 µm | Over 1 µm |  |  |  |
| **Conidia L/W ratio** | 100 | 20 | Unknown | To 2.5 µm | To 3.5 µm | Over 3.5 µm |  |  |
| Conidia shape: ovoid | 100 | 67 | Unknown | Absent | Present |  |  |  |
| Conidia shape: bacilliform | 100 | 73 | Unknown | Absent | Present |  |  |  |
| Conidia shape: biguttulate | 100 | 100 | Unknown | Absent | Present |  |  |  |
| Thallus medulla CaOx crystals | 100 | 60 | Absent | Thin, distinct layer | Whole medulla (distinct layer absent) | |  |  |
| Thallus cortex CaOx crystals | 96 | 87 | Absent | Present |  |  |  |  |
| Apothecia CaOx crystals in cortex | 49 | 87 | Absent | Present |  |  |  |  |
| Apothecia CaOx crystals in algal layer | 48 | 93 | Absent | Present |  |  |  |  |
| Apothecia CaOx crystals in parathecium | 94 | 93 | Absent | Present |  |  |  |  |
| Apothecia CaOx crystals in basal part | 100 | 73 | Absent | Present |  |  |  |  |
