# Supplementary figures and images for: Quantitative integrative taxonomy informs species delimitation in Teloschistaceae (lichenized Ascomycota): the genus Wetmoreana as a case study
Source: IMA Fungus. 2024 Apr 1;15:9. doi: 10.1186/s43008-024-00140-1 (PMC11225190; doi:10.1186/s43008-024-00140-1)

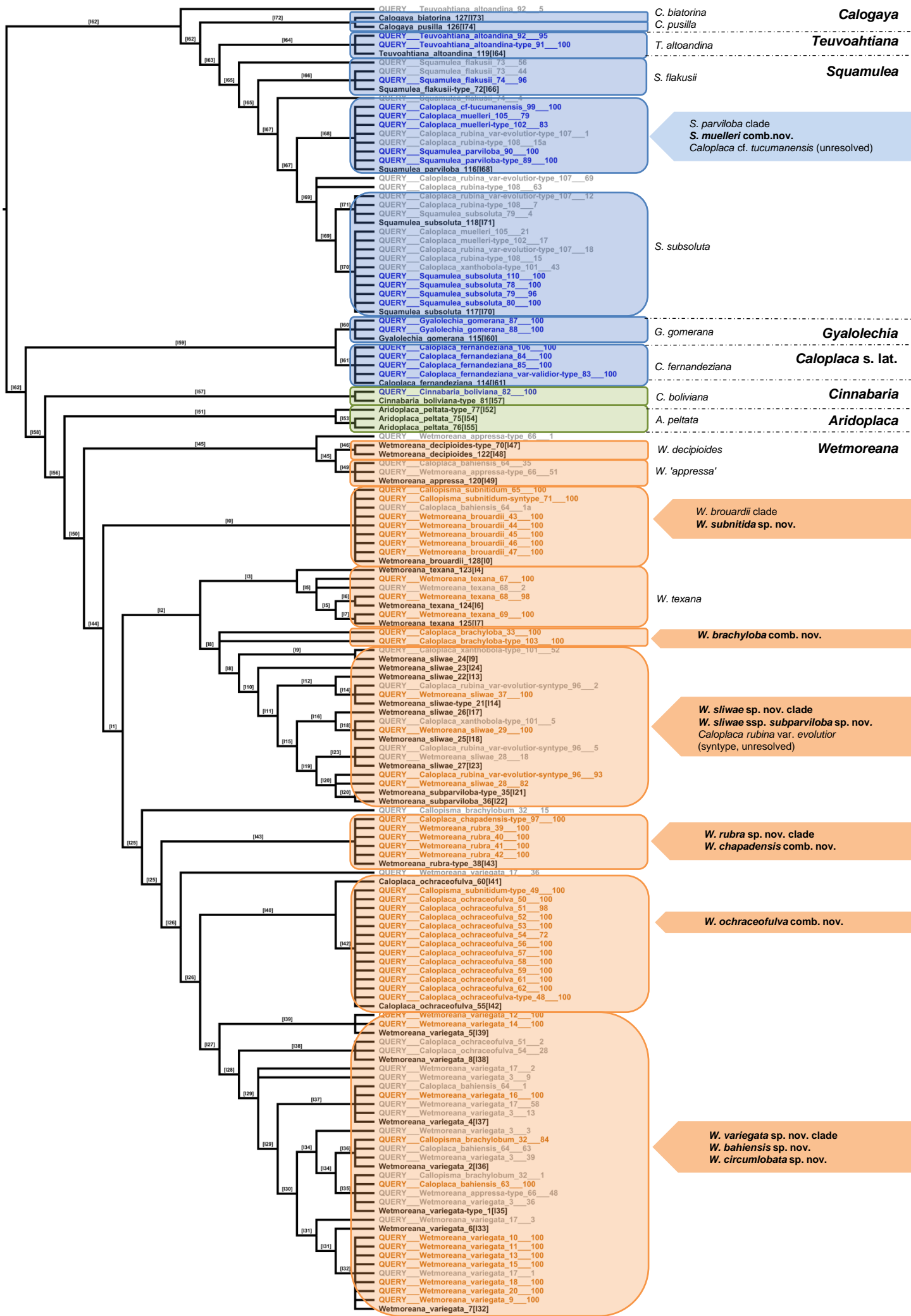

Supplement: Supplementary file 7 — Additional file 7: Figure S1. Cartoon tree showing placements of 73 queried Teloschistaceae specimens for which the DNA sequences are unknown, using 73 phenotypic traits for 112 specimens in total, based on PBPB and MP weighting technique. Numbers in square brackets indicate node numbers and numbers after the query name indicate bootstrap support for a particular placement of the query taxon based on its morphological features. [file 43008_2024_140_MOESM7_ESM.pdf]

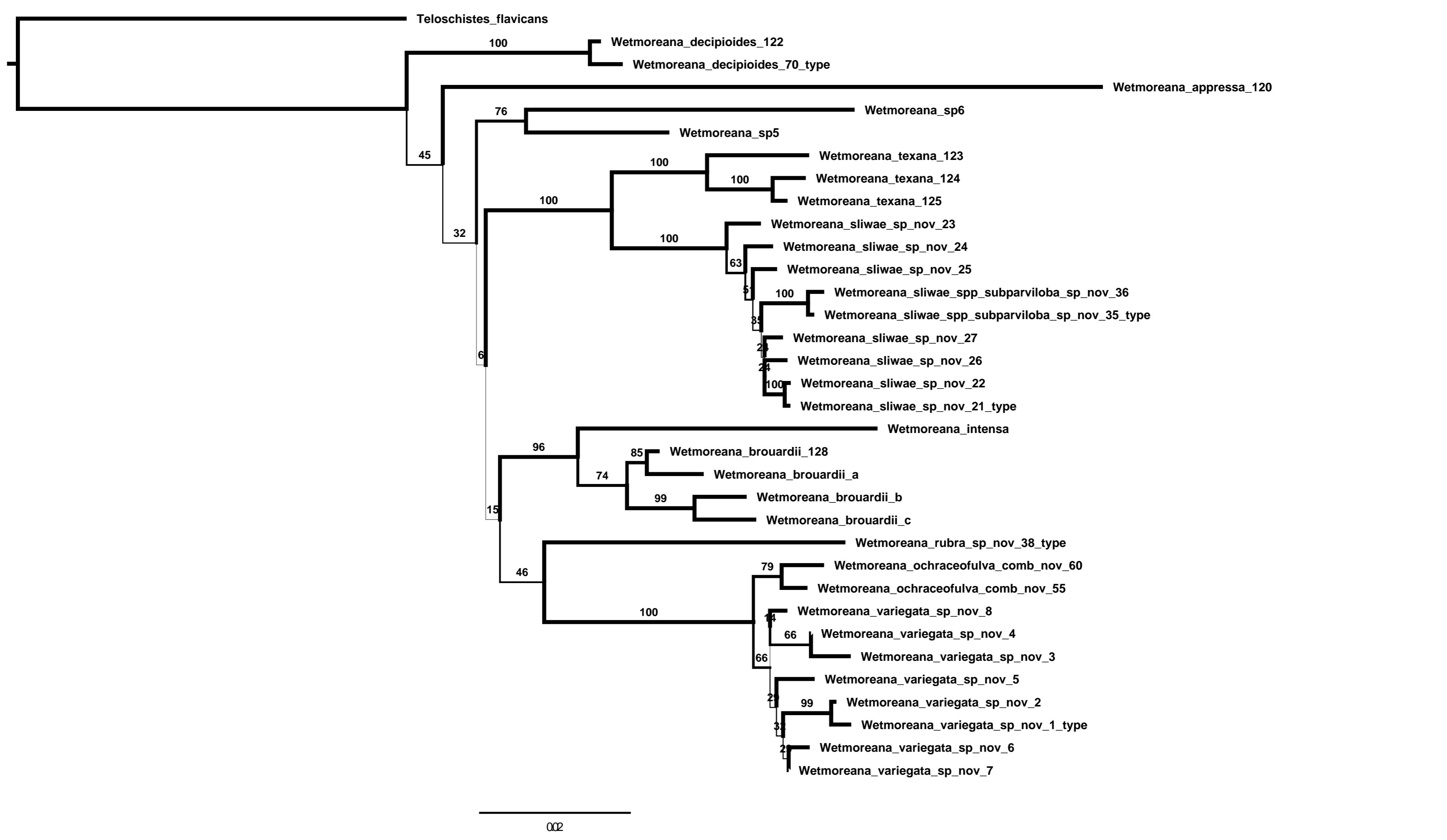

Supplement: Supplementary file 8 — Additional file 8: Figure S2. Phylogenetic tree of Wetmoreana based on three loci (ITS, nuLSU and mtSSU) performed in RAxML. The support values associated with branches indicate maximum likelihood bootstrap values. The values ≥ 70 are considered as significant support in this study. The thickness of the branches corresponds to the level of support. The newly proposed names are signed. The numbers after species names corresponds to PBPB numbers. [file 43008_2024_140_MOESM8_ESM.pdf]

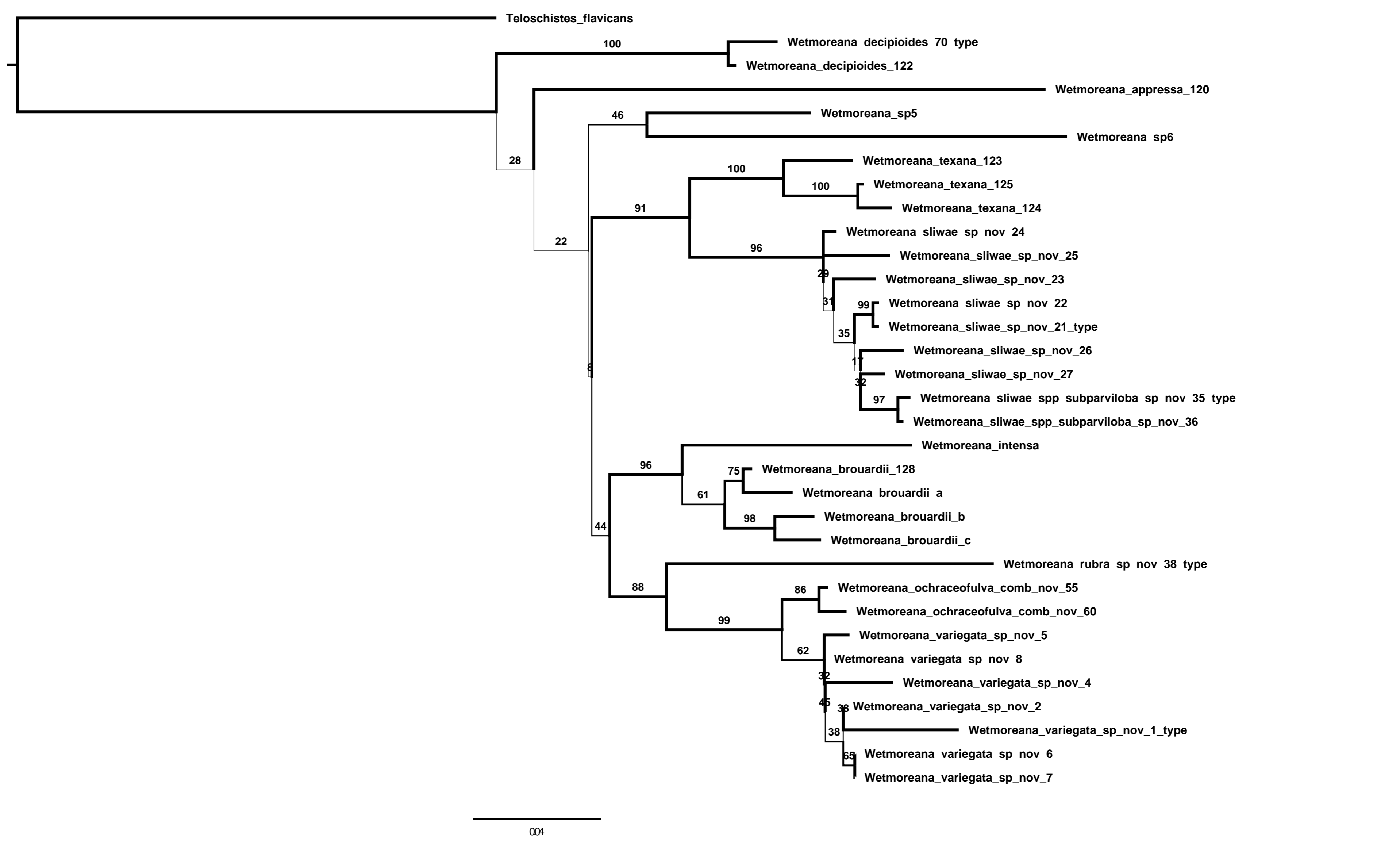

Supplement: Supplementary file 9 — Additional file 9: Figure S3. Phylogenetic tree of Wetmoreana based on ITS performed in RAxML. [file 43008_2024_140_MOESM9_ESM.pdf]

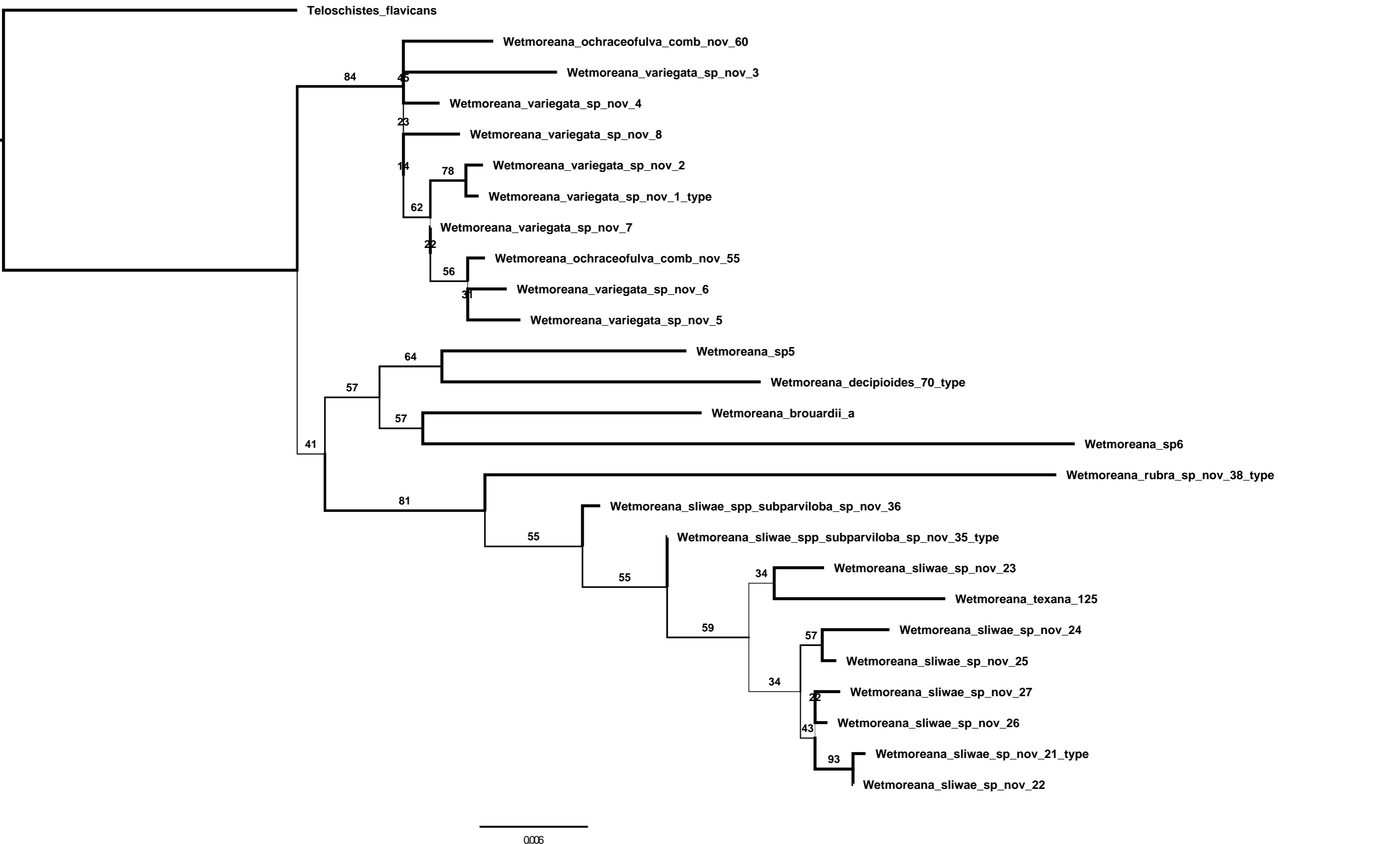

Supplement: Supplementary file 10 — Additional file 10: Figure S4. Phylogenetic tree of Wetmoreana based on nuLSU performed in RAxML. [file 43008_2024_140_MOESM10_ESM.pdf]

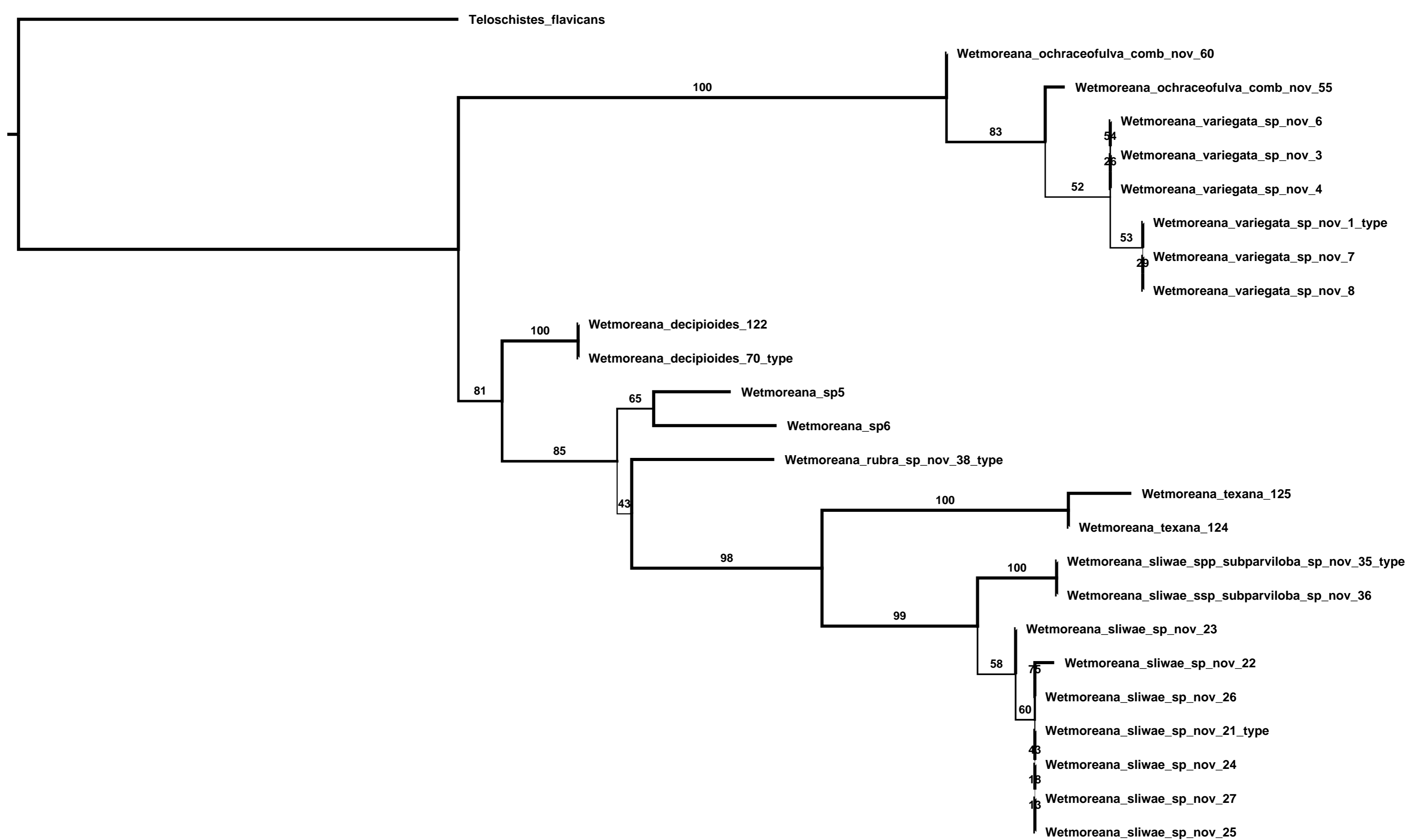

Supplement: Supplementary file 11 — Additional file 11: Figure S5. Phylogenetic tree of Wetmoreana based on mtSSU performed in RAxML. [file 43008_2024_140_MOESM11_ESM.pdf]

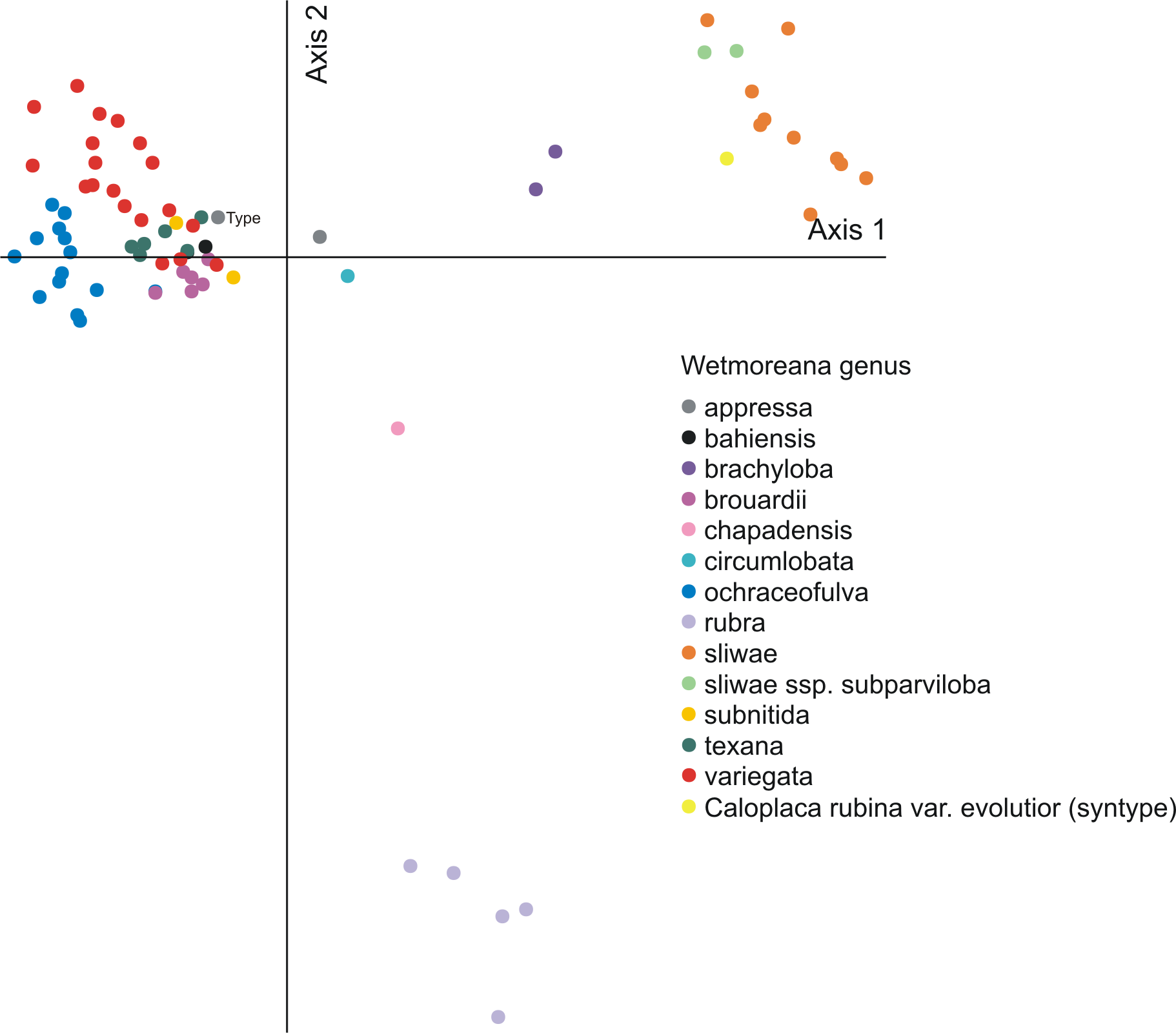

Supplement: Supplementary file 13 — Additional file 13: Figure S6. PCA ordination based on 63 phenotypic characters of 14 species of Wetmoreana including the binned taxa from the PBPB analysis. [file 43008_2024_140_MOESM13_ESM.tif]

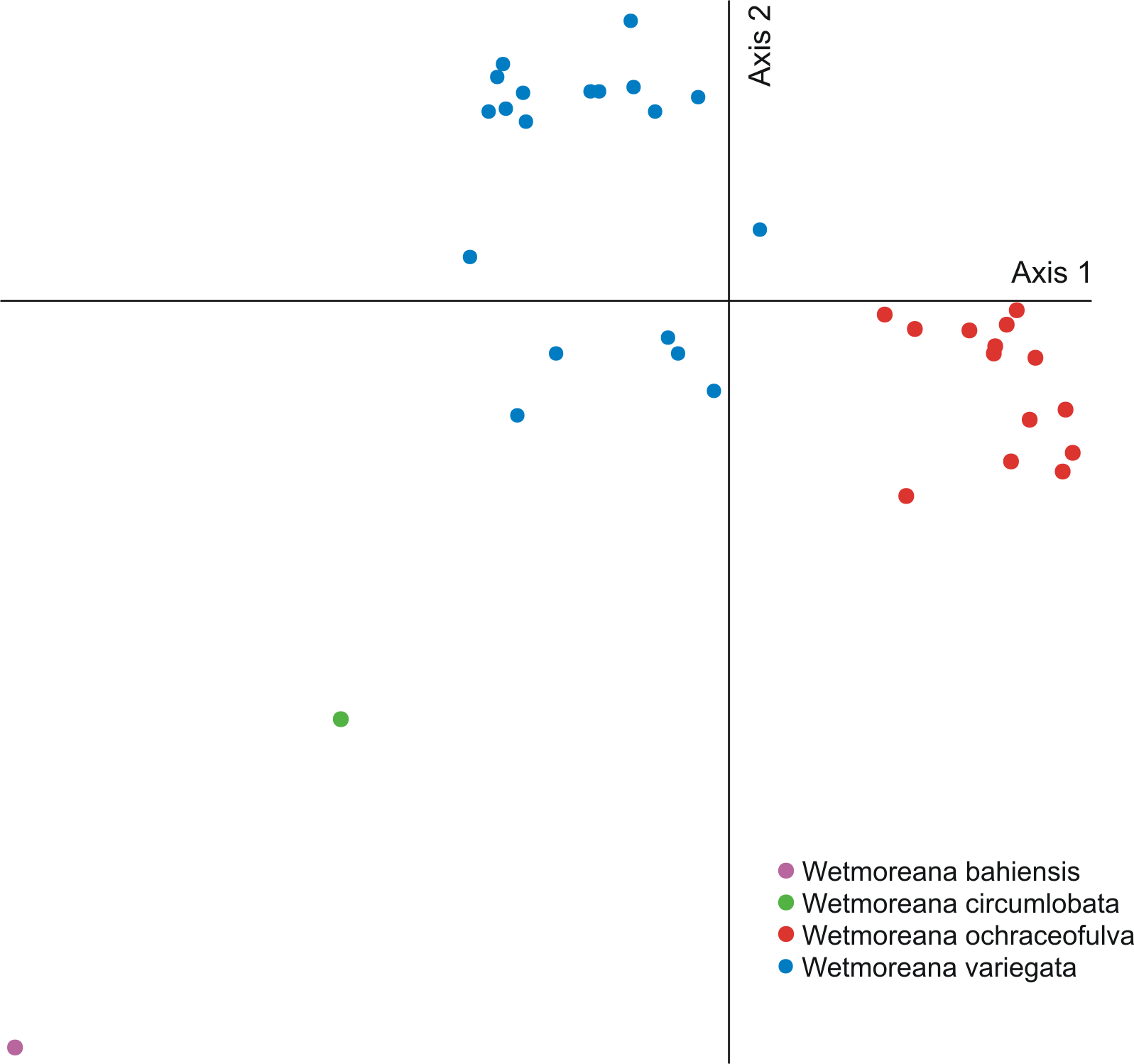

Supplement: Supplementary file 14 — Additional file 14: Figure S7. PCA ordination based on 46 phenotypic characters of the Wetmoreana ochraceofulva clade defined by PBPB-MP. [file 43008_2024_140_MOESM14_ESM.tif]

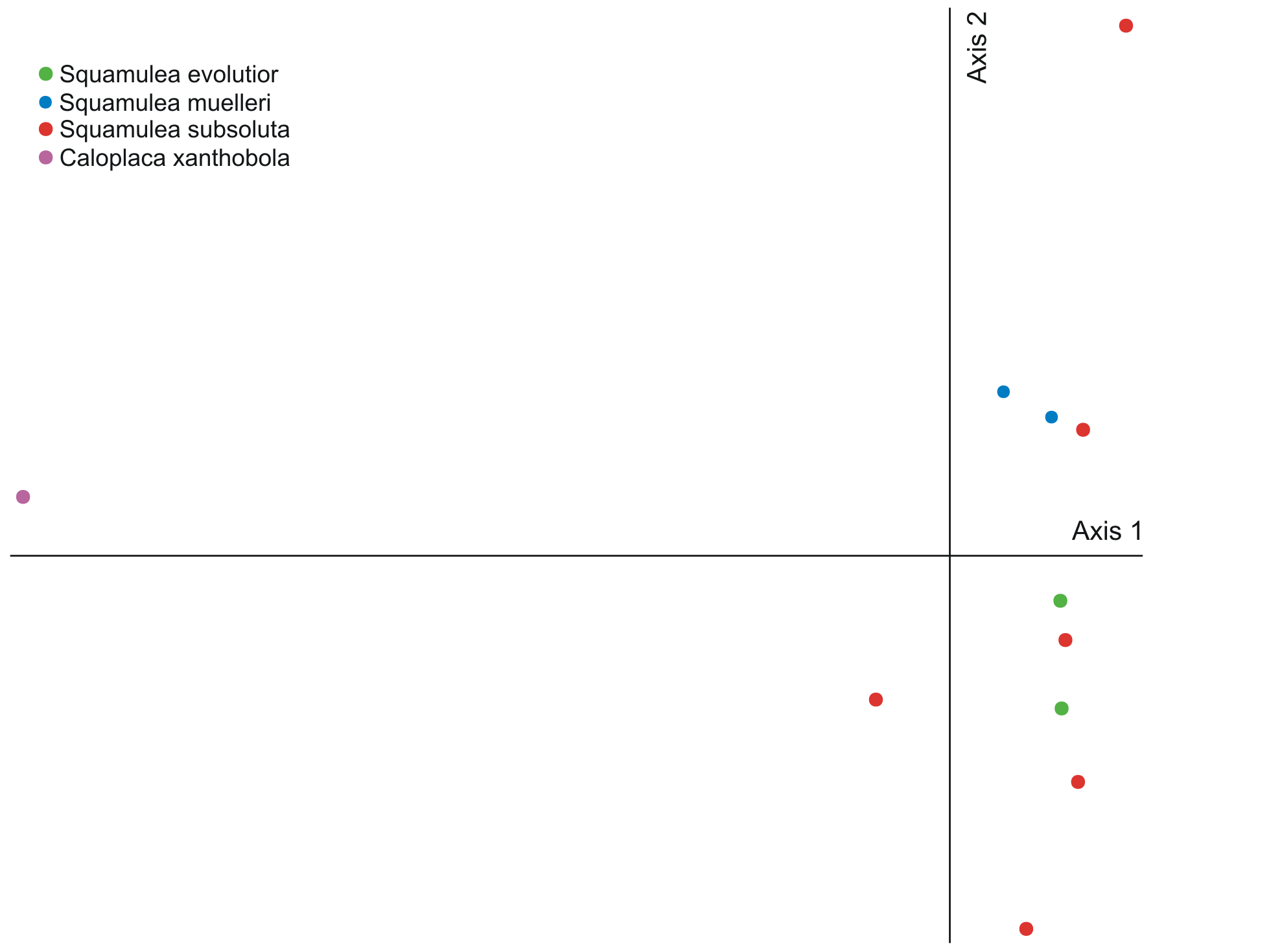

Supplement: Supplementary file 15 — Additional file 15: Figure S8. PCA ordination based on 32 phenotypic characters of the Squamulea subsoluta clade defined by PBPB-ML. [file 43008_2024_140_MOESM15_ESM.tif]

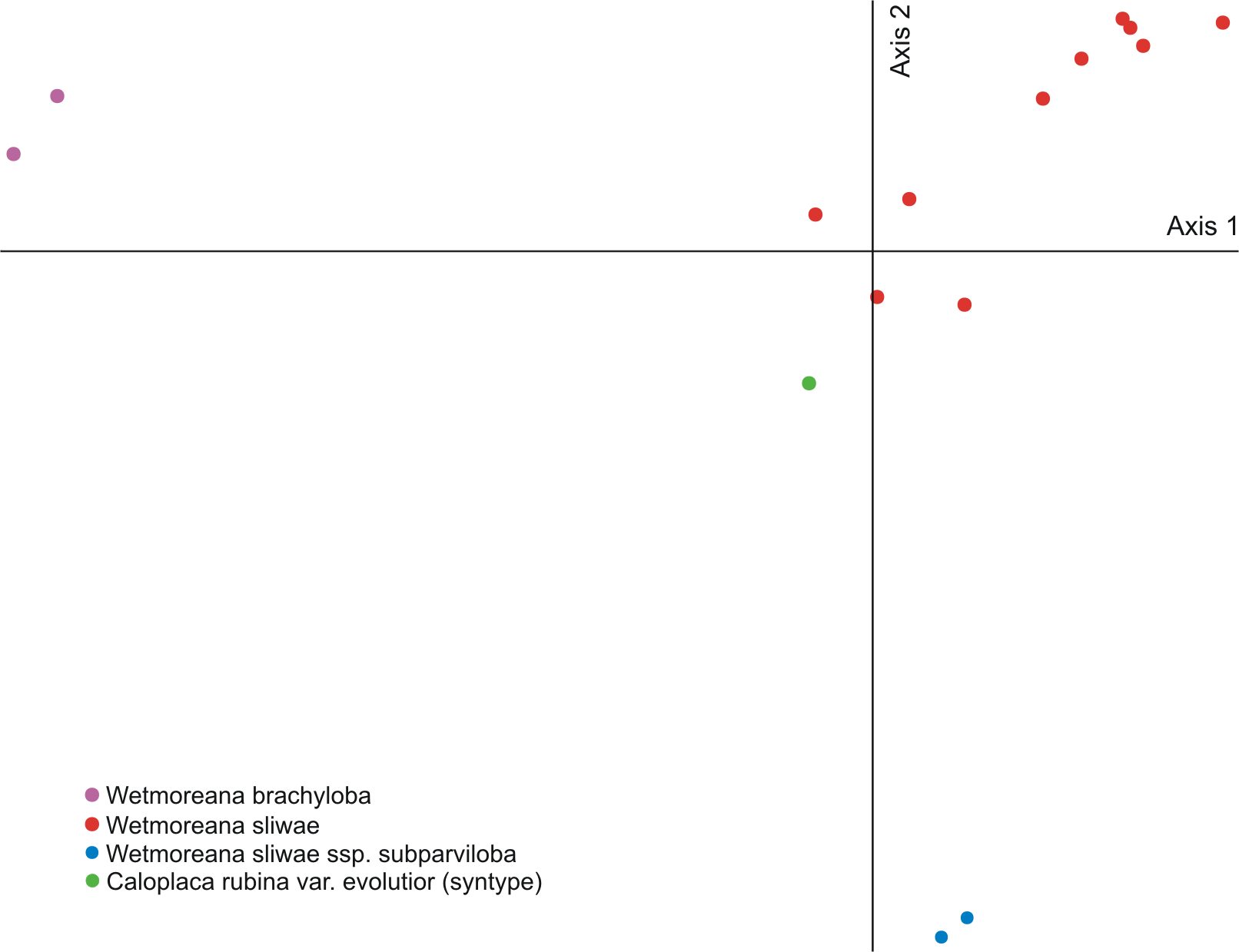

Supplement: Supplementary file 16 — Additional file 16: Figure S9. PCA ordination based on 36 phenotypic characters of the Wetmoreana sliwae clade defined by PBPB-ML. [file 43008_2024_140_MOESM16_ESM.tif]

**A**

PBPB, MP

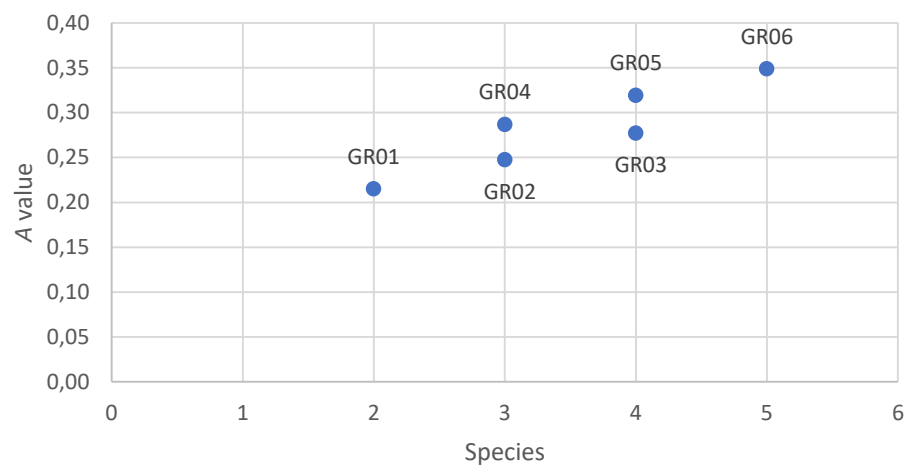**B**

PBPB, MP, without apothecia

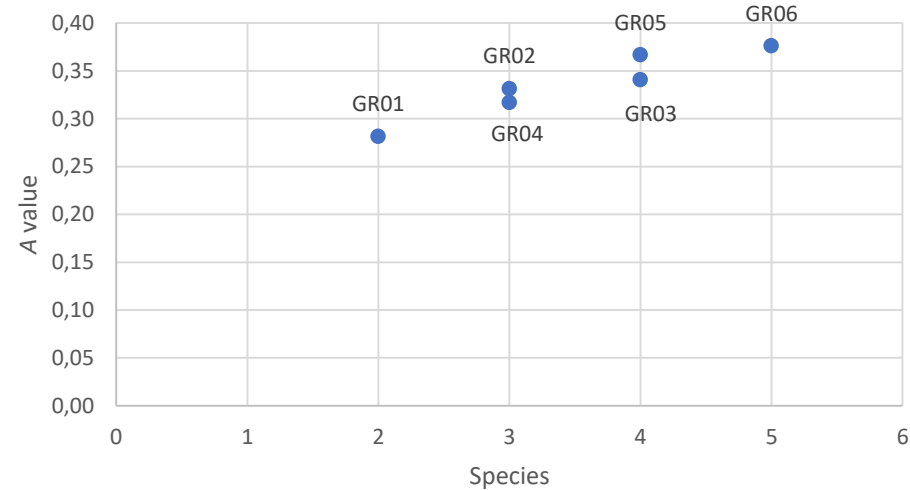**C**

DNA

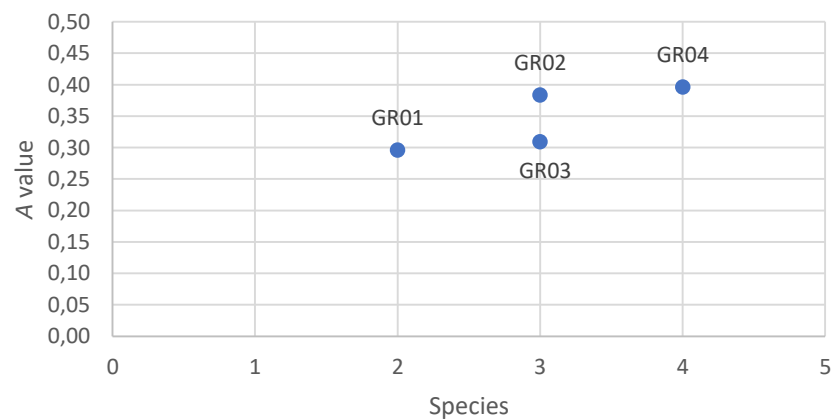**D**

DNA, without apothecia

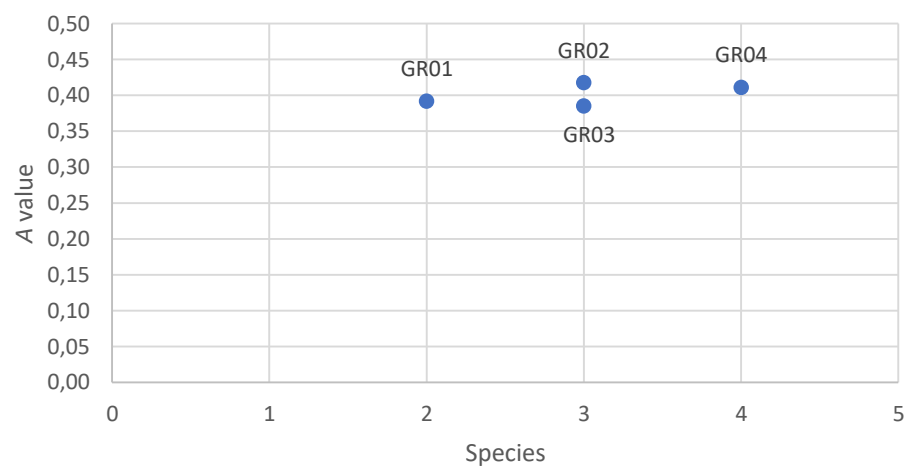

Supplement: Supplementary file 17 — Additional file 17: Figure S10. The comparison between the ochraceofulva /variegata and sliwae /subparviloba /brachyloba complexes using MRPP based on PBPB-MP (A, B) or molecular (C, D) defined clades, and for complete (A, C) and incomplete (without apothecia, B, D) datasets. The analyzed groups are as follow in case of PBPB-MP: GR01: ochraceofulva s.lat. vs. sliwae s.lat., GR02: ochraceofulva s.lat. vs. sliwae s.lat./brachyloba, GR03: ochraceofulva s.lat. vs. sliwae/subparviloba/brachyloba, GR04: ochraceofulva/variegata vs. sliwae s.lat., GR05: ochraceofulva/variegata vs. sliwae s.lat./brachyloba, GR06: ochraceofulva/variegata vs. sliwae/subparviloba/brachyloba. In case of DNA: GR01: ochraceofulva s.lat. vs. sliwae s.lat., GR02: ochraceofulva s.lat. vs. sliwae/subparviloba, GR03: ochraceofulva/variegata vs. sliwae s.lat., GR04: ochraceofulva/variegata vs. sliwae/subparviloba. [file 43008_2024_140_MOESM17_ESM.pdf]

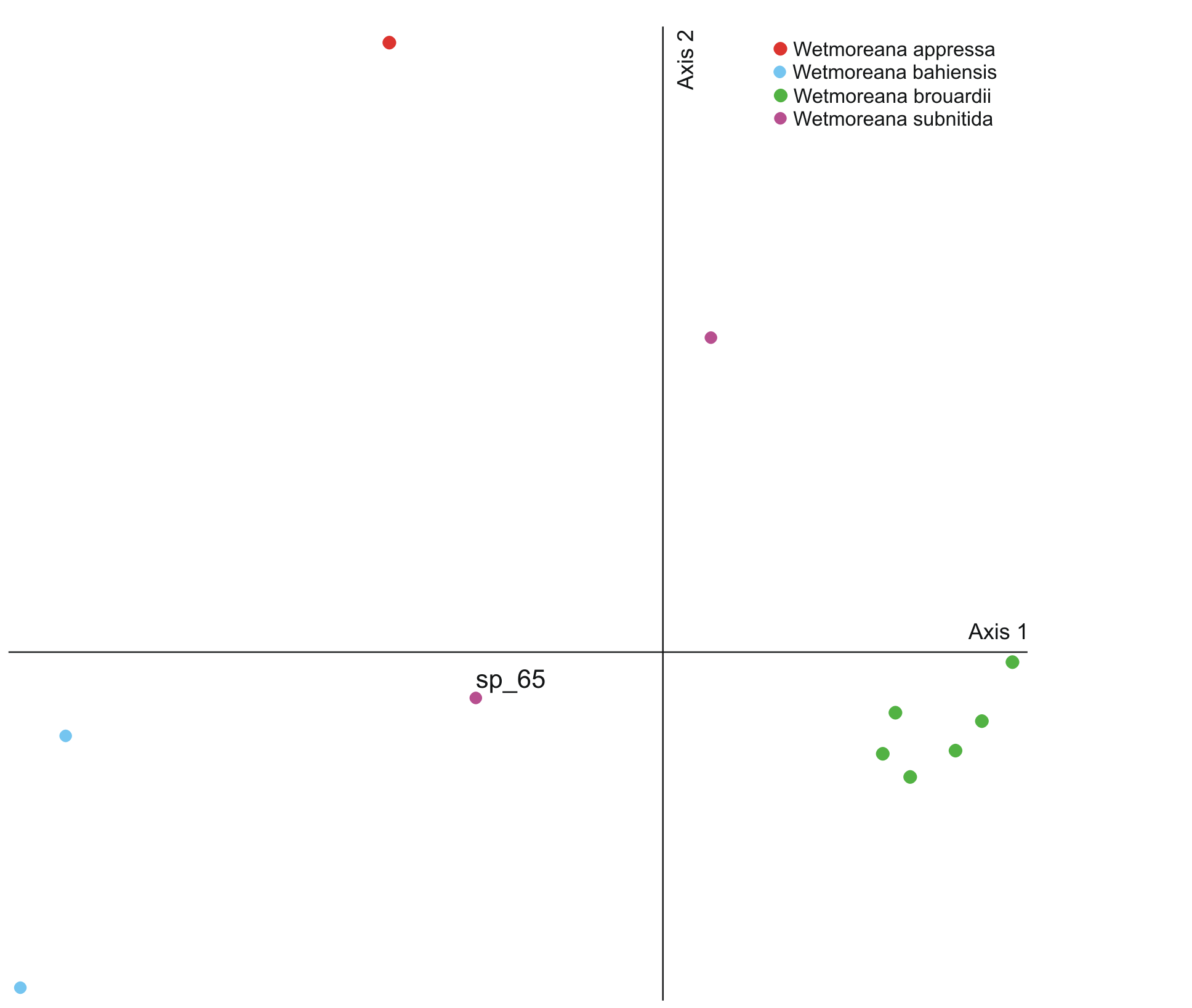

Supplement: Supplementary file 18 — Additional file 18: Figure S11. PCA ordination of the Wetmoreana brouardii complex based on 34 phenotypic characters of 11 species. [file 43008_2024_140_MOESM18_ESM.tif]
