# Supplementary material for: Quantitative integrative taxonomy informs species delimitation in Teloschistaceae (lichenized Ascomycota): the genus Wetmoreana as a case study
Source: IMA Fungus. 2024 Apr 1;15:9. doi: 10.1186/s43008-024-00140-1 (PMC11225190; doi:10.1186/s43008-024-00140-1)
Supplement: Supplementary file 12 — Additional file 12: Table S3. PBPB results presented as classification table. Placements of query specimens according ML and MP weighting, with bootstrap support (BS) indicated. [file 43008_2024_140_MOESM12_ESM.docx]

Additional file 12: Table S3. PBPB results presented as classification table. Placements of query specimens according ML and MP weighting, with bootstrap support (BS) indicated.

| **Weighting** | **Query specimen** | **Node** | **Placement** | **BP** | **Comments** |
| --- | --- | --- | --- | --- | --- |
| ML | Caloplaca_fernandeziana_106 | I61 | Caloplaca_fernandeziana_114[I61] | 100 | ***Caloplaca* s.lat.** |
| MP | Caloplaca_fernandeziana_106 | I61 | Caloplaca_fernandeziana_114[I61] | 100 |  |
| ML | Caloplaca_fernandeziana_84 | I61 | Caloplaca_fernandeziana_114[I61] | 100 |  |
| MP | Caloplaca_fernandeziana_84 | I61 | Caloplaca_fernandeziana_114[I61] | 100 |  |
| ML | Caloplaca_fernandeziana_85 | I61 | Caloplaca_fernandeziana_114[I61] | 100 |  |
| MP | Caloplaca_fernandeziana_85 | I61 | Caloplaca_fernandeziana_114[I61] | 100 |  |
| ML | Caloplaca_fernandeziana_var-validior-type_83 | I61 | Caloplaca_fernandeziana_114[I61] | 100 |  |
| MP | Caloplaca_fernandeziana_var-validior-type_83 | I61 | Caloplaca_fernandeziana_114[I61] | 100 |  |
| ML | Cinnabaria_boliviana_82 | I57 | Cinnabaria_boliviana-type_81[I57] | 100 | ***Cinnabaria*** |
| MP | Cinnabaria_boliviana_82 | I57 | Cinnabaria_boliviana-type_81[I57] | 100 |  |
| ML | Gyalolechia_gomerana_87 | I60 | Gyalolechia_gomerana_115[I60] | 100 | ***Gyalolechia*** |
| MP | Gyalolechia_gomerana_87 | I60 | Gyalolechia_gomerana_115[I60] | 100 |  |
| ML | Gyalolechia_gomerana_88 | I60 | Gyalolechia_gomerana_115[I60] | 100 |  |
| MP | Gyalolechia_gomerana_88 | I60 | Gyalolechia_gomerana_115[I60] | 100 |  |
| ML | Teuvoahtiana_altoandina-type_91 | I64 | Teuvoahtiana_altoandina_119 | 100 | ***Teuvoahtiana*** |
| MP | Teuvoahtiana_altoandina-type_91 | I64 | Teuvoahtiana_altoandina_119[I64] | 100 |  |
| ML | Teuvoahtiana_altoandina_92 | I62 | Calogaya biatorina 127 & Calogaya pusilla 126 | 95 | ***Teuvoahtiana*/**  ***Calogaya*** |
| ML | Teuvoahtiana_altoandina_92 | I64 | Teuvoahtiana_altoandina_119 | 5 |  |
| MP | Teuvoahtiana_altoandina_92 | I62 | Calogaya_biatorina_127[I73]/Calogaya_pusilla_126[I74] | 5 |  |
| MP | Teuvoahtiana_altoandina_92 | I64 | Teuvoahtiana_altoandina_119[I64] | 95 |  |
| ML | Caloplaca_xanthobola-type_101 | I70 | Squamulea_subsoluta_117[I70] | 100 | ***Squamulea*** |
| MP | Caloplaca_xanthobola-type_101 | I18 | Wetmoreana_sliwae_25[I18] | 5 |  |
| MP | Caloplaca_xanthobola-type_101 | I70 | Squamulea_subsoluta_117[I70] | 43 |  |
| MP | Caloplaca_xanthobola-type_101 | I9 | Wetmoreana_sliwae_24[I9] | 52 |  |
| ML | Caloplaca_rubina_var-evolutior-type_107 | I69 | Squamulea_subsoluta_118[I71] | 100 |  |
| MP | Caloplaca_rubina_var-evolutior-type_107 | I68 | Squamulea_parviloba_116[I68] | 1 |  |
| MP | Caloplaca_rubina_var-evolutior-type_107 | I69 | Squamulea_subsoluta_118[I71] | 69 |  |
| MP | Caloplaca_rubina_var-evolutior-type_107 | I70 | Squamulea_subsoluta_117[I70] | 18 |  |
| MP | Caloplaca_rubina_var-evolutior-type_107 | I71 | Squamulea_subsoluta_118[I71] | 12 |  |
| ML | Caloplaca_rubina-type_108 | I69 | Squamulea_subsoluta_118[I71] | 100 |  |
| MP | Caloplaca_rubina-type_108 | I68 | Squamulea_parviloba_116[I68] | 15 |  |
| MP | Caloplaca_rubina-type_108 | I69 | Squamulea_subsoluta_118[I71] | 63 |  |
| MP | Caloplaca_rubina-type_108 | I70 | Squamulea_subsoluta_117[I70] | 15 |  |
| MP | Caloplaca_rubina-type_108 | I71 | Squamulea_subsoluta_118[I71] | 7 |  |
| ML | Squamulea_flakusii_73 | I65 | Squamulea_flakusii-type_72[I66] | 96 |  |
| ML | Squamulea_flakusii_73 | I66 | Squamulea_flakusii-type_72[I66] | 4 |  |
| MP | Squamulea_flakusii_73 | I65 | Squamulea_flakusii-type_72[I66] | 56 |  |
| MP | Squamulea_flakusii_73 | I66 | Squamulea_flakusii-type_72[I66] | 44 |  |
| ML | Squamulea_flakusii_74 | I66 | Squamulea_flakusii-type_72[I66] | 100 |  |
| MP | Squamulea_flakusii_74 | I66 | Squamulea_flakusii-type_72[I66] | 96 |  |
| MP | Squamulea_flakusii_74 | I67 | Squamulea_parviloba_116[I68] | 4 |  |
| ML | Squamulea_parviloba_90 | I68 | Squamulea_parviloba_116[I68] | 100 |  |
| MP | Squamulea_parviloba_90 | I68 | Squamulea_parviloba_116[I68] | 100 |  |
| ML | Squamulea_parviloba-type_89 | I68 | Squamulea_parviloba_116[I68] | 100 |  |
| MP | Squamulea_parviloba-type_89 | I68 | Squamulea_parviloba_116[I68] | 100 |  |
| ML | Squamulea_subsoluta_110 | I70 | Squamulea_subsoluta_117[I70] | 100 |  |
| MP | Squamulea_subsoluta_110 | I70 | Squamulea_subsoluta_117[I70] | 100 |  |
| ML | Squamulea_subsoluta_78 | I70 | Squamulea_subsoluta_117[I70] | 89 |  |
| ML | Squamulea_subsoluta_78 | I71 | Squamulea_subsoluta_118[I71] | 11 |  |
| MP | Squamulea_subsoluta_78 | I70 | Squamulea_subsoluta_117[I70] | 100 |  |
| ML | Squamulea_subsoluta_79 | I70 | Squamulea_subsoluta_117[I70] | 100 |  |
| MP | Squamulea_subsoluta_79 | I70 | Squamulea_subsoluta_117[I70] | 96 |  |
| MP | Squamulea_subsoluta_79 | I71 | Squamulea_subsoluta_118[I71] | 4 |  |
| ML | Squamulea_subsoluta_80 | I70 | Squamulea_subsoluta_117[I70] | 100 |  |
| MP | Squamulea_subsoluta_80 | I70 | Squamulea_subsoluta_117[I70] | 100 |  |
| ML | Caloplaca_cf-tucumanensis_99 | I20 | Wetmoreana_subparviloba-type_35[I21] | 1 |  |
| ML | Caloplaca_cf-tucumanensis_99 | I68 | Squamulea_parviloba_116[I68] | 99 |  |
| MP | Caloplaca_cf-tucumanensis_99 | I68 | Squamulea_parviloba_116[I68] | 100 |  |
| ML | Caloplaca_muelleri_105 | I70 | Squamulea_subsoluta_117[I70] | 100 |  |
| MP | Caloplaca_muelleri_105 | I68 | Squamulea_parviloba_116[I68] | 79 |  |
| MP | Caloplaca_muelleri_105 | I70 | Squamulea_subsoluta_117[I70] | 21 |  |
| ML | Caloplaca_muelleri-type_102 | I70 | Squamulea_subsoluta_117[I70] | 100 |  |
| MP | Caloplaca_muelleri-type_102 | I68 | Squamulea_parviloba_116[I68] | 83 |  |
| MP | Caloplaca_muelleri-type_102 | I70 | Squamulea_subsoluta_117[I70] | 17 |  |
| ML | Caloplaca_bahiensis_63 | I0 | Wetmoreana_brouardii_128[I0] | 90 | ***Wetmoreana*** |
| ML | Caloplaca_bahiensis_63 | I35 | Wetmoreana_variegata-type_1[I35] | 10 |  |
| MP | Caloplaca_bahiensis_63 | I35 | Wetmoreana_variegata-type_1[I35] | 100 |  |
| ML | Caloplaca_bahiensis_64 | I0 | Wetmoreana_brouardii_128[I0] | 83 |  |
| ML | Caloplaca_bahiensis_64 | I36 | Wetmoreana_variegata_2[I36] | 16 |  |
| ML | Caloplaca_bahiensis_64 | I37 | Wetmoreana_variegata_4[I37] | 1 |  |
| MP | Caloplaca_bahiensis_64 | I0 | Wetmoreana_brouardii_128[I0] | 1 |  |
| MP | Caloplaca_bahiensis_64 | I36 | Wetmoreana_variegata_2[I36] | 63 |  |
| MP | Caloplaca_bahiensis_64 | I37 | Wetmoreana_variegata_4[I37] | 1 |  |
| MP | Caloplaca_bahiensis_64 | I49 | Wetmoreana_appressa_120[I49] | 35 |  |
| ML | Callopisma_brachylobum_32 | I25 | Wetmoreana_rubra-type_38[I43] | 84 |  |
| ML | Callopisma_brachylobum_32 | I26 | Caloplaca_ochraceofulva_60[I41] | 2 |  |
| ML | Callopisma_brachylobum_32 | I35 | Wetmoreana_variegata-type_1[I35] | 14 |  |
| MP | Callopisma_brachylobum_32 | I25 | Wetmoreana_rubra-type_38[I43] | 15 |  |
| MP | Callopisma_brachylobum_32 | I35 | Wetmoreana_variegata-type_1[I35] | 1 |  |
| MP | Callopisma_brachylobum_32 | I36 | Wetmoreana_variegata_2[I36] | 84 |  |
| ML | Wetmoreana_appressa-type_66 | I0 | Wetmoreana_brouardii_128[I0] | 74 |  |
| ML | Wetmoreana_appressa-type_66 | I35 | Wetmoreana_variegata-type_1[I35] | 11 |  |
| ML | Wetmoreana_appressa-type_66 | I45 | Wetmoreana_decipioides-type_70[I47]/Wetmoreana_appressa_120[I49] | 3 |  |
| ML | Wetmoreana_appressa-type_66 | I49 | Wetmoreana_appressa_120[I49] | 12 |  |
| MP | Wetmoreana_appressa-type_66 | I35 | Wetmoreana_variegata-type_1[I35] | 48 |  |
| MP | Wetmoreana_appressa-type_66 | I45 | Wetmoreana_decipioides-type_70[I47]/Wetmoreana_appressa_120[I49] | 1 |  |
| MP | Wetmoreana_appressa-type_66 | I49 | Wetmoreana_appressa_120[I49] | 51 |  |
| ML | Wetmoreana_brouardii_43 | I0 | Wetmoreana_brouardii_128[I0] | 100 |  |
| MP | Wetmoreana_brouardii_43 | I0 | Wetmoreana_brouardii_128[I0] | 100 |  |
| ML | Wetmoreana_brouardii_44 | I0 | Wetmoreana_brouardii_128[I0] | 100 |  |
| MP | Wetmoreana_brouardii_44 | I0 | Wetmoreana_brouardii_128[I0] | 100 |  |
| ML | Wetmoreana_brouardii_45 | I0 | Wetmoreana_brouardii_128[I0] | 100 |  |
| MP | Wetmoreana_brouardii_45 | I0 | Wetmoreana_brouardii_128[I0] | 100 |  |
| ML | Wetmoreana_brouardii_46 | I0 | Wetmoreana_brouardii_128[I0] | 100 |  |
| MP | Wetmoreana_brouardii_46 | I0 | Wetmoreana_brouardii_128[I0] | 100 |  |
| ML | Wetmoreana_brouardii_47 | I0 | Wetmoreana_brouardii_128[I0] | 100 |  |
| MP | Wetmoreana_brouardii_47 | I0 | Wetmoreana_brouardii_128[I0] | 100 |  |
| ML | Callopisma_subnitidum_65 | I0 | Wetmoreana_brouardii_128[I0] | 100 |  |
| MP | Callopisma_subnitidum_65 | I0 | Wetmoreana_brouardii_128[I0] | 100 |  |
| ML | Callopisma_subnitidum-syntype_71 | I0 | Wetmoreana_brouardii_128[I0] | 100 |  |
| MP | Callopisma_subnitidum-syntype_71 | I0 | Wetmoreana_brouardii_128[I0] | 100 |  |
| ML | Wetmoreana_sliwae_28 | I19 | Wetmoreana_sliwae_27[I23] | 6 |  |
| ML | Wetmoreana_sliwae_28 | I20 | Wetmoreana_subparviloba-type_35[I21] | 14 |  |
| ML | Wetmoreana_sliwae_28 | I23 | Wetmoreana_sliwae_27[I23] | 80 |  |
| MP | Wetmoreana_sliwae_28 | I20 | Wetmoreana_subparviloba-type_35[I21] | 82 |  |
| MP | Wetmoreana_sliwae_28 | I23 | Wetmoreana_sliwae_27[I23] | 18 |  |
| ML | Wetmoreana_sliwae_29 | I18 | Wetmoreana_sliwae_25[I18] | 100 |  |
| MP | Wetmoreana_sliwae_29 | I18 | Wetmoreana_sliwae_25[I18] | 100 |  |
| ML | Wetmoreana_sliwae_37 | I14 | Wetmoreana_sliwae-type_21[I14] | 100 |  |
| MP | Wetmoreana_sliwae_37 | I14 | Wetmoreana_sliwae-type_21[I14] | 100 |  |
| ML | Caloplaca_brachyloba_33 | I8 | Wetmoreana_sliwae_24[I9] | 100 |  |
| MP | Caloplaca_brachyloba_33 | I8 | Wetmoreana_sliwae_24[I9] | 100 |  |
| ML | Caloplaca_brachyloba-type_103 | I8 | Wetmoreana_sliwae_24[I9] | 100 |  |
| MP | Caloplaca_brachyloba-type_103 | I8 | Wetmoreana_sliwae_24[I9] | 100 |  |
| ML | Caloplaca_rubina_var-evolutior-syntype_96 | I23 | Wetmoreana_sliwae_27[I23] | 100 |  |
| MP | Caloplaca_rubina_var-evolutior-syntype_96 | I14 | Wetmoreana_sliwae-type_21[I14] | 2 |  |
| MP | Caloplaca_rubina_var-evolutior-syntype_96 | I20 | Wetmoreana_subparviloba-type_35[I21] | 93 |  |
| MP | Caloplaca_rubina_var-evolutior-syntype_96 | I23 | Wetmoreana_sliwae_27[I23] | 5 |  |
| ML | Wetmoreana_texana_67 | I6 | Wetmoreana_texana_124[I6] | 100 |  |
| MP | Wetmoreana_texana_67 | I5 | Wetmoreana_texana_124[I6] | 100 |  |
| ML | Wetmoreana_texana_68 | I6 | Wetmoreana_texana_124[I6] | 100 |  |
| MP | Wetmoreana_texana_68 | I5 | Wetmoreana_texana_124[I6] | 2 |  |
| MP | Wetmoreana_texana_68 | I6 | Wetmoreana_texana_124[I6] | 98 |  |
| ML | Wetmoreana_texana_69 | I7 | Wetmoreana_texana_125[I7] | 100 |  |
| MP | Wetmoreana_texana_69 | I7 | Wetmoreana_texana_125[I7] | 100 |  |
| ML | Wetmoreana_rubra_39 | I43 | Wetmoreana_rubra-type_38[I43] | 100 |  |
| MP | Wetmoreana_rubra_39 | I43 | Wetmoreana_rubra-type_38[I43] | 100 |  |
| ML | Wetmoreana_rubra_40 | I43 | Wetmoreana_rubra-type_38[I43] | 100 |  |
| MP | Wetmoreana_rubra_40 | I43 | Wetmoreana_rubra-type_38[I43] | 100 |  |
| ML | Wetmoreana_rubra_41 | I43 | Wetmoreana_rubra-type_38[I43] | 100 |  |
| MP | Wetmoreana_rubra_41 | I43 | Wetmoreana_rubra-type_38[I43] | 100 |  |
| ML | Wetmoreana_rubra_42 | I43 | Wetmoreana_rubra-type_38[I43] | 100 |  |
| MP | Wetmoreana_rubra_42 | I43 | Wetmoreana_rubra-type_38[I43] | 100 |  |
| ML | Caloplaca_chapadensis-type_97 | I0 | Wetmoreana_brouardii_128[I0] | 2 |  |
| ML | Caloplaca_chapadensis-type_97 | I43 | Wetmoreana_rubra-type_38[I43] | 98 |  |
| MP | Caloplaca_chapadensis-type_97 | I43 | Wetmoreana_rubra-type_38[I43] | 100 |  |
| ML | Callopisma_subnitidum-type_49 | I42 | Caloplaca_ochraceofulva_55[I42] | 100 |  |
| MP | Callopisma_subnitidum-type_49 | I42 | Caloplaca_ochraceofulva_55[I42] | 100 |  |
| ML | Caloplaca_ochraceofulva_50 | I42 | Caloplaca_ochraceofulva_55[I42] | 100 |  |
| MP | Caloplaca_ochraceofulva_50 | I42 | Caloplaca_ochraceofulva_55[I42] | 100 |  |
| ML | Caloplaca_ochraceofulva_51 | I42 | Caloplaca_ochraceofulva_55[I42] | 100 |  |
| MP | Caloplaca_ochraceofulva_51 | I38 | Wetmoreana_variegata_8[I38] | 2 |  |
| MP | Caloplaca_ochraceofulva_51 | I42 | Caloplaca_ochraceofulva_55[I42] | 98 |  |
| ML | Caloplaca_ochraceofulva_52 | I42 | Caloplaca_ochraceofulva_55[I42] | 100 |  |
| MP | Caloplaca_ochraceofulva_52 | I42 | Caloplaca_ochraceofulva_55[I42] | 100 |  |
| ML | Caloplaca_ochraceofulva_53 | I42 | Caloplaca_ochraceofulva_55[I42] | 100 |  |
| MP | Caloplaca_ochraceofulva_53 | I42 | Caloplaca_ochraceofulva_55[I42] | 100 |  |
| ML | Caloplaca_ochraceofulva_54 | I42 | Caloplaca_ochraceofulva_55[I42] | 100 |  |
| MP | Caloplaca_ochraceofulva_54 | I38 | Wetmoreana_variegata_8[I38] | 28 |  |
| MP | Caloplaca_ochraceofulva_54 | I42 | Caloplaca_ochraceofulva_55[I42] | 72 |  |
| ML | Caloplaca_ochraceofulva_56 | I42 | Caloplaca_ochraceofulva_55[I42] | 100 |  |
| MP | Caloplaca_ochraceofulva_56 | I42 | Caloplaca_ochraceofulva_55[I42] | 100 |  |
| ML | Caloplaca_ochraceofulva_57 | I42 | Caloplaca_ochraceofulva_55[I42] | 100 |  |
| MP | Caloplaca_ochraceofulva_57 | I42 | Caloplaca_ochraceofulva_55[I42] | 100 |  |
| ML | Caloplaca_ochraceofulva_58 | I42 | Caloplaca_ochraceofulva_55[I42] | 100 |  |
| MP | Caloplaca_ochraceofulva_58 | I42 | Caloplaca_ochraceofulva_55[I42] | 100 |  |
| ML | Caloplaca_ochraceofulva_59 | I42 | Caloplaca_ochraceofulva_55[I42] | 100 |  |
| MP | Caloplaca_ochraceofulva_59 | I42 | Caloplaca_ochraceofulva_55[I42] | 100 |  |
| ML | Caloplaca_ochraceofulva_61 | I42 | Caloplaca_ochraceofulva_55[I42] | 100 |  |
| MP | Caloplaca_ochraceofulva_61 | I42 | Caloplaca_ochraceofulva_55[I42] | 100 |  |
| ML | Caloplaca_ochraceofulva_62 | I42 | Caloplaca_ochraceofulva_55[I42] | 100 |  |
| MP | Caloplaca_ochraceofulva_62 | I42 | Caloplaca_ochraceofulva_55[I42] | 100 |  |
| ML | Caloplaca_ochraceofulva-type_48 | I26 | Caloplaca_ochraceofulva_60[I41] | 24 |  |
| ML | Caloplaca_ochraceofulva-type_48 | I42 | Caloplaca_ochraceofulva_55[I42] | 76 |  |
| MP | Caloplaca_ochraceofulva-type_48 | I42 | Caloplaca_ochraceofulva_55[I42] | 100 |  |
| ML | Wetmoreana_variegata_10 | I32 | Wetmoreana_variegata_7[I32] | 3 |  |
| ML | Wetmoreana_variegata_10 | I42 | Caloplaca_ochraceofulva_55[I42] | 97 |  |
| MP | Wetmoreana_variegata_10 | I32 | Wetmoreana_variegata_7[I32] | 100 |  |
| ML | Wetmoreana_variegata_11 | I38 | Wetmoreana_variegata_8[I38] | 89 |  |
| ML | Wetmoreana_variegata_11 | I42 | Caloplaca_ochraceofulva_55[I42] | 11 |  |
| MP | Wetmoreana_variegata_11 | I32 | Wetmoreana_variegata_7[I32] | 100 |  |
| ML | Wetmoreana_variegata_12 | I31 | Wetmoreana_variegata_6[I33] | 93 |  |
| ML | Wetmoreana_variegata_12 | I39 | Wetmoreana_variegata_5[I39] | 7 |  |
| MP | Wetmoreana_variegata_12 | I39 | Wetmoreana_variegata_5[I39] | 100 |  |
| ML | Wetmoreana_variegata_13 | I32 | Wetmoreana_variegata_7[I32] | 100 |  |
| MP | Wetmoreana_variegata_13 | I32 | Wetmoreana_variegata_7[I32] | 100 |  |
| ML | Wetmoreana_variegata_14 | I38 | Wetmoreana_variegata_8[I38] | 100 |  |
| MP | Wetmoreana_variegata_14 | I39 | Wetmoreana_variegata_5[I39] | 100 |  |
| ML | Wetmoreana_variegata_15 | I37 | Wetmoreana_variegata_4[I37] | 94 |  |
| ML | Wetmoreana_variegata_15 | I39 | Wetmoreana_variegata_5[I39] | 6 |  |
| MP | Wetmoreana_variegata_15 | I32 | Wetmoreana_variegata_7[I32] | 100 |  |
| ML | Wetmoreana_variegata_16 | I35 | Wetmoreana_variegata-type_1[I35] | 98 |  |
| ML | Wetmoreana_variegata_16 | I37 | Wetmoreana_variegata_4[I37] | 2 |  |
| MP | Wetmoreana_variegata_16 | I37 | Wetmoreana_variegata_4[I37] | 100 |  |
| ML | Wetmoreana_variegata_17 | I26 | Caloplaca_ochraceofulva_60[I41] | 18 |  |
| ML | Wetmoreana_variegata_17 | I37 | Wetmoreana_variegata_4[I37] | 82 |  |
| MP | Wetmoreana_variegata_17 | I26 | Caloplaca_ochraceofulva_60[I41] | 36 |  |
| MP | Wetmoreana_variegata_17 | I29 | Wetmoreana_variegata_4[I37] | 2 |  |
| MP | Wetmoreana_variegata_17 | I31 | Wetmoreana_variegata_6[I33] | 3 |  |
| MP | Wetmoreana_variegata_17 | I32 | Wetmoreana_variegata_7[I32] | 1 |  |
| MP | Wetmoreana_variegata_17 | I37 | Wetmoreana_variegata_4[I37] | 58 |  |
| ML | Wetmoreana_variegata_18 | I32 | Wetmoreana_variegata_7[I32] | 6 |  |
| ML | Wetmoreana_variegata_18 | I37 | Wetmoreana_variegata_4[I37] | 94 |  |
| MP | Wetmoreana_variegata_18 | I32 | Wetmoreana_variegata_7[I32] | 100 |  |
| ML | Wetmoreana_variegata_20 | I32 | Wetmoreana_variegata_7[I32] | 37 |  |
| ML | Wetmoreana_variegata_20 | I37 | Wetmoreana_variegata_4[I37] | 63 |  |
| MP | Wetmoreana_variegata_20 | I32 | Wetmoreana_variegata_7[I32] | 100 |  |
| ML | Wetmoreana_variegata_3 | I29 | Wetmoreana_variegata_4[I37] | 1 |  |
| ML | Wetmoreana_variegata_3 | I34 | Wetmoreana_variegata_2[I36] | 90 |  |
| ML | Wetmoreana_variegata_3 | I35 | Wetmoreana_variegata-type_1[I35] | 5 |  |
| ML | Wetmoreana_variegata_3 | I36 | Wetmoreana_variegata_2[I36] | 2 |  |
| ML | Wetmoreana_variegata_3 | I37 | Wetmoreana_variegata_4[I37] | 2 |  |
| MP | Wetmoreana_variegata_3 | I29 | Wetmoreana_variegata_4[I37] | 9 |  |
| MP | Wetmoreana_variegata_3 | I34 | Wetmoreana_variegata_2[I36] | 3 |  |
| MP | Wetmoreana_variegata_3 | I35 | Wetmoreana_variegata-type_1[I35] | 36 |  |
| MP | Wetmoreana_variegata_3 | I36 | Wetmoreana_variegata_2[I36] | 39 |  |
| MP | Wetmoreana_variegata_3 | I37 | Wetmoreana_variegata_4[I37] | 13 |  |
| ML | Wetmoreana_variegata_9 | I32 | Wetmoreana_variegata_7[I32] | 100 |  |
| MP | Wetmoreana_variegata_9 | I32 | Wetmoreana_variegata_7[I32] | 100 |  |
